# Explainable random forest predictions of polyester biodegradability using high-throughput biodegradation data

**DOI:** 10.1039/d5sc05380c

**Published:** 2025-11-18

**Authors:** Philippa L. Jacob, Madeleine I. Parker, Daniel J. Keddie, Vincenzo Taresco, Steven M. Howdle, Jonathan D. Hirst

**Affiliations:** a School of Chemistry, University of Nottingham Nottingham NG7 2RD UK jonathan.hirst@nottingham.ac.uk

## Abstract

The development of new, biodegradable polyesters is becoming increasingly important as legislative and consumer drivers push society towards more sustainable polymers. One key bottleneck in the development of biodegradable polyesters is the slow nature of biodegradability testing, which can often take weeks to months. High-throughput screening assays serve as a rapid tool to determine the biodegradability of materials quickly, without the need for lengthy, expensive testing. When combined with machine learning, the data generated by high-throughput assays can be exploited to predict the properties of other similar materials. Here, we report the development of a high-throughput enzymatic biodegradation assay, which has been used to determine the biodegradability of 48 polyesters. Using data generated from the assay to train a predictive model, we can predict the biodegradability of polyesters using an explainable random forest model with 71% accuracy. Transfer learning and model chaining were investigated as routes to improve the model predictions by exploiting existing literature data. SHAP analysis gives insight into the beneficial structural features of biodegradable polyesters. This understanding can be applied in the development of future biodegradable polyesters.

## Abstract 2

A new high-throughput enzymatic biodegradation assay has been developed, and the data have been used to train a machine learning model to predict polyester biodegradability and explain how structural features influence biodegradability.

## Graphical abstract

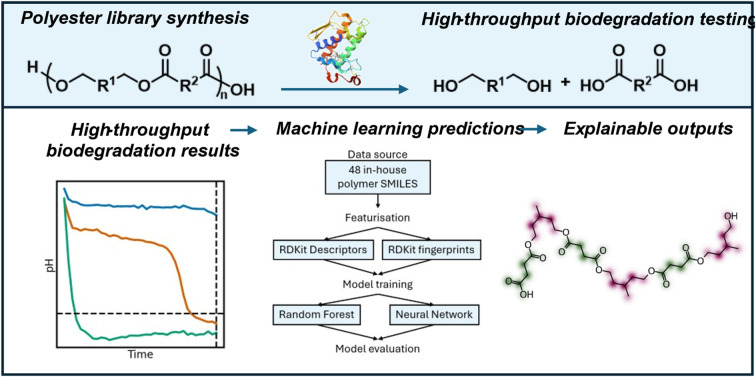

## Introduction

The non-degradable nature of many commonly used polymers has a detrimental impact on the environment, leading to the accumulation of waste in oceans, landfills, and other ecosystems.^1^ This build-up contributes to water pollution, habitat destruction, and risks to human health. One strategy to mitigate pollution from plastics as well as polymers in liquid formulations (PLFs) is the development of biodegradable polymers, which break down into their constituent monomers under natural conditions, reducing long-term waste accumulation.^2–4^

The United Nations Sustainable Development Goals (SDGs) were announced in 2015 to address global threats and strive towards a more sustainable future.^5^ Although pollution from polymers is only mentioned as part of one goal – life on land (SDG 14, item 14.1.1b), this goal is intrinsically intertwined with several other goals including SDGs 11 (sustainable cities and communities), 12 (responsible consumption and production) and 13 (climate action).^6^ The motivation for a more sustainable future, driven by these SDGs, has resulted in an increase in consumer awareness and more substantive government legislation. This, in turn, has increased research and development into biodegradable polymers, with several large-scale applications now emerging in food packaging and biomedical applications.^7^ Standard biodegradation tests often require weeks of incubation and extensive data collection, making them both time- and resource-intensive. These limitations slow down the design and optimisation of new biodegradable materials.

Machine learning (ML) has gained traction as a powerful tool for predicting polymer properties.^8–13^ Fransen *et al.* used machine learning to predict biodegradability, screening over 600 polyesters and polycarbonates using a clear-zone assay and trained a random forest (RF) model on the data, predicting biodegradability with 82% accuracy.^14^ A lack of consistent and available biodegradation data could be attributed to the slow progress in biodegradability prediction. The range of biodegradation screenings used in the literature means that biodegradation data often cannot be directly compared, making pattern recognition *via* ML difficult. Furthermore, access to raw data is often limited as data are frequently presented graphically in the literature. With little access to large data, the development of ML applications in this field has been stymied.

In this work, amphiphilic polyesters are of particular interest due to their application as surfactant-type molecules. These polymers are capable of self-assembly, similar to surfactants, and there are multiple examples of their use in drug delivery, personal care and as PLFs.^15,16^ In 2023, the Royal Society of Chemistry highlighted the unsustainable nature of many frequently used PLFs.^17^ Whilst amphiphilic polyesters may be one route towards more sustainable PLFs, it is important to consider their biodegradation profiles, as many polyesters are not biodegradable. With the publication of this report in 2023, our work is particularly timely and will provide a strong foundation for future research into biodegradable PLFs.

Herein, we report the development of a high-throughput accelerated biodegradation assay feeding into an RF model to predict the biodegradability of functional polyesters. A predictive model was trained from biodegradability data obtained from a library of polymers prepared in-house. We compared two different algorithms and feature representations during the model development, as well as assessing the applicability domain of the model to ensure reliable predictions. SHAP analysis^18^ has been used to examine feature importance, providing insights into structural features influencing biodegradability. Using a highly explainable model supports the rational design of biodegradable polymers and facilitates more efficient screening of new materials.

## Methods

### Polymer synthesis

All reagents were used as received without further purification. Details of reagent suppliers and purity can be found in the supplementary information (Table S1).

**Fig. 1:**
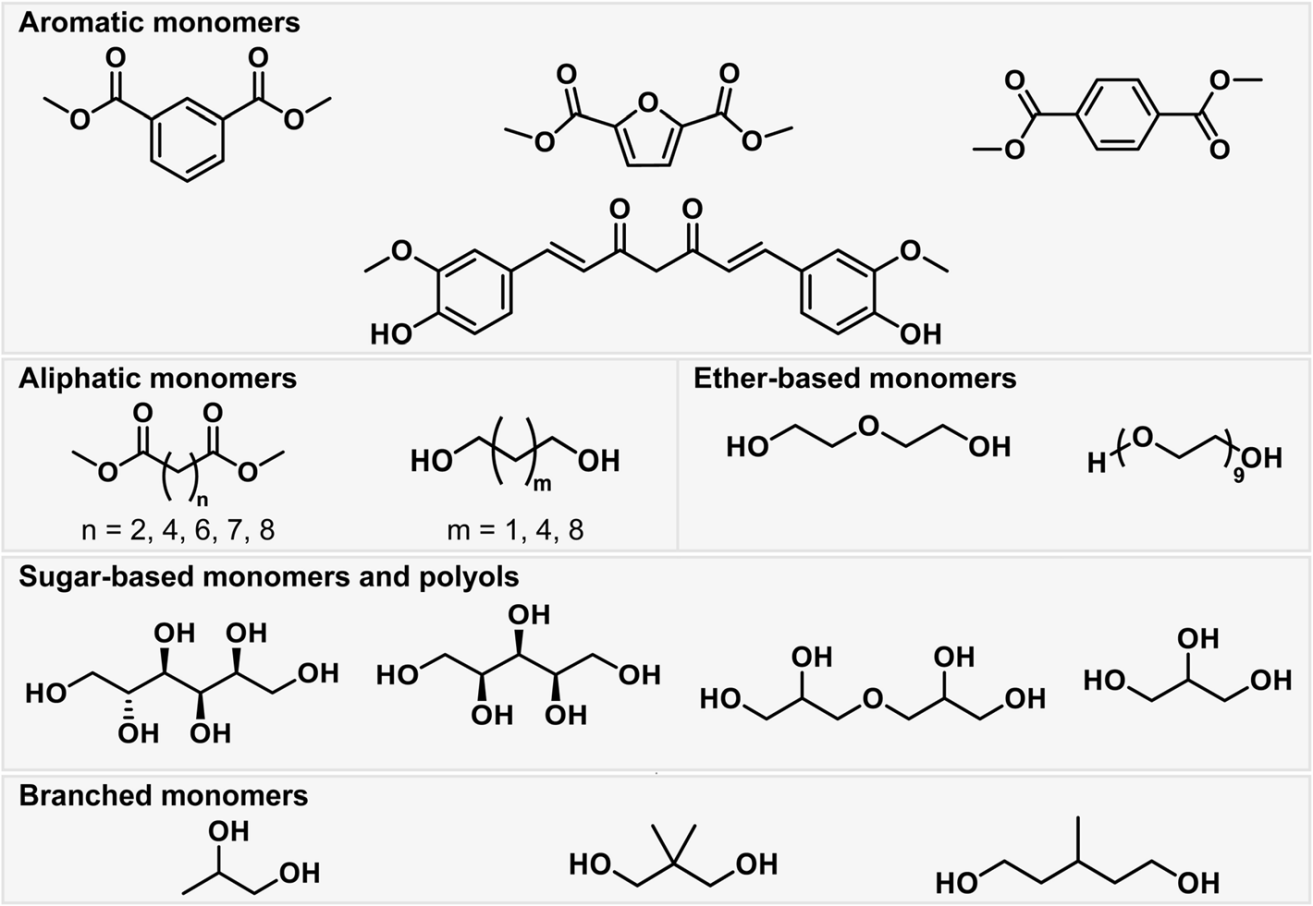
Examples of monomer types used in the synthesis of the library of polyesters that underwent biodegradability assessment.

To synthesise a library of amphiphilic polyesters, a range of monomers were chosen accordingly (Fig. 1). Previously reported literature on high-throughput polymer biodegradation focuses on non-water-soluble polymers with few pendant functionalities.^14^ The limited functionality of these literature polymers does not align with our aim of assessing the biodegradability of surfactant-type polymers for application in the PLF field. Therefore, we synthesised a new polymer library, better aligned to our research objectives. Using monomers including glycerol, diglycerol, sorbitol and xylitol, amphiphilic polyesters were successfully synthesised.

48 polyesters were synthesised *via* polycondensation or ring-opening polymerisation for biodegradation assessment (Table 1). Polycondensations were performed in bulk (solvent-free conditions) using equimolar ratios of diol:diacid/diester and potassium carbonate (K_2_CO_3_) catalyst (5 wt% w.r.t the total monomer mass) for 24 h.^15^ Specific molar amounts and reaction temperatures are detailed in Table S2.

**Table 1:** Monomer combinations, molar masses and biodegradability of polyesters

Polymer number	Monomer 1	Monomer 2	*M*_n_ (g mol^−1^)	*M*_w_ (g mol^−1^)	*Ð*	Biodegradable^b^
1	Isosorbide	Dimethyl furan-2,5-dicarboxylate	610	870	1.4	Yes
2	Sorbitol	Dimethyl succinate	n.d.	n.d.	n.d.	No
3	Sorbitol	Dimethyl suberate	510	4745	9.3	No
4	Sorbitol	Dimethyl azelate	680	3472	5.1	No
5	Xylitol	Dimethyl adipate	4160	7410	1.8	No
6	Curcumin	Dimethyl fumarate	1030	1270	1.2	No
7	Isosorbide	Dimethyl fumarate	1780	2895	1.6	Yes
8	Glycerol	Dimethyl suberate	1725	2225	1.3	Yes
9	1,3-Propanediol	Cyclobutane-1,1-dicarboxylic acid	470	580	1.2	Yes
10	PEG400	Dimethyl adipate	2065	3305	1.6	No
11	PEG400	Dimethyl furan-2,5-dicarboxylate	3045	4450	1.5	No
12^a^	1,2-Propanediol	Dimethyl isophthalate	2080	3545	1.7	No
13^a^	1,4-Cyclohexanedimethanol	Dimethyl terephthalate	n.d.	n.d.	n.d.	No
14^a^	3-Methyl-1,5-pentanediol	Dimethyl terephthalate	2795	4480	1.6	No
15^a^	1,10-Decanediol	Dimethyl terephthalate	n.d.	n.d.	n.d.	No
16^a^	1,6-Hexanediol	Dimethyl furan-2,5-dicarboxylate	n.d.	n.d.	n.d.	No
17^a^	2-Butene-1,4-diol	Dimethyl succinate	1345	3530	2.5	Yes
18^a^	3-Methyl-1,5-pentanediol	Dimethyl succinate	3120	6385	2.0	Yes
19^a^	1,10-Decanediol	Dimethyl succinate	915	2900	3.2	No
20^a^	2,2-Dimethyl-1,3-propanediol	Dimethyl adipate	1030	1620	1.6	No
21^a^	1,6-Hexanediol	Dimethyl adipate	6135	11 430	1.9	No
22^a^	Diethylene glycol	Dimethyl adipate	5835	10 860	1.8	Yes
23^a^	1,4-Cyclohexanedimethanol	Dimethyl sebacate	910	1880	2.1	No
24^a^	1,10-Decanediol	Dimethyl sebacate	871	1070	1.2	No
25^a^	1,6-Hexanediol	Dimethyl sebacate	7470	13 680	1.8	No
26^a^	Diethylene glycol	Dimethyl sebacate	2510	6260	2.5	Yes
27	Glycerol	Dimethyl furan-2,5-dicarboxylate	1480	2115	1.4	Yes
28	Glycerol	Dimethyl succinate	3130	5795	1.9	Yes
29	Glycerol	Dimethyl adipate	2465	3485	1.4	Yes
30	Glycerol	Dimethyl azelate	2490	3820	1.5	Yes
31	Glycerol	Dimethyl sebacate	2410	3291	1.4	Yes
32	Diglycerol	Dimethyl succinate	3310	4595	1.4	Yes
33	Diglycerol	Dimethyl adipate	3945	7045	1.8	No
34	Diglycerol	Dimethyl azelate	4405	9690	2.2	Yes
35	Diglycerol	Dimethyl furan-2,5-dicarboxylate	2210	3290	1.5	Yes
36	Diglycerol	Dimethyl azelate	1350	1665	1.2	Yes
37	Diglycerol	Dimethyl sebacate	3950	6330	1.6	Yes
38	Diglycerol	Dimethyl suberate	3295	4355	1.3	Yes
39	1,3-Propanediol	Dimethyl furan-2,5-dicarboxylate	n.d.	n.d.	n.d.	Yes
40^a^	1,3-Propanediol	Dimethyl succinate	1450	2785	1.9	Yes
41	PEG400	Dimethyl succinate	1950	3085	1.6	No
42	PEG400	Dimethyl suberate	1500	2105	1.4	Yes
43	PEG400	Dimethyl azelate	1340	1785	1.3	Yes
44	PEG400	Dimethyl sebacate	1970	3100	1.6	Yes
45^a^	ε-Caprolactone	—	9120	12 900	1.4	No
46^a^	δ-Valerolactone	—	6940	13 360	1.4	No
47^a^	Lactide	—	12 830	15 300	1.2	No
48^a^	Lactide	Glycolide	4990	14 690	2.9	Yes

a Polymers marked with an asterisk also appear in the literature dataset.^14^ Molar masses were determined using GPC, further details in SI.

b According to the in-house assay.

Ring-opening polymerisations of ε-caprolactone, δ-valerolactone and d,l-lactide were performed in bulk for 24 h using tin(ii) 2-ethylhexanoate (Sn(Oct)_2_) as a catalyst and benzyl alcohol as an initiator.^19^ Polymers were purified by precipitation from dichloromethane (DCM) into cold diethyl ether. Specific molar amounts and reaction temperatures are detailed in Table S3. Gel permeation chromatography (GPC) analysis of all polymers can be found in Table S4. Characterisation methods can also be found in the supplementary information.

### Biodegradation assay

Polymer (20 mg) was dissolved in dimethylsulfoxide (DMSO) (1 mL), and a 25 µL aliquot was solvent cast into a 96-well plate (Greiner, black, flat bottom, chimney). Where polymers were not completely soluble in DMSO, a suspension of polymer in DMSO was used. DMSO was removed *in vacuo* in a 50 °C vacuum oven for 24 h. Once dry, lipase from porcine pancreas (1 mg mL^−1^ solution in pH 8 buffer, 100 µL) and fluorescein solution (0.025 mM in pH 8 buffer, 70 µL) were added to the wells. Control experiments were run in the absence of enzyme and/or dye. Phosphate buffer (1 mM, pH 8) was freshly prepared for each assay.

As biodegradation takes place, diacid monomers are produced and these lower the pH of the solution. Fluorescence spectroscopy (TECAN Infinite M Plex) was used to monitor the pH of each polymer-containing well and hence monitor biodegradation (*λ*_excitation_ = 498 nm, *λ*_emission_ = 517 nm). A calibration curve relating the fluorescence intensity of fluorescein to pH was prepared by measuring the fluorescence of phosphate buffer solutions at regular intervals between pH 5.6–7.95 (Fig. S1). Between these pH values, the fluorescence of fluorescein can be related to pH.^20^ All biodegradation assays were run in at least duplicate. Polymers were considered biodegradable if a pH of less than 5.4, indicative of diacid formation, was measured after 18.5 h.

Not all polymers were soluble in the aqueous environment used in the assay. Indeed, a variety of degradation rates were observed whilst monitoring the pH using fluorescence spectroscopy, and this can be attributed to several factors including polyester solubility. It is anticipated that the differences in biodegradation rate and solubility have been somewhat mitigated by the longer timeframe used in the assay. Several polyesters reached the pH cutoff within shorter times, but a longer timeframe (18.5 h) was used to avoid bias in the assay towards faster-degrading polyesters.

### Data preparation

The in-house dataset comprising 48 polyesters was utilised for ML. To standardise the representation of polymer structures, each polymer was considered as a trimer. Each trimer was generated by computationally ‘polymerising’ the repeat unit of the corresponding polymer. This representation was chosen as it is theoretically the shortest sequence where RDKit fingerprints^21^ with the default maximum path length cannot capture an entire polymer backbone in a single bit. Each polymer in the dataset was represented by its Simplified Molecular Input Line Entry System (SMILES)^22^ string along with its biodegradability classification (biodegradable or non-biodegradable). Several generalisations have been made when using trimer representations, including the assumption that all polymers have one acid and one alcohol end group (for polymers made using polycondensation as well as ROP), as well as regular incorporation of asymmetric monomers into the polymer chain.

Polymer chain-end groups as well as internal ester linkages are considered within the trimer representation. Both of these moieties are reported to play a role in polyester biodegradation as chain ends are most susceptible to hydrolysis, while random scission of the polymer chain is responsible for the large reduction in polymer molar mass.^23^ This representation is also in agreement with the regulation on the registration, evaluation, authorisation and restriction of chemicals (REACH) definition of a polymer molecule: “a molecule that contains a sequence of at least 3 monomer units, which are covalently bound to at least one other monomer unit or other reactant.”^24^ This trimer representation was used as the primary structural input for feature extraction and modelling.

### Feature extraction

Two types of molecular features were considered: molecular descriptors and fingerprints. Molecular descriptors were computed using RDKit for each trimer, resulting in a feature set of 210 descriptors. These descriptors captured various structural, electronic, and physicochemical properties relevant to the polymer's biodegradability. A variance thresholding step was applied to remove descriptors with zero variance across the dataset, eliminating redundant features.

RDKit fingerprints were generated directly from the SMILES strings of the trimers. Parameters such as fingerprint length and maximum path length were tuned. To minimise bit collisions and enhance interpretability, longer fingerprint lengths and shorter maximum-paths were set. Polymers were treated as linear entities in their trimer representations, although in some cases, more branched structures may also be present.

### Model development and training

Two types of model were considered: RF and neural networks (NN), selected for their robustness and ability to handle high-dimensional feature spaces. The models were trained and validated using a nested cross-validation approach to ensure reliable performance estimation and hyperparameter optimisation. Stratified five-fold nested cross-validation was employed to maintain the class balance in both training and validation splits and to avoid overfitting, problems often observed when handling limited data.^25^ In the inner loop, hyperparameters were tuned, while the outer loop estimated the model's generalisation performance. Hyperparameter tuning was conducted over a predefined search space (Table 2) which included parameters related to both fingerprint generation, such as length and path size, and model configuration. Optimal hyperparameters were identified based on the highest accuracy achieved during the inner cross-validation loop.

**Table 2:** Hyperparameter search space

Hyperparameter	Search space
Fingerprint generation	
Fingerprint length	2048, 4096, 8192
Maximum path length	4, 5, 6

Random forest	
Number of trees	100, 200, 500
Maximum tree depth	2, 4, 6
Maximum number of features considered for each split	2, 5, 10

Neural network	
Hidden layer sizes	(8, 0), (16, 0), (8, 4), (16, 8)
Maximum number of iterations	500, 1000, 1500

After identifying the best hyperparameters, the final model was trained and evaluated using the outer loop of cross-validation. Performance metrics such as accuracy, precision, recall, F1 score, and receiver operating characteristic area under the curve (ROC-AUC) were calculated to assess model performance. The Brier score was used to evaluate model calibration.

### Statistical analysis

Statistical tests were employed to evaluate the significance of differences in model configurations and hyperparameter settings. The Friedman test, a non-parametric test, was used to compare multiple model configurations across cross-validation splits. Pairwise comparisons were conducted using the Conover test, with Holm-Bonferroni corrections applied to control for family-wise error rates.

### Explainable AI (XAI)

Feature importance was analysed by the calculation of Shapley values using the SHAP package.^18^ Feature mapping to visualise these results occurred by the decomposition of Shapley values onto their constituent atoms.

## Results and discussion

### Polyester design and synthesis

48 polyesters were synthesised in this work and underwent biodegradability testing, and out of these, 25 were classed as biodegradable. These polymers were designed with the aim of yielding a relatively even split of degradable and non-biodegradable polyesters after biodegradability testing, and avoiding any potential bias in the model training set. The library of polyesters was designed to include linear as well as branched monomers, aromatic as well as aliphatic monomers and sugar-based monomers to endow hydrophilicity. PEG400 and diethylene glycol, hydrophilic (poly)ethers, were also used as diols to endow hydrophilicity, with the anticipation that the presence of the (poly)ether backbone may impact the polyester biodegradability (Fig. 1).

It is widely acknowledged that terephthalate-based polyesters do not readily biodegrade.^26^ Therefore, both terephthalic and isophthalic acid were selected in the synthesis of potentially non-degradable polymers. It is also known that aliphatic diacids and diols, up to a certain length, when incorporated into polyesters, can be biodegradable.^27^ Furan-based monomers have received significant attention as terephthalic acid alternatives in polyester synthesis.^28^ Therefore, several furan-containing polyesters were synthesised to assess the differences in biodegradability between these two moieties.

Using the library of polymers synthesised in-house, a fluorescence-based enzymatic biodegradation assay was developed, based on a previously reported method using lipase from porcine pancreas.^29^ The use of this enzyme in polyester biodegradation is well-reported.^30–33^ The fluorescence of fluorescein is pH-dependent due to the cationic, neutral, anionic and dianionic forms that fluorescein can adopt depending on the pH of the solution it is in.^34,35^ The cationic species is most prevalent in acidic conditions, while between pH 4.3 and 6.4, the monoanionic form is most common. The dianion of fluorescein exists at pH values greater than 6.4.^36^

Prior to the development of the fluorescein-based fluorescence assay, another UV-vis absorption-based biodegradation assay was trialled involving the use of phenol red, a pH-responsive dye, as has been previously published by Pirillo *et al.*^37^ However, it was found that the biodegradation products of several of the polymers screened absorbed in a similar region to phenol red, rendering it difficult to determine the true absorbance of the dye, and hence the pH of the solution and the extent of biodegradation.

**Fig. 2:**
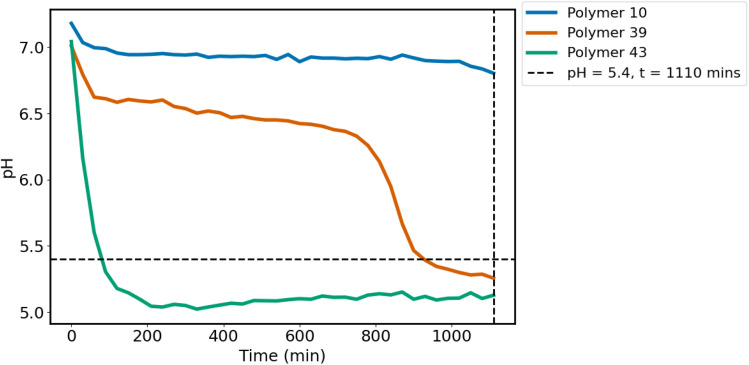
Biodegradation profiles of three different polyesters measured over 18.5 h, showing two biodegradable polyesters and one non-biodegradable polyester. Polymer 10 = poly(PEG400 adipate), polymer 39 = poly(1,3-propylene furandicarboxylate) and polymer 43 = poly(PEG400 azelate).

Exploiting the pH-dependent fluorescence intensity of fluorescein, the biodegradation profile of the polyesters was assessed within 18 hours, significantly faster than can be achieved by alternative literature assays (Fig. 2). For example, Fransen *et al.* reported the use of a clear-zone biodegradation assay taking up to 13 days^14^ while the more standardised OECD 301 tests that look at gas evolution or consumption as an indicator of biodegradation take up to 28 days.^38^ Whilst the clear-zone assay and OECD testing do offer the advantage of whole-organism biodegradation, their long timeframes do not make them attractive preliminary biodegradation screening techniques.

To assess the correlation between the fluorescence-based assay and whole-organism assays, the biodegradability of 20 polyesters, synthesised in-house but previously studied by Fransen *et al.*,^14^ was first investigated (Table 3). Here, it was found that after 18.5 h, the agreement between the two assays was 80%. Using this time as a cutoff point to assess biodegradability, the library of in-house polyesters was then tested. The biodegradation data obtained using this assay were used for ML predictions of biodegradability. Control reactions confirmed that in the absence of the enzyme, pH change was minimal, with a limited examples of a small degree of polymer hydrolysis.

**Table 3:** A confusion matrix of the results of the literature and in-house biodegradability assays for the 20 polymers found in both datasets

		In-house		
		Biodegradable	Non-biodegradable	Sum
Fransen *et al.*	Biodegradable	5	3	8
	Non-biodegradable	1	11	12
	Sum	6	14	20

Whilst pH measurement offers a rapid, accessible approach to biodegradation monitoring, there are limitations to this method. Clearly, this method is most applicable to polyesters and other similar polymers with labile functionalities that would cause a pH change upon degradation. Furthermore, there is a risk of ‘false positive’ results that may be caused by the presence of short oligomeric chains, giving an artificially low pH reading in the absence of any biodegradation taking place.

To mitigate this, pH was monitored from *t* = 0 h (the point at which the enzyme was added to the polymer) every 30 minutes for 18.5 h. The initial pH of most polymers lay between 6 and 7, and this is likely influenced by the short chain length of the polymers and the initial presence of carboxylic acid end groups.

### Benchmarking: featurisation and algorithms

In the development of the predictive model, several model configurations were assessed. The algorithms that were considered were RF and NN, and the featurisation techniques were RDKit fingerprints and descriptors. Additionally, regression modelling was tested to predict biodegradation half-life and pH at 1110 minutes. However, its performance was poor, and it was not pursued further. Details of the regression model benchmarking are provided in the SI. Statistical methods were used to determine the best-performing classification model.

**Fig. 3:**
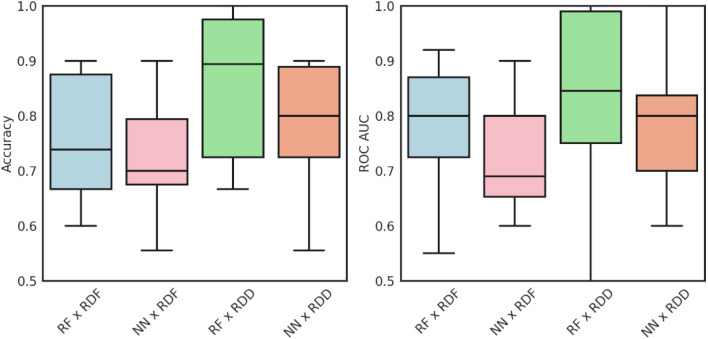
Comparison of the accuracy and ROC AUC of feature representations and algorithms. RF = random forest, NN = neural network, RDF = RDKit fingerprint, RDD = RDKit descriptors.

As shown in Fig. 3, no statistical difference was found between methods in accuracy nor receiver operating characteristic area under the curve (ROC AUC), with Friedman's test *p*-values of 0.070 and 0.195, respectively. As all methods were statistically equivalent in performance, an RF with RDKit fingerprints was chosen as the final model configuration. RDKit fingerprints allow more intuitive, visual explanations of models and facilitate the creation of an interpretable model.

### Final model results

The performance metrics of the final model using the in-house data are as follows. The optimised RF model with RDKit fingerprints achieved an accuracy of 70.7% (standard deviation (SD) = 15.3%), a precision of 0.71 (SD = 0.13), a recall of 0.72 (SD = 0.20), and an F1 score of 0.71 (SD = 0.17). The ROC-AUC score was 0.77 (SD = 0.07), reflecting the model's ability to discriminate between biodegradable and non-biodegradable polymers.

This performance is broadly comparable to that of similar models reported in the literature, despite the smaller size of the training set.^39–41^ However, existing models differ substantially in their applicability to our use case. Many are either trained on datasets that do not focus on polyesters, rely on experimentally derived physicochemical properties such as glass transition temperature or solubility, or predict different biodegradability endpoints derived from distinct assay systems. These factors limit their suitability for virtual screening workflows, where predictions must be made directly from molecular structure without the need for experimental data. In contrast, our model is specifically trained on polyesters and utilises exclusively computationally derived structural descriptors, allowing *in silico* screening of novel polyesters for biodegradability.

Although Fransen *et al.*^14^ recently presented a high-performing model for predicting the biodegradability of polyesters and polycarbonates, with an accuracy of 82% reported, their approach is also unsuitable for direct application to our use case. The trained models have not been published and cannot be independently validated, and their assay system differs from ours in methodology and biodegradability criteria. Furthermore, much of their dataset lacks functional features critical for surfactant behaviour, such as pendant groups, and the polymers often exhibit poor aqueous solubility. As a result, the chemical space of their study only partially overlaps with the class of polyesters relevant for PLF design, further limiting their model's utility for our intended application. In contrast, our newly developed model provides a structure-based classification tool trained exclusively on polyesters and tailored to a rapid biodegradability assay, offering a valuable resource for the identification of application-ready biodegradable polyester candidates.

### Applicability domain

Defining the applicability domain is crucial for determining whether a model's predictions are valid for a given input. The applicability domain helps assess the novelty of input data and guides decisions on whether predictions should be made, while ensuring that confidence estimates remain meaningful. Using the three-step framework suggested by Hanser *et al.*, we define the applicability domain in terms of its validity, reliability and decidability domains.^42^

We employed the bounding-box approach to define the validity domain. This method establishes the limits of the model's training data in feature space and helps to identify novel objects. Specifically, a sample is considered inside the validity domain if it contains only features observed during training and does not omit any feature present across the entire training set. By restricting predictions to data points within the learned space, this approach reduces the risk of extrapolation and unreliable predictions.

A key advantage of the bounding-box method is its practicality for small datasets. Compared to other methods, it offers a less restrictive definition of the validity domain, which is beneficial when data are limited, as it prevents unnecessary exclusion of potentially valid samples.^43^ Additionally, this approach simplifies the handling of binary features in an explainable fashion, by focusing on their presence or absence, rather than with more complex continuous values.

Despite its advantages, the bounding-box method has inherent limitations. A major challenge is the high dimensionality of the data, which leads to significant empty regions within the defined hyperrectangle. This sparsity increases the likelihood of falsely including novel samples that lie outside the model's expertise.^44^ Another limitation is the issue of bit collisions in molecular fingerprinting. Due to the hashing process in fingerprint generation, distinct substructures can map to the same bit, resulting in potential false positives where unseen substructures are incorrectly classified as being within the validity domain. This problem is not unique to the bounding-box approach but is a common limitation across various validity domain definition strategies.

The reliability domain evaluates the proximity of new samples to the training set and ensures that class probability estimates are trustworthy.^43^ The similarity threshold can be adjusted depending on the use case; here, we present an illustrative example. We assessed the information density around each test polymer using Tanimoto similarity,^45^ comparing the RDKit fingerprint of each sample to its five nearest neighbours in the training set. Leave-one-out cross-validation was used to give a more complete picture of the size of the domain, and no samples were found to be outside the validity domain. The 48 samples were divided into three similarity groups: a low-similarity group (<0.8 Tanimoto similarity) with 19 samples, a mid-similarity group (0.8–0.9) with 10 samples, and a high-similarity group (>0.9) with 19 samples (Table S7). Polymers in the high-similarity group tended to feature multiple examples of their constituent diols and diacids within the training set, whereas those in the low-similarity group often contained previously unseen or less-represented diols or diacids. A calibration curve was generated for different levels of similarity, providing an empirical basis for assessing the confidence in a prediction.

**Fig. 4:**
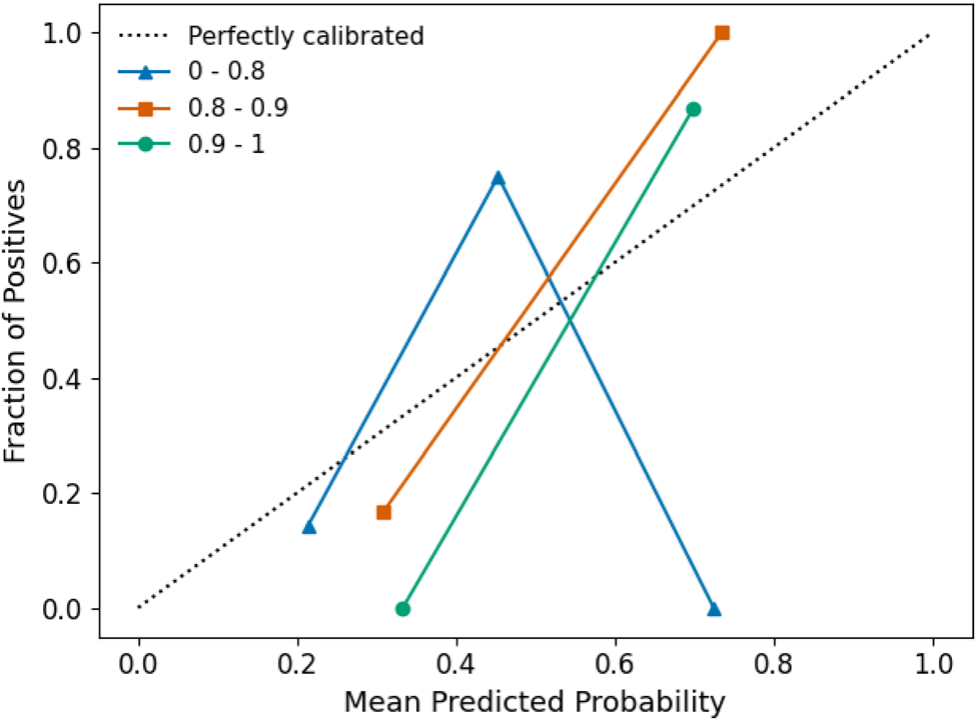
Calibration curves for different levels of Tanimoto similarity of test samples to the training set, where each point represents at least four samples.

Fig. 4 illustrates the calibration curves for each level of structural similarity to the training set, showing how well the model's predicted probabilities align with the observed class frequencies. Our results indicate that the model was poorly calibrated for samples in the low-similarity group (<0.8), making their predicted class probabilities unreliable. Therefore, predictions for samples with similarity below this threshold should be discounted. This problem could be overcome by expanding the size of the training set to further increase model generalisability. In contrast, the mid- and high-similarity groups exhibited better calibration and are considered within the reliability domain.

While a polymer may fall within the validity and reliability domains, it may still lie outside the decidability domain if the model's predicted probability is close to 50%. The decidability domain identifies samples with high uncertainty, typically those near the decision boundary, where the model cannot confidently assign a class label. Since predictions within the reliability domain were shown to be well-calibrated, the model's error rates can be estimated directly from predicted probabilities. To minimise errors, predictions with high uncertainty are rejected, as these correspond to cases where the model is inherently less reliable. Samples with probabilities near the decision boundary are excluded from the decidability domain to improve overall prediction quality. Klingspohn *et al.* showed that built-in class probability estimates are particularly effective at reducing error rates.^46^

The choice of the threshold for the decidability domain involves balancing accuracy and the number of samples for which predictions can be made, which can vary depending on the specific application.

### Prospective prediction

To assess the model's performance under fully prospective conditions and eliminate any possibility of data leakage, seven new polymers were selected and synthesised (details provided in the SI). All seven samples fell within both the validity and reliability domains of the final model. The model correctly classified four of these polymers. On average, correctly predicted samples exhibited slightly higher mean Tanimoto similarity to their five nearest neighbours in the training set, suggesting that higher structural similarity enhances prediction reliability.

In addition, three further polymers were synthesised that lay outside the model's validity domain but showed high structural similarity to the training data. Of these, none was correctly predicted, highlighting the importance of restricting predictions to samples within the defined applicability domain. This result supports the robustness of the domain boundaries in preventing unreliable extrapolations. However, given the limited number of prospective samples, these observations should be interpreted carefully, and additional validation with larger datasets would be valuable to confirm these findings.

### Explaining predictions

To gain insight and understanding into the model's decision-making process, SHAP values were calculated, providing a ranking of the most influential model features. SHAP scores assess the relative contribution of model features to the output, where positive SHAP values indicate that a feature will influence the model to predict the polymer as biodegradable.

**Fig. 5:**
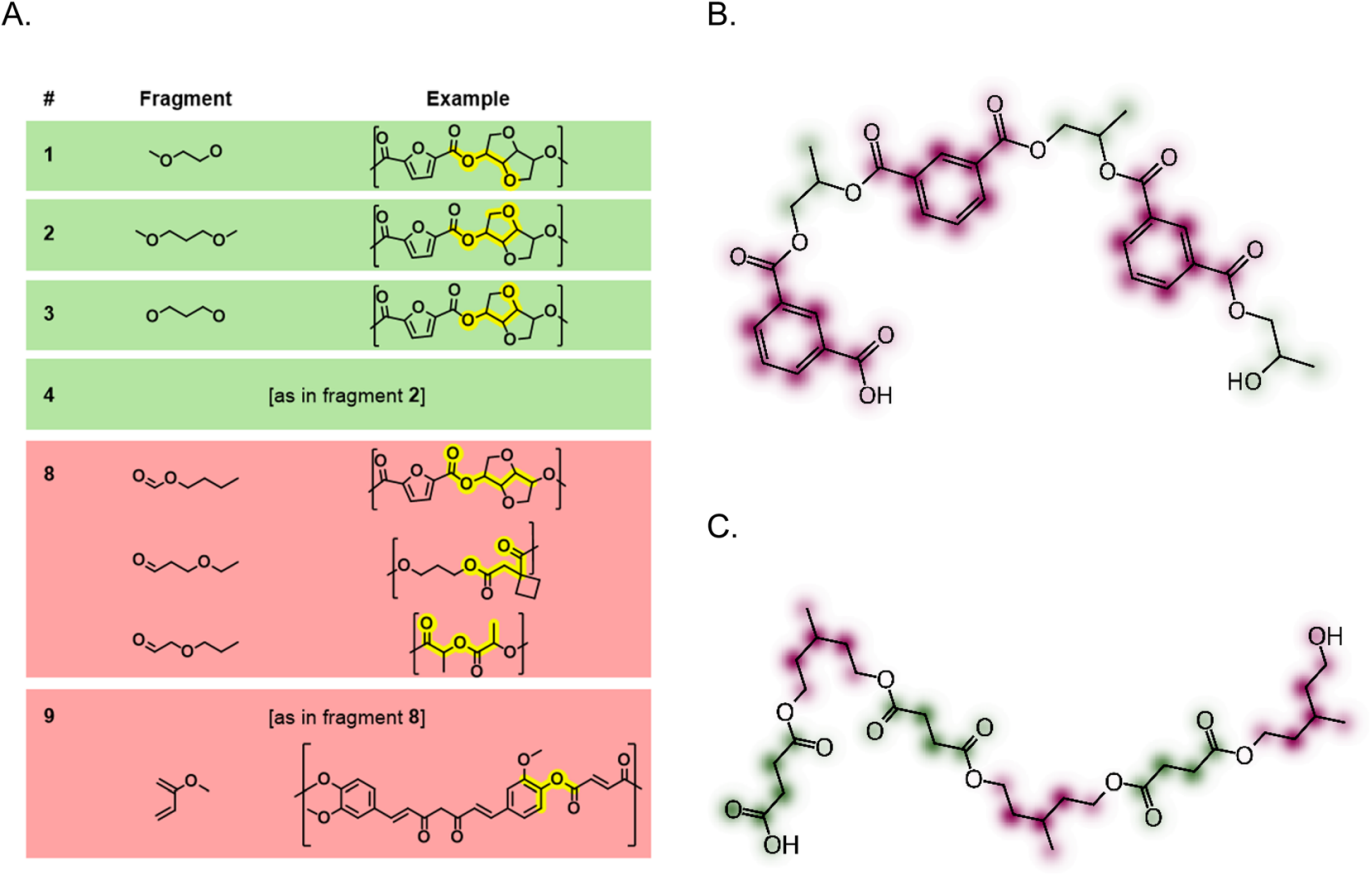
(A) Visualisation of six of the most influential features used in the model, ordered by their mean contribution to the prediction outcome. Features that influence the model to predict the polymer as biodegradable are coloured green, and those that influence it to predict non-biodegradable are in red. Note that the fragments do not have implicit hydrogens, as only heavy atoms are represented in the model. Examples of polymers in the training set are shown, with the fragment highlighted in yellow. (B and C) Feature mapping onto trimer structures demonstrating areas contributing positively (green) or negatively (pink) to biodegradation.

The most influential features used in the model have been identified using SHAP analysis and visualised in Fig. 5, and the top features show repeats of the same substructures. According to the SHAP analysis, fragments 2, 3 and 4, found in glycerol and 1,3-propanediol as well as isosorbide, contribute very positively to biodegradability when present in a polymer. When absent from a polymer, there is an observable decrease in biodegradability. The glycerol-based polymers assessed in this study are water-soluble polymers, and it is possible that this facilitates the interaction of the polymer with the lipase in the biodegradation assay. Whilst hydrophilicity (log *P*) alone cannot explain these trends, it does contribute to the trends observed in the SHAP analysis (Fig. S5 and S6). This was verified by performing SHAP analysis on the RF model built using RDKit descriptors, which found log *P* to be a top feature.

Polymers were found to have a lower biodegradability when fragment 9, an unsaturated moiety found in the curcumin, isophthalate, terephthalate and furan-based polyesters, was present in the polymer structure. Notably, fragment 9 is also part of a larger, conjugated π-system. Polymers such as these have a higher tendency to π–π stack, reducing the likelihood of enzymatic hydrolysis occurring due to the strong interactions between polymer chains. Feature mapping shown in Fig. 5B demonstrates how the isophthalate group hinders biodegradability, despite the presence of groups that appear to have a weak, positive influence on biodegradability.

Fragment 8, present in poly(lactic acid) as well as poly(3-methyl-1,5-pentylene succinate) (Fig. 5C), was detrimental to biodegradability. The nature of this fragment, which includes the methyl group present in poly(lactic acid), may hinder enzyme access to the cleavable ester group. However, this is an example of a bit collision where three moieties are represented by a single fingerprint bit. It is possible that one of these may be more influential towards biodegradability than the others.

As noted previously, bit collisions present a limitation for molecular fingerprinting approaches. Due to conflating the contributions of chemically distinct fragments within the same fingerprint bit, it is difficult to link a specific fragment to a given model outcome unambiguously. To enhance interpretability, alternative non-hashed molecular representations could be explored. SMARTS,^47^ for instance, avoid bit collisions through explicit definition of the substructures to be represented, while learned representations use machine learning to extract meaningful descriptors.^48^

Analysis of SHAP interaction scores revealed there are no significant interactions between features. Of the features used in the model, the top 20 have a cumulative SHAP score representing less than a third of the model, with the top feature having less than a 3% share. This shows the top features cannot be considered in isolation, though they can give insight.

### Transfer learning

**Fig. 6:**
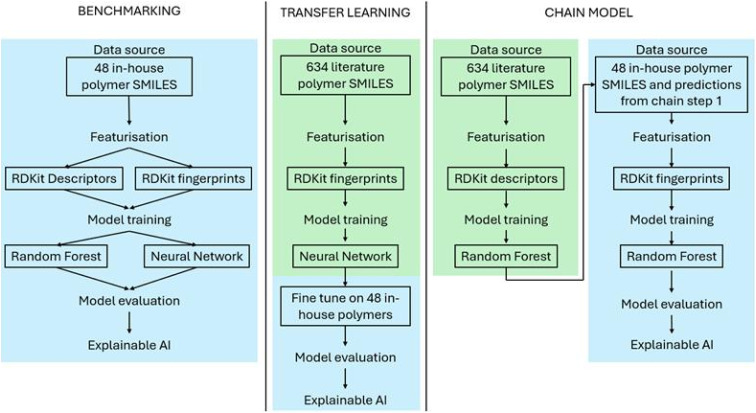
Model architectures for the benchmarking, transfer learning and chained models.

Given the similarity to prior work by Fransen *et al.*, an attempt was made to utilise their literature data to improve the performance of the in-house model.^14^ Both sources focus on polyester biodegradability, though under different experimental conditions. Despite this distinction, the two datasets share overlapping polymer classes, with 20 polymers found in both, making their data a potentially valuable resource for model improvement. Two approaches were tested: transfer learning through fine-tuning of a NN and a chained model, where predictions from the Fransen model were used as an additional feature (Fig. 6). Both approaches are discussed below, and their respective methods can be found in the SI.

Transfer learning has been used in a range of contexts in polymer informatics^49–52^ and was attempted recently in the prediction of polymer biodegradability^41^ using data from small molecule biodegradability, though it did not offer a performance increase. In this work, we used literature data from Fransen *et al.* to pre-train a NN to predict biodegradability as defined in the external study.^14^ Its output layer was then retrained on the in-house dataset to predict in-house biodegradability. A final fine-tuning step involved training the entire network with a low learning rate for one epoch to improve generalisation (Fig. 6). While this approach did not exceed the baseline model's performance (achieving 67% accuracy), it provided valuable insights into the challenges of cross-domain knowledge transfer in polymer biodegradability prediction.

The absence of performance gains from this method is attributed to differences in task and input domains between the datasets. Although both studies focused on polymer biodegradability, the target data predicted enzyme-driven biodegradability, whereas the source data focused on whole-cell biodegradability under different conditions. Furthermore, the datasets occupied different regions of chemical space (Fig. S3 and S4); while both included polyesters, their compositions diverged significantly. The Optimal Transport Dataset Distance^53^ between the source and target datasets, normalised by the geometric mean of the datasets' self-distances, was 49.7. This indicates that the datasets are substantially more dissimilar than the variation observed within each dataset, supporting low expected transferability. Transfer learning typically performs well when differences in task or input domain are modest, but struggles when both aspects differ substantially.^54^ Addressing such challenges may require domain adaptation techniques to align feature spaces or task objectives.

The chained model partially addresses these issues by explicitly aligning tasks and removing out-of-domain features. The approach follows the simple premise of using the output of one model as an input for another, in a sequential manner. In this approach, predictions from a model trained on the Fransen *et al.*^14^ dataset were used as an additional feature for the in-house model (Fig. 6). This method slightly increased the accuracy of the model, achieving 73% accuracy. Feature importance analysis confirmed that the literature prediction was a significant feature, suggesting that knowledge transfer had occurred. However, despite the performance improvement, a reduction in explainability and potential for error propagation make this model less desirable than direct prediction.

## Conclusion

The development of biodegradable polymers is hindered by the slow and resource-intensive nature of biodegradation testing, necessitating more efficient screening methods. In this study, an integrated experimental and computational approach was developed to accelerate the discovery of biodegradable polyesters.

A library of 48 amphiphilic polyesters was synthesised using hydrophilic diols and hydrophobic diacids/diesters to generate surfactant-type polymers. A fluorescence-based enzymatic biodegradation assay was developed, significantly reducing testing time compared to traditional whole-organism assays. The 18-hour assay demonstrated a strong correlation with established methods, demonstrating its reliability for preliminary screening.

The biodegradation data obtained from this assay were subsequently utilised to train a machine-learning model. After benchmarking several model configurations, an RF classifier using RDKit fingerprints was selected due to its strong predictive performance while maintaining interpretability. The model achieved an accuracy of 71%, comparable to existing literature models while being tailored to a domain of functional polyesters. By defining the applicability domain in terms of validity, reliability, and decidability, the model ensures robust and meaningful predictions. The use of RDKit fingerprints facilitated interpretability, with SHAP analysis providing insight into key molecular features influencing biodegradability. Furthermore, as the model requires only repeat unit structures as input, it enables *in silico* screening of new polymers before synthesis, reducing experimental workload in the early stages of material development.

To explore the potential for integrating literature data, transfer learning and a chained modelling approach were investigated using data from Fransen *et al.*^14^ Despite their theoretical potential, neither method resulted in improved predictive performance. The lack of improvement is attributed to differences in task objectives and chemical space between datasets, limiting knowledge transfer. While the chained model showed some integration of external knowledge, this did not increase model accuracy, suggesting that additional domain adaptation techniques may be necessary for effective transfer. This could involve approaches such as feature transformations to map both datasets into a shared latent space or adversarial training to learn a domain-invariant representation of the polymer structures.^55^

Future work should focus on expanding the dataset to improve model generalisation as well as including polymer physical properties in the training data to account for differences in molar mass, for example. Extending the modelling approach to include regression or multi-class classification frameworks would allow for the development of predictions for polymers with varying degradation rates or half-lives. Additionally, the utilisation of domain adaptation strategies may enable better integration of larger datasets, enhancing the model's performance and generalisation to a broader range of functional polyesters. By combining rapid experimental methods with machine learning, this study establishes a foundation for more efficient and interpretable biodegradability predictions, supporting the design of sustainable polymeric materials.

## Author contributions

PLJ: conceptualization, data curation, funding, investigation, methodology, writing – original draft, review and editing; MIP: conceptualization, data curation, investigation, methodology, software, visualization, writing – original draft, review and editing; DJK: methodology, writing – review and editing; VT: conceptualization, funding acquisition, supervision, writing – review and editing; SMH: conceptualization, funding acquisition, resources, supervision, writing – review and editing; JDH: conceptualization, funding acquisition, resources, supervision, writing – review and editing.

## Conflicts of interest

There are no conflicts to declare.

## Supplementary Material

SC-OLF-D5SC05380C-s001

## Data Availability

The dataset, model, and code to reproduce the training process are available open-source in alignment with the FAIR (findable, accessible, interoperable, and reproducible) data principles^56^ on Github at https://github.com/maddyparker01/MLPolyesterBiodegradability and archived on Zenodo at https://doi.org/10.5281/zenodo.17545516.^57,58^ Supplementary information (SI): additional details on the synthesis, assay, machine learning models and NMR spectra. See DOI: https://doi.org/10.1039/d5sc05380c.
