# Supplementary material for: Explainable random forest predictions of polyester biodegradability using high-throughput biodegradation data
Source: Chem Sci. 2025 Nov 18;17(2):944–55. doi: 10.1039/d5sc05380c (PMC12645284; doi:10.1039/d5sc05380c)
Supplement: SC-017-D5SC05380C-s001 [file SC-017-D5SC05380C-s001.pdf]

Supplementary Information (SI) for Chemical Science.
This journal is © The Royal Society of Chemistry 2025

Supplementary Information

Materials

All reagents were used as received without further purification unless otherwise stated.

Table S1. Reagent suppliers and purities

Reagent	Supplier	Purity
K_2CO_3	Sigma Aldrich	99%
$Sn(Oct)_2$	Sigma	92.5-100%
**Diacids/diesters**		
Dimethyl furan-2,5-dicarboxylate	Apollo Scientifit	-
Dimethyl succinate	Abcr	98%
Dimethyl suberate	Thermo Scientific	99%
Dimethyl azelate	Synthesised in-house	-
Azeleic acid	Alfa Aesar	96%
Dimethyl adipate	Sigma Aldrich	99%
Dimethyl fumarate	Synthesised in-house	-
Fumaric acid	Alfa Aesar	99%
Cyclobutane-1,1-dicarboxylic acid	abcr	98%
Dimethyl isophthalate	Aldrich	99%
Dimethyl terephthalate	Aldrich	≥99%
Dimethyl sebacate	Thermo Scientific	97%
**Alcohols**		
Isosorbide	Acros Organics	98%
D-Sorbitol	Acros Organics	97%
Xylitol	Sigma Aldrich	
Curcumin	abcr	95%
Glycerol	Sigma-Aldrich	>99%
1,3-propanediol	Aldrich	98%
PEG400	Sigma Aldrich	-
1,2-Propanediol	Sigma Aldrich	>99.5%
1,4-Cyclohexanedimethanol (cis + trans)	Thermo Scientific	99%
3-Methyl-1,5-pentanediol	Sigma Aldrich	≥98%
1,10-Decanediol	Lancaster Synthesis	97%
1,6-Hexanediol	Aldrich	97%
2-Butene-1,4-diol	Aldrich	95%
2,2-Dimethyl-1,3-propanediol	Alfa Aesar	99%
Diethylene glycol	Aldrich	99%
Diglycerol	TCI	-
**Cyclic monomers**		
Lactide	Aldrich	99%
Glycolide	Sigma-Aldrich	>99%
Caprolactone	Thermo Fisher	99%
δ-Valerolactone	Sigma Aldrich	Technical grade
DCM	Fisher Scientific	Reagent grade
**Biodegradation assay**		
Fluorescein	Aldrich	95%
Na_2HPO_4	Fluka	98-100%
NaH_2PO_4	Sigma Aldrich	>99%

Lipase from porcine pancreas, Type II	Sigma	-
DMSO	Fisher Scientific	Reagent grade

Dimethyl fumarate synthesis

A dimethyl fumarate synthesis from literature was followed.[1] Briefly, fumaric acid (10.00 g, 86.15 mmol) was dissolved in methanol (60 mL) with H_2SO_4 (0.5 mL) and heated to 75 °C. The reaction mixture was refluxed for 24 h. The reaction mixture was concentrated *in vacuo* and extracted with diethyl ether (3 × 50 mL) and finally with $NaHCO_3$ (50 mL). The product was dried over magnesium sulfate and the solvent removed *in vacuo*. ^{1}H NMR (400 MHz, $CDCl_3$): δ 6.87 (s, 2H), 3.81 (s, 6H).

Dimethyl azelate synthesis

A dimethyl fumarate synthesis from literature was followed.[1] Briefly, azelaic acid (10.00 g, 53.13 mmol) was dissolved in methanol (60 mL) with H_2SO_4 (0.5 mL) and heated to 75 °C. The reaction mixture was refluxed for 24 h. The reaction mixture was concentrated *in vacuo* and extracted with diethyl ether (3 × 50 mL) and finally with $NaHCO_3$ (50 mL). The product was dried over magnesium sulfate and the solvent removed *in vacuo*. ^{1}H NMR (400 MHz, $CDCl_3$): δ 3.63 (s, 6 H), 2.26 (t, 3H), 4.03 (m, 4H), 1.28 (m, 6H).

Polymer synthesis and characterisation

Polymers were characterised by gel permeation chromatography (GPC) to determine the molar mass, and by nuclear magnetic resonance (NMR) spectroscopy. GPC in either THF, DMF or water was performed according to the solubility of the polymers. All samples were filtered through a 0.45 μm teflon filter prior to analysis. Polymer molar masses and GPC eluent used have been summarised in Tables S4 and S9.

THF: Samples were prepared as a 3 mg/mL solution in THF (HPLC grade, Fisher Scientific). Analysis was performed using a THF (HPLC grade) eluent at 40 °C with two Mixed-D and two Mixed-E columns in series with a flow rate of 1 mL/min. Instrument was calibrated using PMMA standards ranging from 540 to 1.02 × 10^6 g/mol.

DMF: Samples were prepared as a 10 mg/mL solution in DMF (HPLC grade, Fisher Scientific). Analysis was performed using a DMF (HPLC grade) eluent at 80 °C with one mixed-D and one mixed-E column in series with a flow rate of 1 mL/min. Instrument was calibrated using PMMA standards ranging from 540 to 1.02 × 10^6 g/mol.

Water: Samples were prepared as a 3 mg/mL solution in 0.02% sodium azide solution (HPLC grade water, Fisher Scientific). Analysis was performed using a 0.02% sodium azide solution eluent at 40 °C with a mixed-M and a mixed-H column in series with a flow rate of 1 mL/min. Instrument was calibrated using PEG standards ranging from 120 to 1.4 × 10^6 g/mol.

Selection of Polymers for Prospective Prediction

Candidate polymers were selected and screened to ensure they fell within the model's applicability domain. Polymers meeting these criteria were synthesised, and those for which synthesis was successful are reported.

Table S2. Synthetic details for the preparation of polyesters via polycondensation.

Polymer Number	Diol	Diol (mmol)	Diacid/ester	Diester/acid (mmol)	K_2CO_3 (mg)	T (°C)
**1**	Isosorbide	3.00	Dimethyl furan-2,5-dicarboxylate	3.00	46	120
**2**	Sorbitol	25.00	Dimethyl succinate	25.00	410	120

**3**	Sorbitol	2.47	Dimethyl suberate	2.47	43	120
**4**	Sorbitol	2.31	Dimethyl azelate	2.31	46	120
**5**	Xylitol	3.00	Dimethyl adipate	3.00	48	100
**6**	Curcumin	6.57	Dimethyl fumarate	6.57	159	80
**7**	Isosorbide	6.57	Dimethyl fumarate	6.57	86	120
**8**	Glycerol	2.47	Dimethyl suberate	2.47	37	100
**9**	1,3-propanediol	6.57	Cyclobutane-1,1-dicarboxylic acid	6.57	63	120
**10**	PEG400	10.26	Dimethyl adipate	10.26	294	70
**11**	PEG400	2.72	Dimethyl furan-2,5-dicarboxylate	2.72	78	120
**12***	1,2-propanediol	25	Dimethyl isophthalate	25	338	120
**13***	1,4-cyclohexanedimethanol	6.57	Dimethyl terephthalate	6.57	93	120
**14***	3-methyl-1,5-pentanediol	25	Dimethyl terephthalate	25	390	120-150
**15***	1,10-decanediol	25	Dimethyl terephthalate	25	460	120-150
**16***	1,6-hexanediol	6.57	Dimethyl furan-2,5-dicarboxylate	6.57	99	120
**17***	2-Butene-1,4-diol	8.46	Dimethyl succinate	8.46	137	120
**18***	3-methyl-1,5-pentanediol	25	Dimethyl succinate	25	280	120
**19***	1,10-decanediol	6.57	Dimethyl succinate	6.57	105	80
**20***	2,2-dimethyl-1,3-propanediol	25	Dimethyl adipate	25	347	120
**21***	1,6-hexanediol	25	Dimethyl adipate	25	365	95
**22***	Diethylene glycol	25	Dimethyl adipate	25	350	90
**23***	1,4-cyclohexanedimethanol	8.46	Dimethyl sebacate	8.46	147	120
**24***	1,10-decanediol	25	Dimethyl sebacate	25	436	120
**25***	1,6-hexanediol	25	Dimethyl sebacate	25	436	95
**26***	Diethylene glycol	25	Dimethyl sebacate	25	315	90
**27**	Glycerol	2.72	Dimethyl furan-2,5-dicarboxylate	2.72	38	120
**28**	Glycerol	5.42	Dimethyl succinate	5.42	65	100
**29**	Glycerol	10.26	Dimethyl adipate	10.26	136	100
**30**	Glycerol	2.31	Dimethyl azelate	2.31	35	120
**31**	Glycerol	10.26	Dimethyl sebacate	10.26	165	100
**32**	Diglycerol	10.26	Dimethyl succinate	10.26	110	90
**33**	Diglycerol	10.26	Dimethyl adipate	10.26	139	100
**34**	Diglycerol	2.31	Dimethyl azelate	2.31	38	120
**35**	Diglycerol	2.72	Dimethyl furan-2,5-dicarboxylate	2.72	48	120
**36**	Diglycerol	3.00	Dimethyl azelate	3.00	58	80
**37**	Diglycerol	10.26	Dimethyl sebacate	10.26	203	100
**38**	Diglycerol	2.47	Dimethyl suberate	2.47	39	100
**39**	1,3-propanediol	3.00	Dimethyl furan-2,5-dicarboxylate	3.00	39.0	120
**40***	1,3-propanediol	8.46	Dimethyl succinate	8.46	82.2	120
**41**	PEG400	10.26	Dimethyl succinate	10.26	280	70
**42**	PEG400	2.47	Dimethyl suberate	2.47	74	70
**43**	PEG400	2.31	Dimethyl azelate	2.31	71	70
**44**	PEG400	10.26	Dimethyl sebacate	10.26	203	100

*Polymers marked with an asterisk also appear in the literature dataset.[2]

Table S3. Synthetic details for the preparation of polyesters via ROP

**Polymer Number**	**Benzyl alcohol (mmol)**	**Monomer(s)**	**Monomer 1 (mmol)**	**Monomer 2 (mmol)**	**Sn(Oct)$_2$ (mmol)**	**Temperature (°C)**
**45***	0.3504	Caprolactone	17.52	-	0.01752	130
**46***	0.3993	Valerolactone	19.97	-	0.01947	130

**47***	0.2775	Lactide	13.88	-	0.01388	130
**48***	0.3466	Lactide / glycolide	8.62	8.62	0.01724	130

*Polymers marked with an asterisk also appear in the literature dataset.[2]

Table S4. Polymer molar masses.

Polymer Number	Eluent	M_n (g/mol)	M_w (g/mol)	$Đ$
**1**	DMF	610	870	1.4
**2**	-	n.d.	n.d.	n.d.
**3**	Water	510	4745	9.3
**4**	Water	680	3472	5.1
**5**	DMF	4160	7410	1.8
**6**	THF	1030	1270	1.2
**7**	DMF	1780	2895	1.6
**8**	DMF	1725	2225	1.3
**9**	THF	470	580	1.2
**10**	DMF	2065	3305	1.6
**11**	DMF	3045	4450	1.5
**12**	THF	2080	3545	1.7
**13**	-	n.d.	n.d.	n.d.
**14**	THF	2795	4480	1.6
**15**	-	n.d.	n.d.	n.d.
**16**	-	n.d.	n.d.	n.d.
**17**	THF	1345	3530	2.5
**18**	THF	3120	6385	2.0
**19**	THF	915	2900	3.2
**20**	THF	1030	1620	1.6
**21**	THF	6135	11430	1.9
**22**	THF	5835	10860	1.8
**23**	THF	910	1880	2.1
**24**	DMF	871	1070	1.2
**25**	THF	7470	13680	1.8
**26**	THF	2510	6260	2.5
**27**	DMF	1480	2115	1.4
**28**	DMF	3130	5795	1.9
**29**	DMF	2465	3485	1.4
**30**	DMF	2490	3820	1.5
**31**	DMF	2410	3291	1.4
**32**	DMF	3310	4595	1.4
**33**	DMF	3945	7045	1.8
**34**	DMF	4405	9690	2.2
**35**	DMF	2210	3290	1.5
**36**	DMF	1350	1665	1.2
**37**	DMF	3950	6330	1.6
**38**	DMF	3295	4355	1.3
**39**	-	n.d.	n.d.	n.d.
**40**	THF	1450	2785	1.9
**41**	DMF	1950	3085	1.6
**42**	DMF	1500	2105	1.4

**43**	DMF	1340	1785	1.3
**44**	DMF	1970	3100	1.6
**45**	THF	9120	12900	1.4
**46**	THF	6940	13360	1.4
**47**	THF	12830	15300	1.2
**48**	THF	4990	14690	2.9

* Several samples were not soluble in GPC eluents; therefore, it was not possible to determine their molar mass. These samples are marked with n.d.

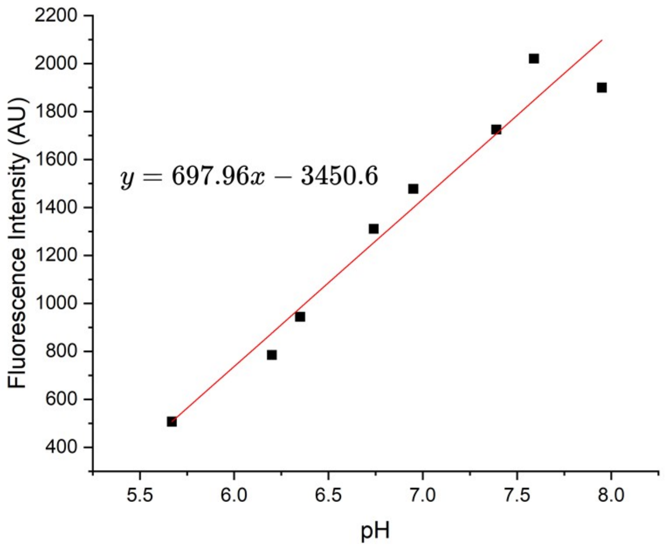

Figure S1. Calibration of pH and fluorescence intensity of fluorescein (R^2 = 0.9577). The lowest pH value that can be accurately determined using this calibration curve is 4.94, where fluorescence intensity = 0.

Table S5. pH values at t = 0 h for biodegradation assay. Values are an average of the repeat measurements alongside a standard deviation.

Polymer Number	pH	Standard Deviation
**1**	6.57	0.05
**2**	7.16	0.17
**3**	6.95	0.03
**4**	6.95	0.46
**5**	6.88	0.06
**6**	7.11	0.07
**7**	6.68	0.09
**8**	6.65	0.14
**9**	6.93	0.16
**10**	7.06	0.11
**11**	6.48	0.13

**12**	7.29	0.24
**13**	7.19	0.23
**14**	7.35	0.28
**15**	7.37	0.19
**16**	6.93	0.24
**17**	6.66	0.25
**18**	6.86	0.34
**19**	7.09	0.20
**20**	7.16	0.20
**21**	7.33	0.22
**22**	6.56	0.85
**23**	7.04	0.18
**24**	7.34	0.21
**25**	7.47	0.31
**26**	7.22	0.34
**27**	6.57	0.22
**28**	6.70	0.05
**29**	6.87	0.20
**30**	5.82	0.26
**31**	5.86	0.17
**32**	6.99	0.16
**33**	6.94	0.05
**34**	6.58	0.28
**35**	6.46	0.22
**36**	6.87	0.21
**37**	6.76	0.11
**38**	6.65	0.09
**39**	6.86	0.13
**40**	7.06	0.19
**41**	6.96	0.15
**42**	6.67	0.08
**43**	7.57	2.03
**44**	6.74	0.26
**45**	7.04	0.38
**46**	6.89	0.23
**47**	6.90	0.24
**48**	7.14	0.28

Table S6. pH values at t = 0 h and t = 18.5 h for control reactions (without enzyme) of the biodegradation assay. Values are an average of the repeat measurements alongside a standard deviation.

Polymer Number	t = 0 h pH	Standard Deviation	t = 18.5 h pH	Standard Deviation
**1**	6.67	0.10	5.94	0.07
**2**	7.11	0.23	6.88	0.10
**3**	6.90	0.07	6.76	0.07
**4**	6.75	0.28	6.56	0.29
**5**	6.96	0.13	6.73	0.15
**6**	6.70	0.66	6.57	0.30
**7**	6.63	0.13	5.49	0.11
**8**	6.88	0.20	6.76	0.31
**9**	6.86	0.11	6.67	0.21
**10**	7.08	0.25	6.90	0.11
**11**	6.51	0.06	6.16	0.13

**12**	7.22	0.09	6.90	0.28
**13**	7.21	0.16	6.96	0.23
**14**	7.23	0.27	7.20	0.21
**15**	7.30	0.13	7.17	0.15
**16**	7.15	0.14	6.95	0.15
**17**	6.95	0.47	6.88	0.27
**18**	7.27	0.12	7.22	0.16
**19**	7.10	0.15	6.87	0.19
**20**	7.34	0.10	7.26	0.19
**21**	7.25	0.12	7.13	0.09
**22**	7.37	0.04	7.26	0.10
**23**	7.11	0.21	6.75	0.37
**24**	7.27	0.05	7.07	0.14
**25**	7.38	0.19	7.13	0.04
**26**	7.29	0.22	7.17	0.23
**27**	6.46	0.21	5.78	0.47
**28**	6.87	0.01	6.56	0.20
**29**	7.03	0.12	6.75	0.07
**30**	6.85	0.12	6.71	0.20
**31**	6.88	0.36	6.90	0.14
**32**	7.11	0.12	6.80	0.08
**33**	6.94	0.07	6.71	0.09
**34**	6.83	0.04	6.74	0.08
**35**	6.51	0.05	6.07	0.30
**36**	7.06	0.17	7.04	0.21
**37**	7.07	0.06	6.94	0.02
**38**	6.87	0.03	6.76	0.10
**39**	6.77	0.16	6.37	0.15
**40**	7.28	0.19	7.04	0.05
**41**	7.09	0.20	6.91	0.07
**42**	6.79	0.16	6.62	0.17
**43**	6.70	0.06	6.64	0.21
**44**	6.89	0.10	6.83	0.05
**45**	7.18	0.07	6.95	0.14
**46**	7.18	0.12	6.89	0.17
**47**	7.21	0.10	6.76	0.16
**48**	7.17	0.11	6.69	0.13

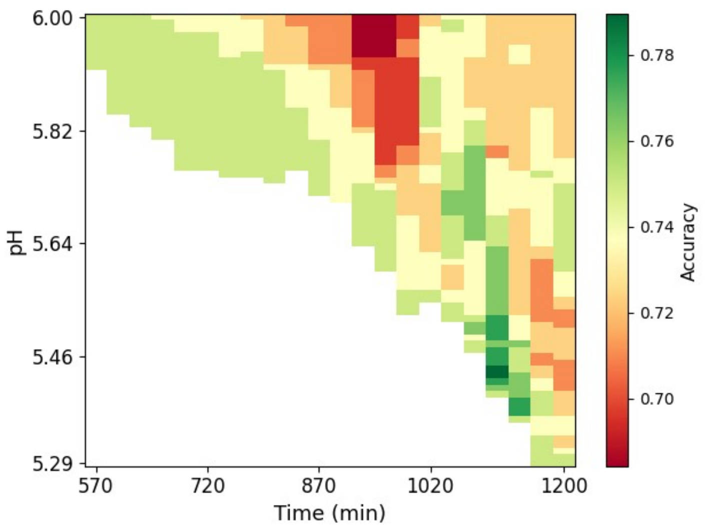

Figure S2. Accuracy of the in-house assay for the 20 polymers that appeared in both datasets, relative to the Fransen assay.

Regression Models: Hill Model

A hill model[3] was fitted to the pH data for each of the 157 samples over 0 – 1200 minutes.

$$y = a - \frac{bt^c}{d^c + t^c} \qquad \textbf{Equation 1}$$

Where a is the starting pH, b is the total pH drop, c is the Hill coefficient, which reflects the shape of the curve (exponential or sigmoidal), d is the half-life of biodegradation, and t is time.

Polymers that showed no pH drop were removed from the dataset as a curve couldn't be fitted, and those with negative fit parameters were filtered out. Most of the remaining samples had an R^2 greater than 0.98, and those with an R^2 less than 0.8 were removed, leaving 73 samples covering 33 polymers.

Both c, the hill coefficient, and d, the half-life of biodegradation, were used as target variables.

Training

Featurisation and training followed the main text, differing only in the use of group fold cross-validation as the data splitting strategy, and R^2 as the performance metric.

Regression Models: pH at Time Threshold

pH at 1110 minutes was used as the target variable, with the above training method.

Results

None of the three models achieved a mean test R^2 value greater than 0.2, and further modelling was not performed.

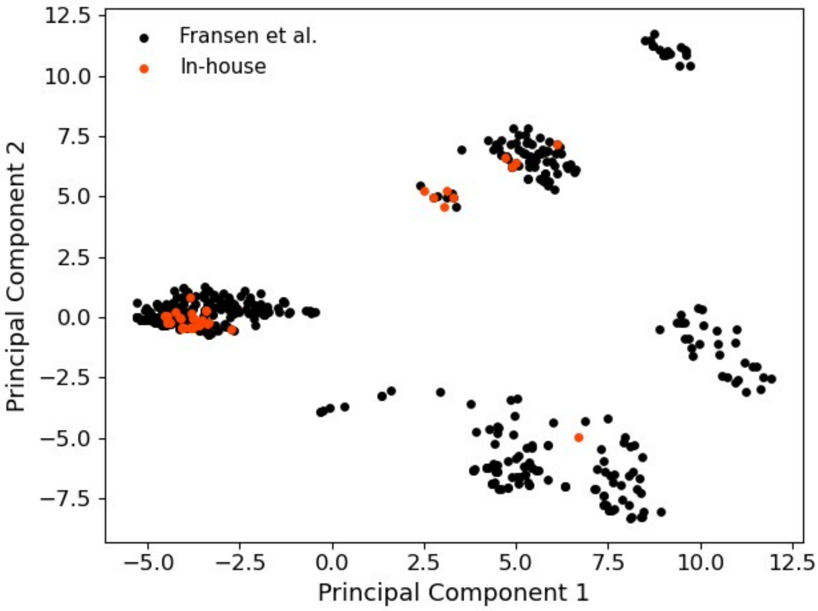

Figure S3. Principal component analysis of the literature and in-house polymers, using RDKit fingerprints as described in the main text. 21.4 and 13.1 % of the variance of the data is explained by components 1 and 2, respectively.

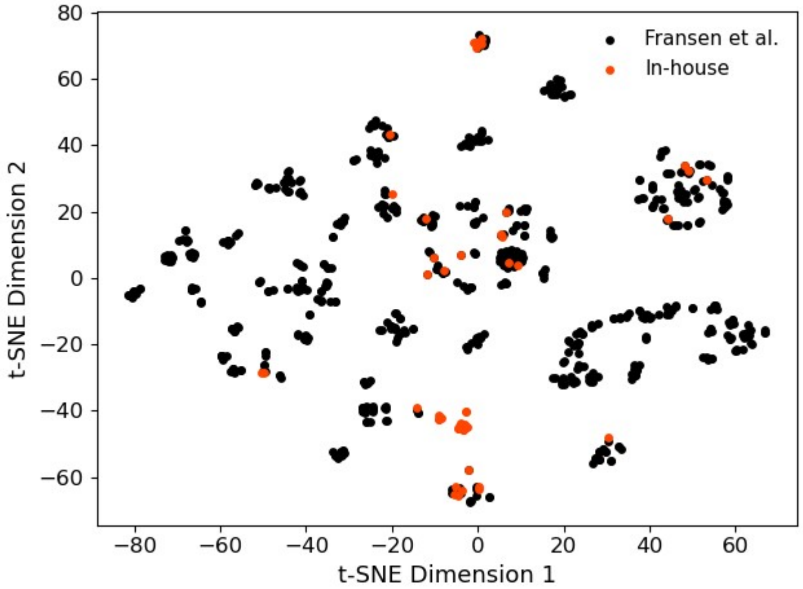

Figure S4. T-SNE of the literature and in-house polymers, using RDKit fingerprints as described in the main text.

Table S7. Similarity of each polymer to its five nearest neighbours in the dataset.

Polymer Number	Similarity Group
**1**	Low

**2**	Medium
**3**	Medium
**4**	Medium
**5**	Medium
**6**	Low
**7**	Low
**8**	High
**9**	Low
**10**	High
**11**	Low
**12**	Low
**13**	Low
**14**	Low
**15**	Low
**16**	Low
**17**	Low
**18**	Low
**19**	Low
**20**	Low
**21**	High
**22**	High
**23**	Low
**24**	High
**25**	High
**26**	High
**27**	Low
**28**	Low
**29**	Medium
**30**	Medium
**31**	High
**32**	High
**33**	High
**34**	Medium
**35**	High
**36**	High
**37**	Medium
**38**	High
**39**	High
**40**	High
**41**	Medium
**42**	High
**43**	Medium
**44**	Low
**45**	Low
**46**	High
**47**	High
**48**	High

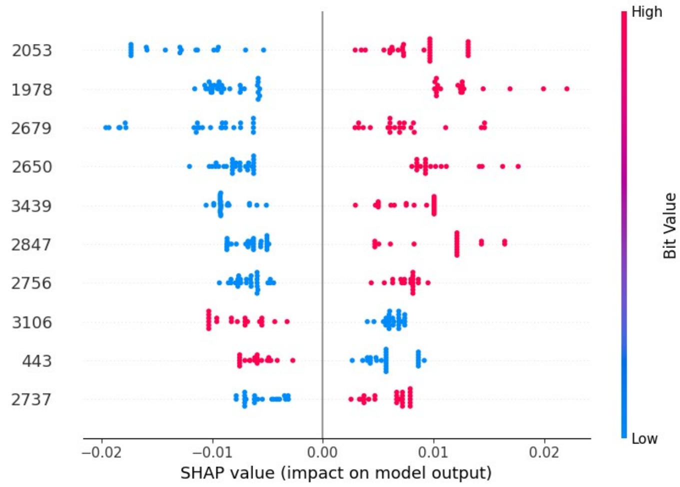

Figure S5. SHAP scores for the 10 most influential features in the RF model using RDKit fingerprints as descriptors.

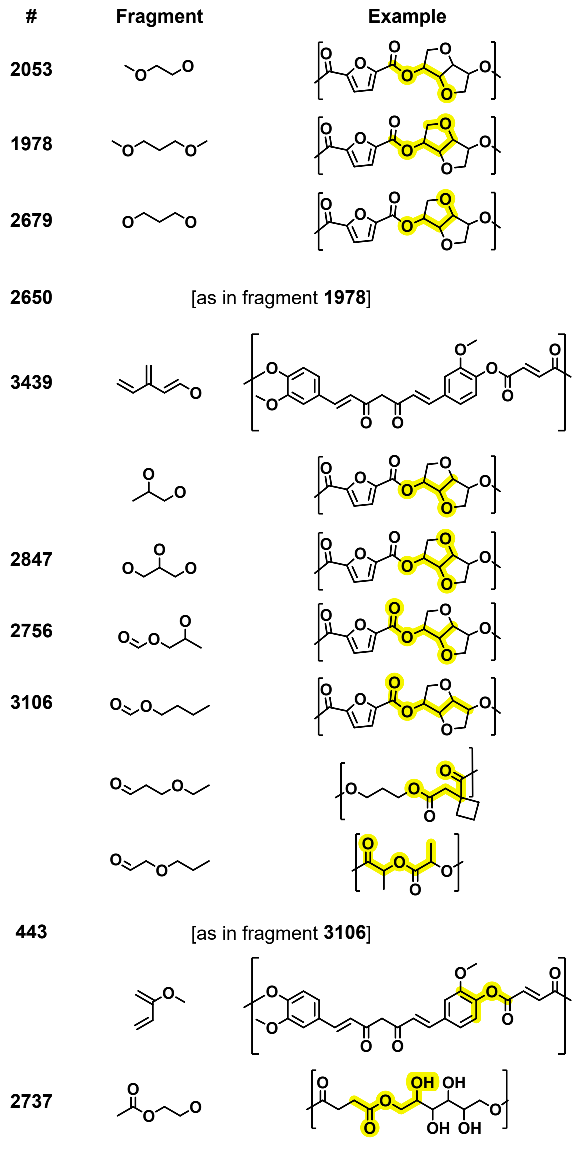

Figure S6. Visualisation of the 10 most influential features with example polymers from the training dataset.

Transfer learning data:

The dataset from Fransen et al. consisted of 658 polymers – polyesters and polycarbonates.[2] Duplicates and conflicting entries were removed, resulting in 634 polymers remaining, of which 327 were biodegradable. Each polymer had the SMILES of its diacid/diol, its composition ratio, and the polymer's biodegradability classification. The dataset also included information on polymer molecular weights and their physical states for some of the samples. However, as found in the original study, this information provides no increase in accuracy, so was removed from the dataset.

The in-house dataset was as in the main text, and 20 polymers appeared in both datasets.

For both datasets, the polymer repeat unit SMILES was 'polymerised' to produce a trimer which acts as the structure representation in all models in this work.

Transfer learning method:

Data: Data scaling was fitted with respect to the literature source data and was used to transform the target data.

Base model structure: Each network formed a fully connected pyramid in which the number of hidden layers was 2. The number of neurons in the first hidden layer was varied {32-128} and was reduced by half for the second hidden layer. Neurons in both hidden layers were activated by ReLU, and a sigmoid activation function was configured on the output layer. A binary cross-entropy loss function was used with the Adam optimiser, and the model was trained for {10-50, steps of 10} epochs.

Transfer learning: The base model weights were frozen, and the last layer was retrained on the assay dataset for 10 epochs. Base model weights were then unfrozen, and fine-tuning of the whole model took place with a learning rate of 1e-5 for 10 epochs to refine the weight parameters. Stratified five-fold cross-validation was used, and accuracy was evaluated on the out-of-fold data.

Analysis: Optimal Transport Dataset Distance was calculated to evaluate domain similarity between source and target datasets, using the associated Python package.[4-6]

Model Chaining (Overview)

The model architecture in this work consists of a sequential chain structure, where the output of an initial model trained on the literature dataset is used as an additional input feature for a subsequent model trained on the in-house dataset. This approach aims to leverage information from the larger literature dataset to improve predictive performance on the smaller in-house dataset.

Model Chain Step 1 - Literature Assay Biodegradability Prediction

Featurisation

For each polymer in the literature dataset, RDKit molecular descriptors were calculated. To ensure that the in-house polymers fell within the domain of applicability of this model, descriptors were filtered by

range. Descriptors were removed if any value in the in-house dataset fell outside the range observed in the literature dataset. This resulted in the removal of 51 features.

A variance threshold was applied to remove descriptors with zero variance, thereby eliminating features that do not contribute to model performance.

Training

To maintain comparability to the published model, the same data splitting technique was used to split the polymers based on their chemistries into groups for cross validation. The exact splits were not provided, but the code to replicate them was. This resulted in six groups where the proportion of ten different functional groups was even across all.

An RF classifier was fitted, following the same procedure as described in the main text but using this 6-fold CV.

Model Chain Step 2 - In-house Assay Biodegradability Prediction

Featurisation

RDKit fingerprints were generated from the SMILES, using the same method as in the main text.

The predicted probability of biodegradability from the literature-based model was used as an additional feature for each polymer in this second model. This probability provided a continuous measure rather than a binary output, allowing the model to gauge confidence in the prediction.

Training

The RF training process mirrored the main text.

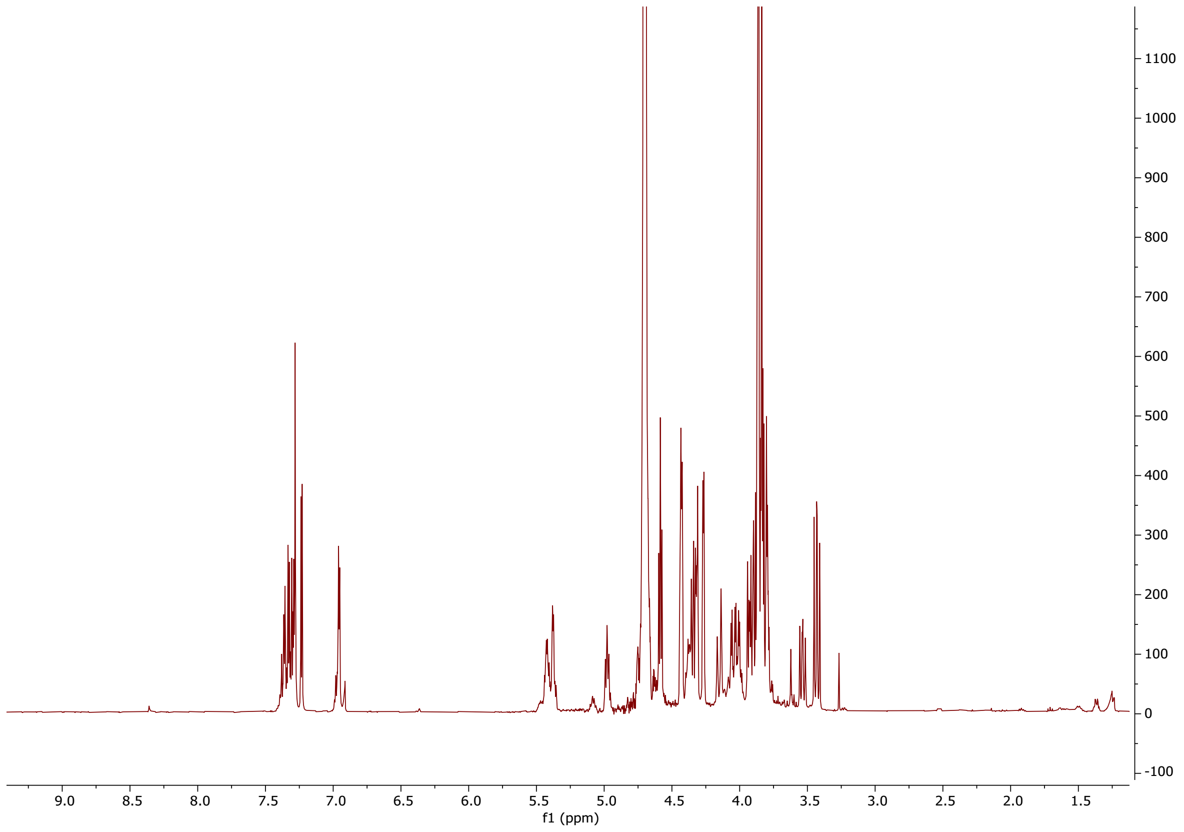

Figure S7. ^1H NMR spectrum of Polymer 1(400 MHz, D_2O).

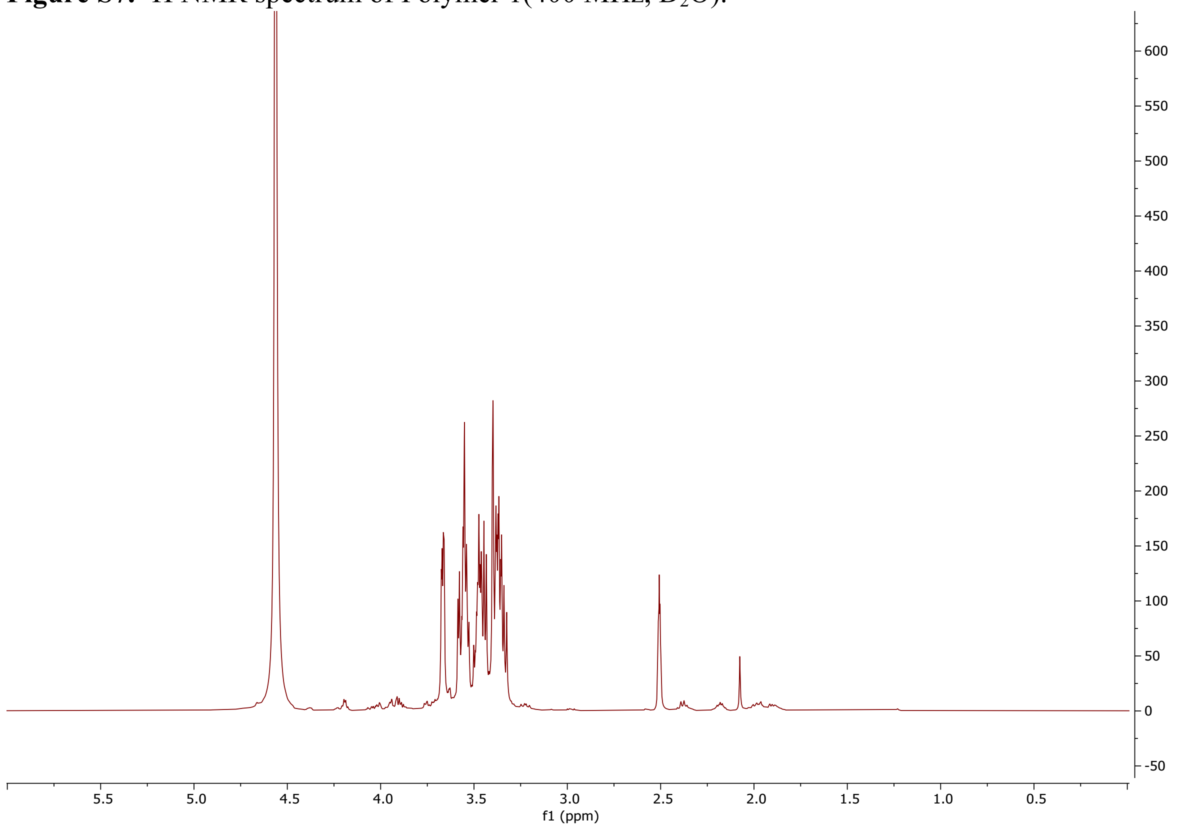

Figure S8. ^1H NMR spectrum of Polymer 2 (400 MHz, DMSO-d_6).

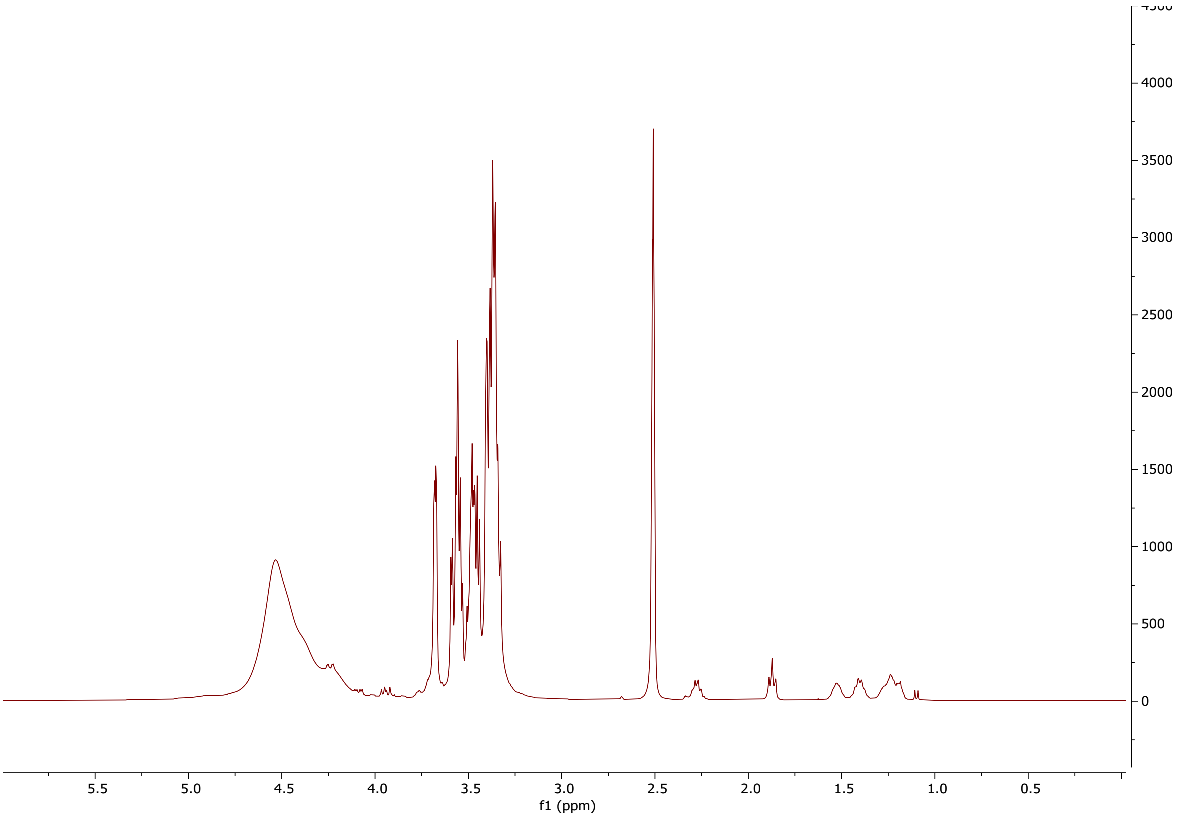

Figure S9. ^{1}H NMR spectrum of Polymer 3 (400 MHz, DMSO-d_6).

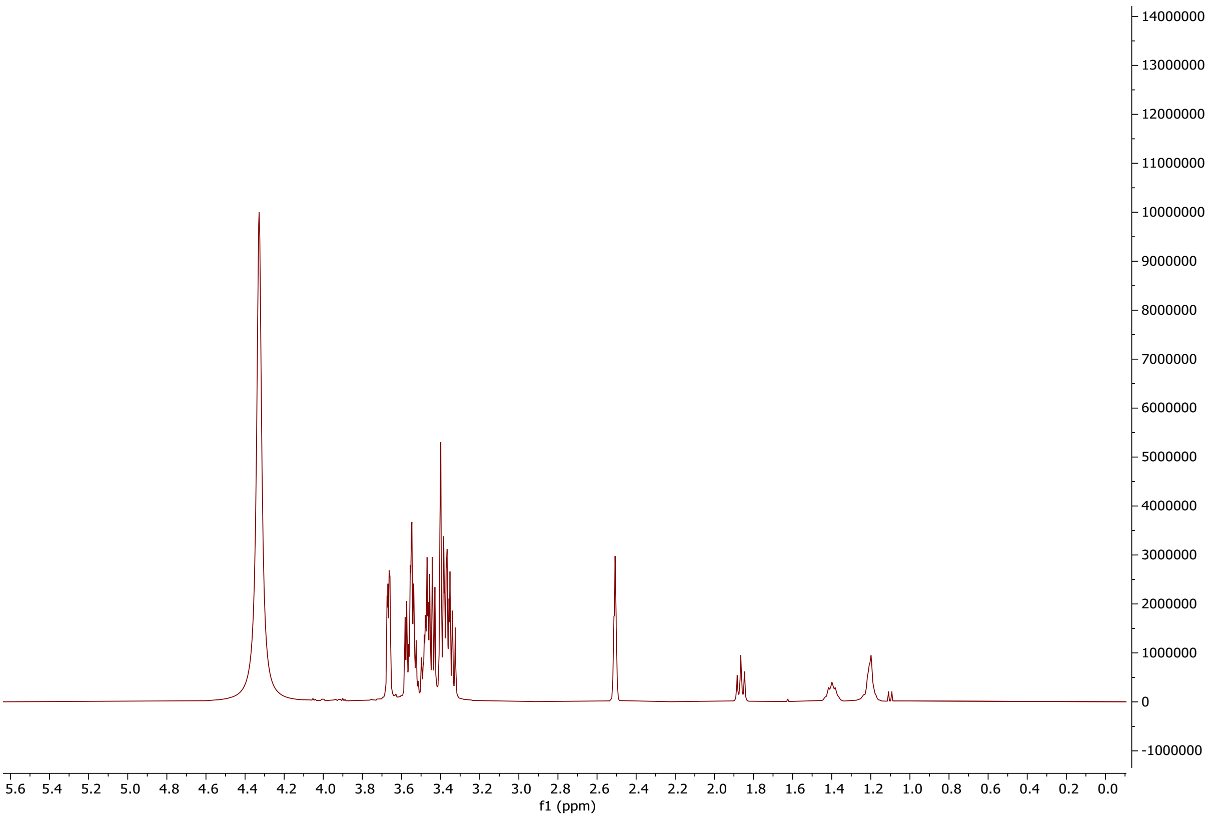

Figure S10. ^{1}H NMR spectrum of Polymer 4 (400 MHz, DMSO-d_6).

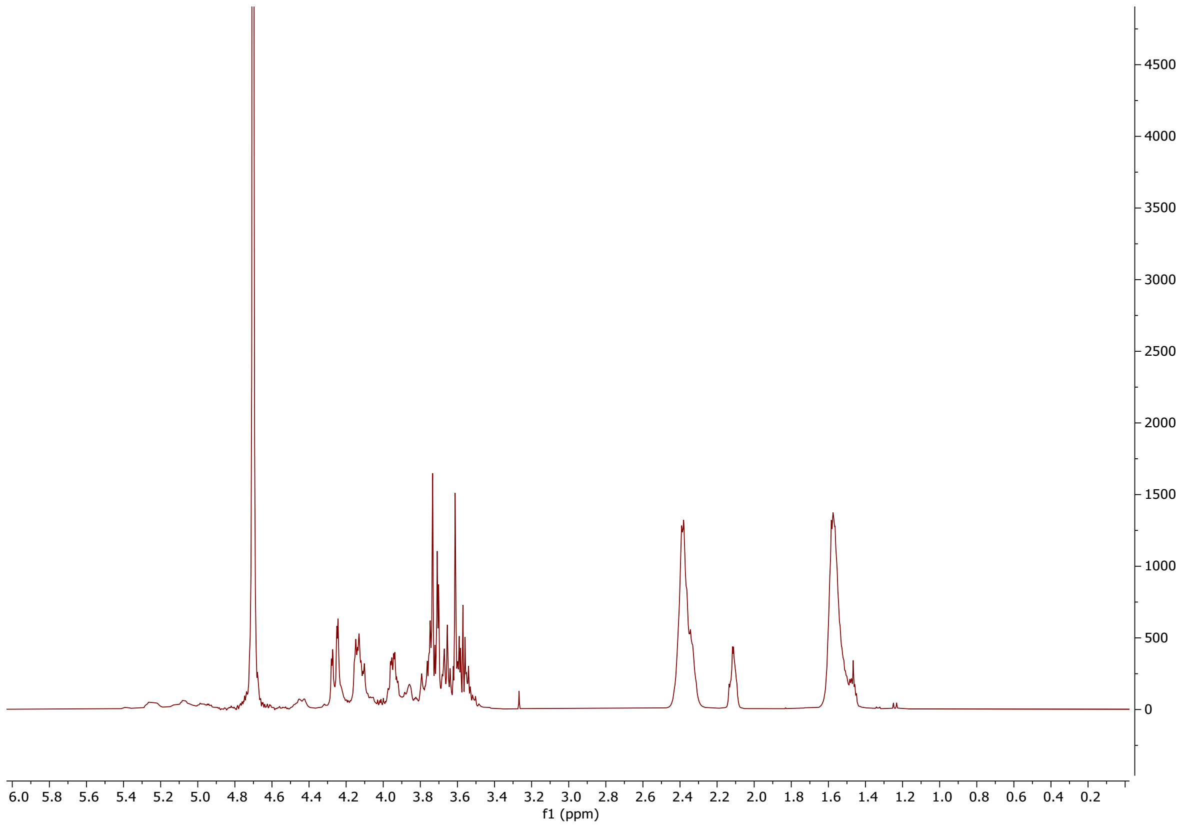

Figure S11. ^{1}H NMR spectrum of Polymer 5 (400 MHz, D_2O).

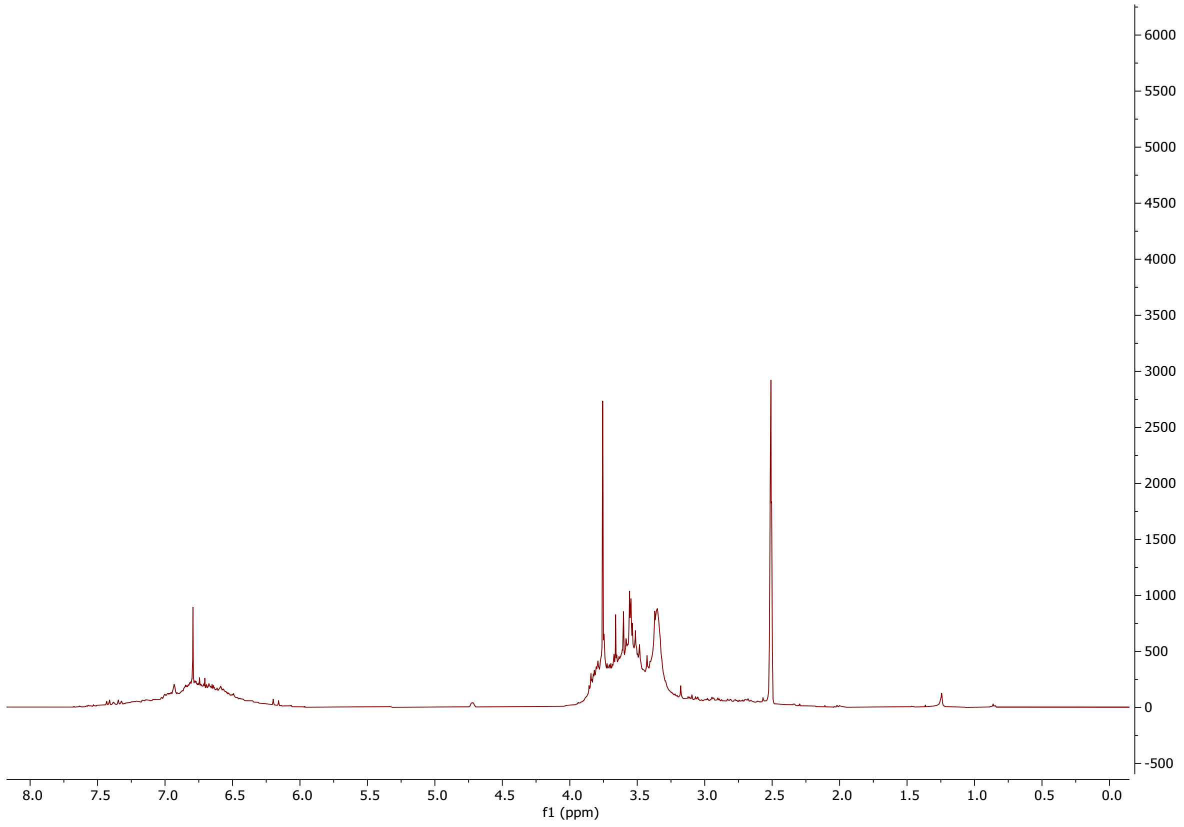

Figure S12. ^{1}H NMR spectrum of Polymer 6 (400 MHz, DMSO-d_6).

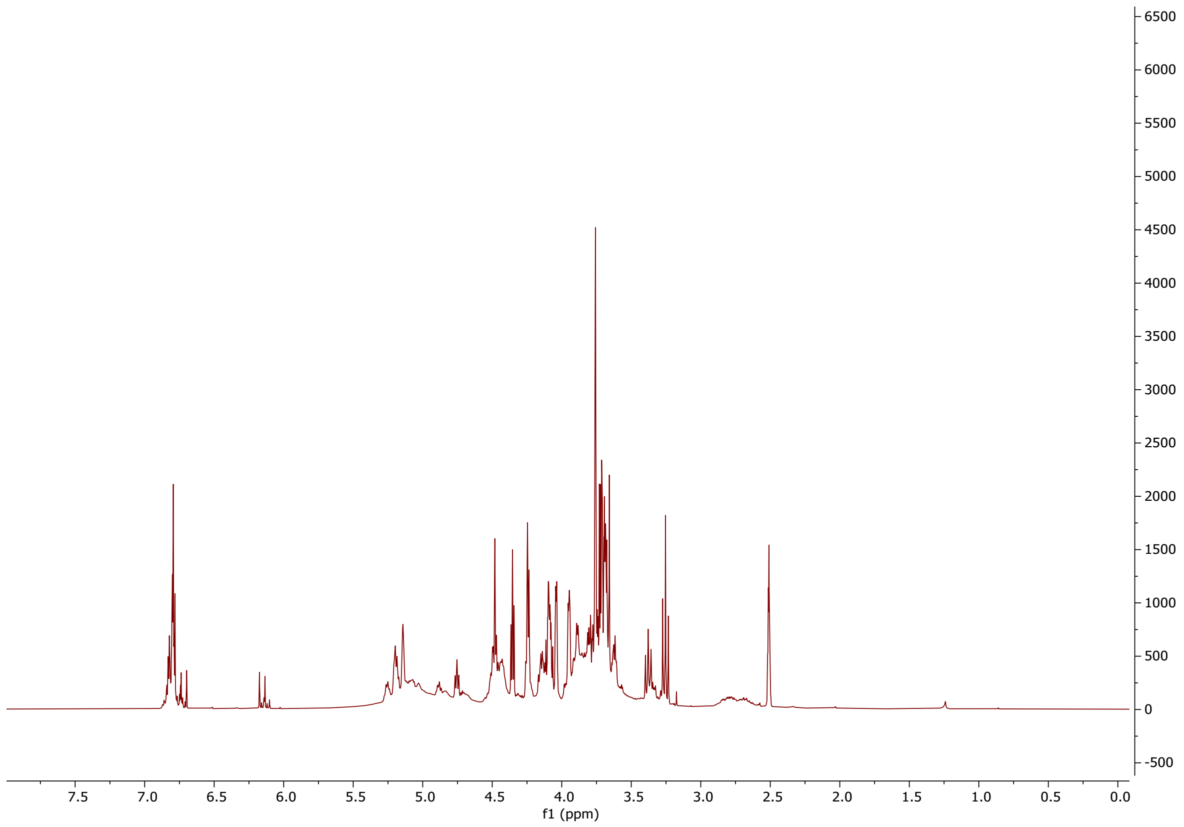

Figure S13. ^{1}H NMR spectrum of Polymer 7 in (400 MHz, DMSO-d_6).

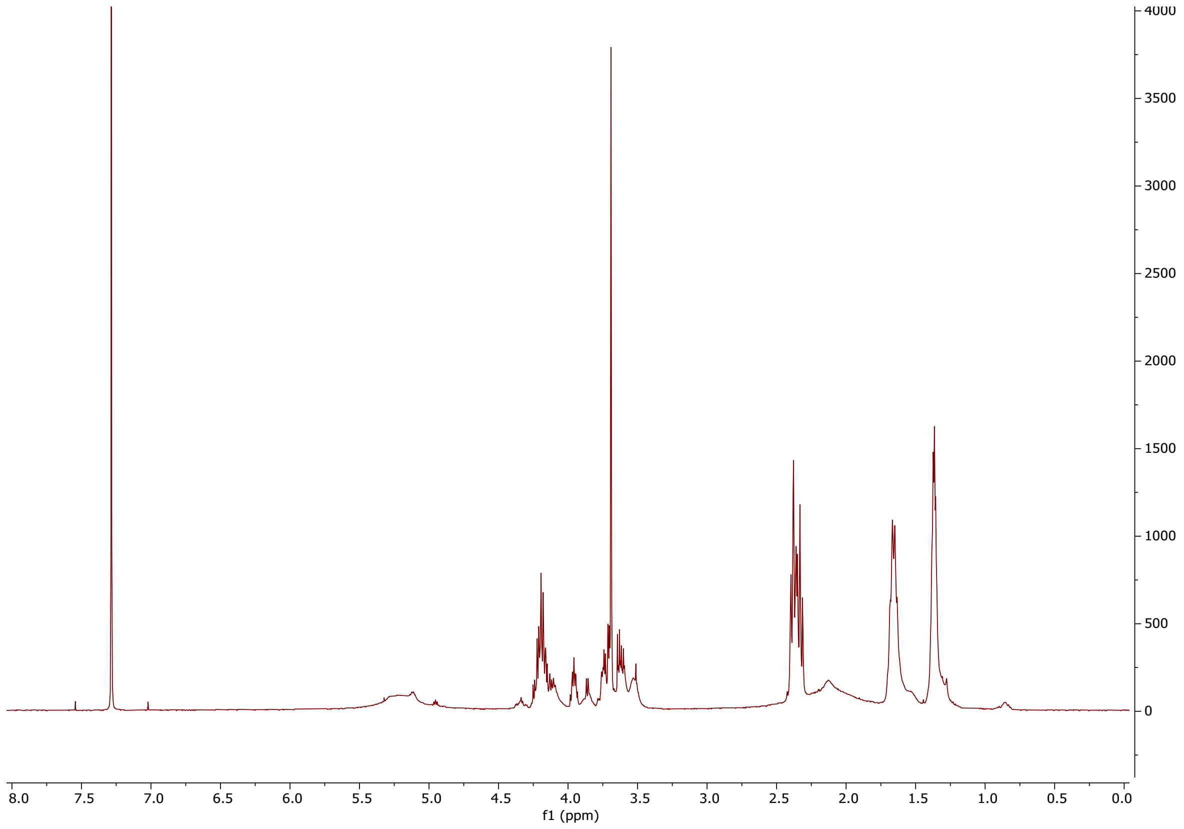

Figure S14. ^{1}H NMR spectrum of Polymer 8 (400 MHz, $CDCl_3$).

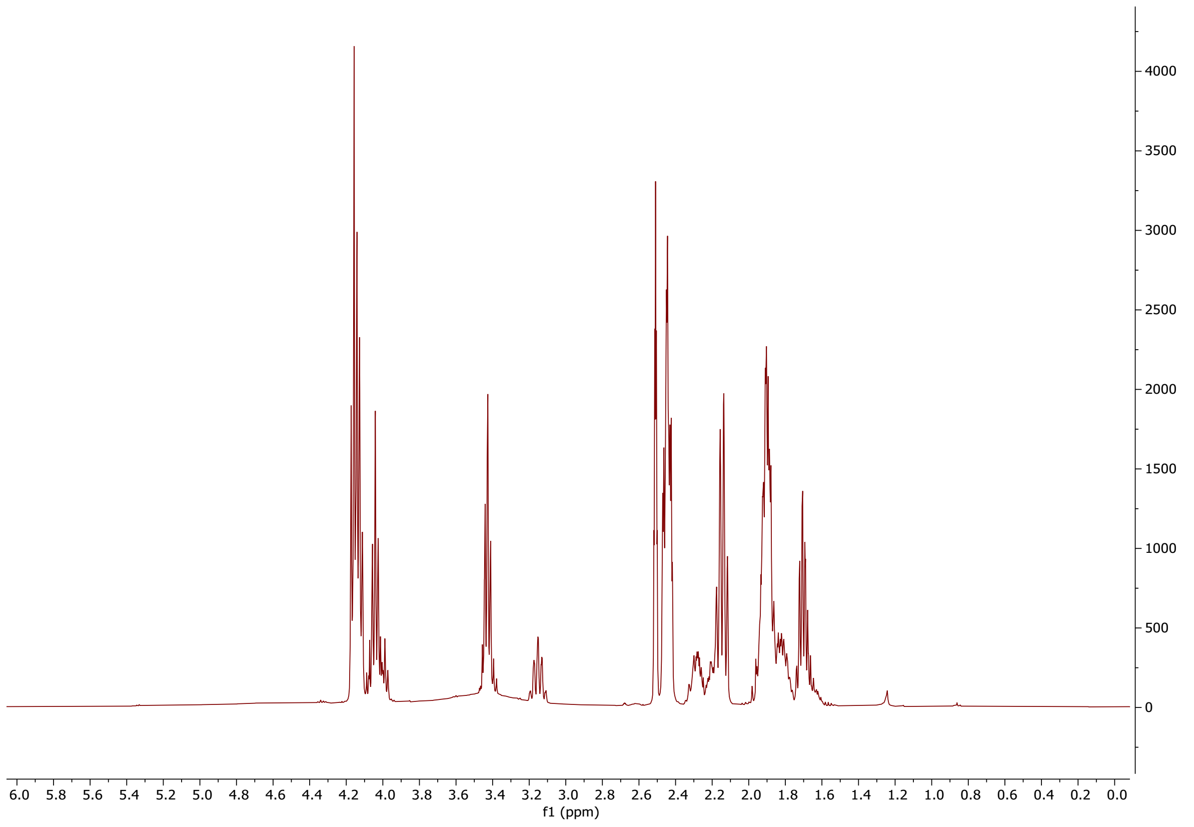

Figure S15. ^{1}H NMR spectrum of Polymer 9 (400 MHz, DMSO-d_6).

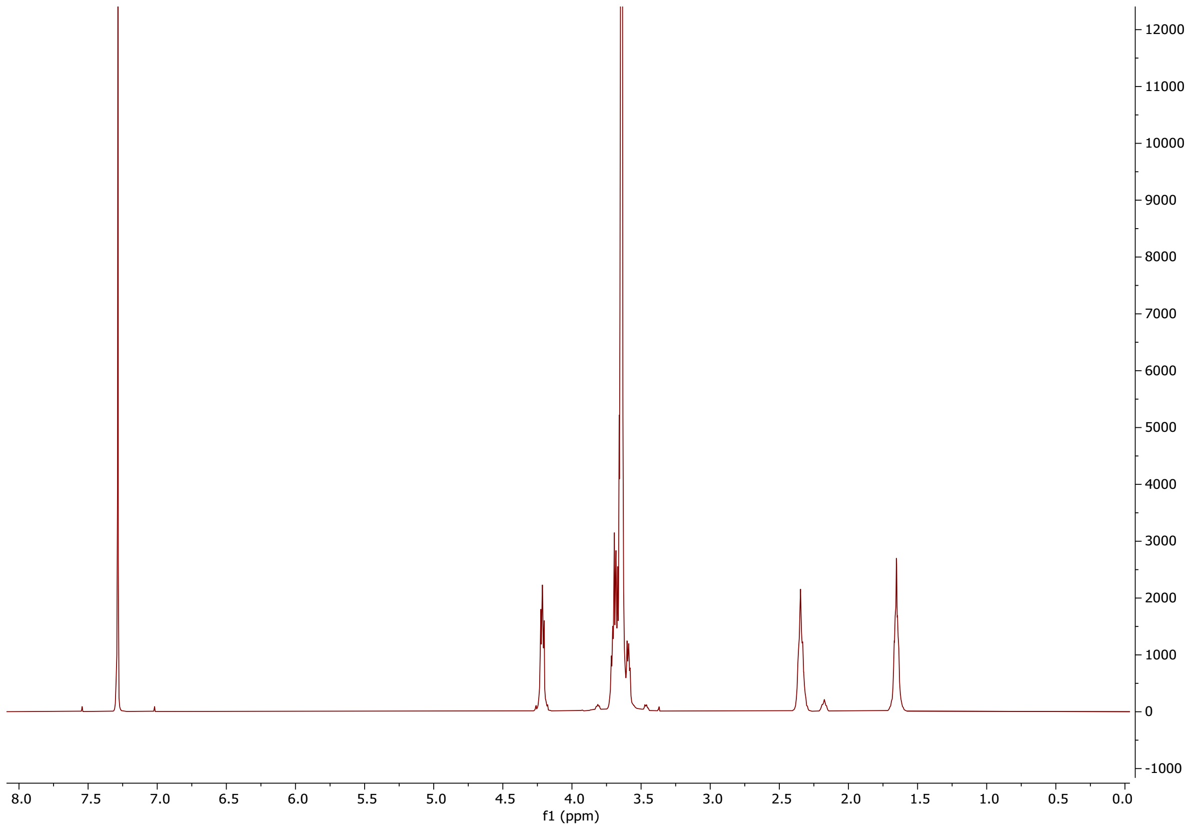

Figure S16. ^{1}H NMR spectrum of Polymer 10 (400 MHz, $CDCl_3$).

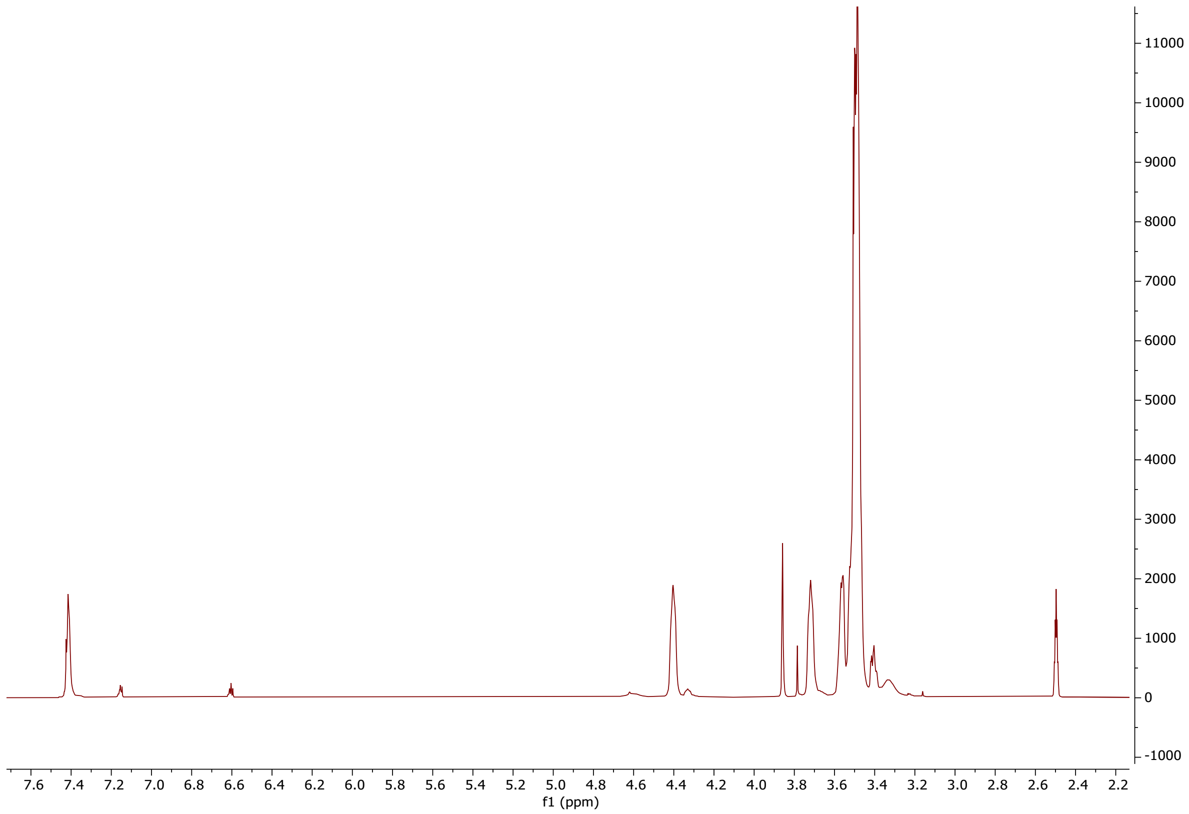

Figure S17. ^{1}H NMR spectrum of Polymer 11 ((400 MHz, DMSO-d_6).

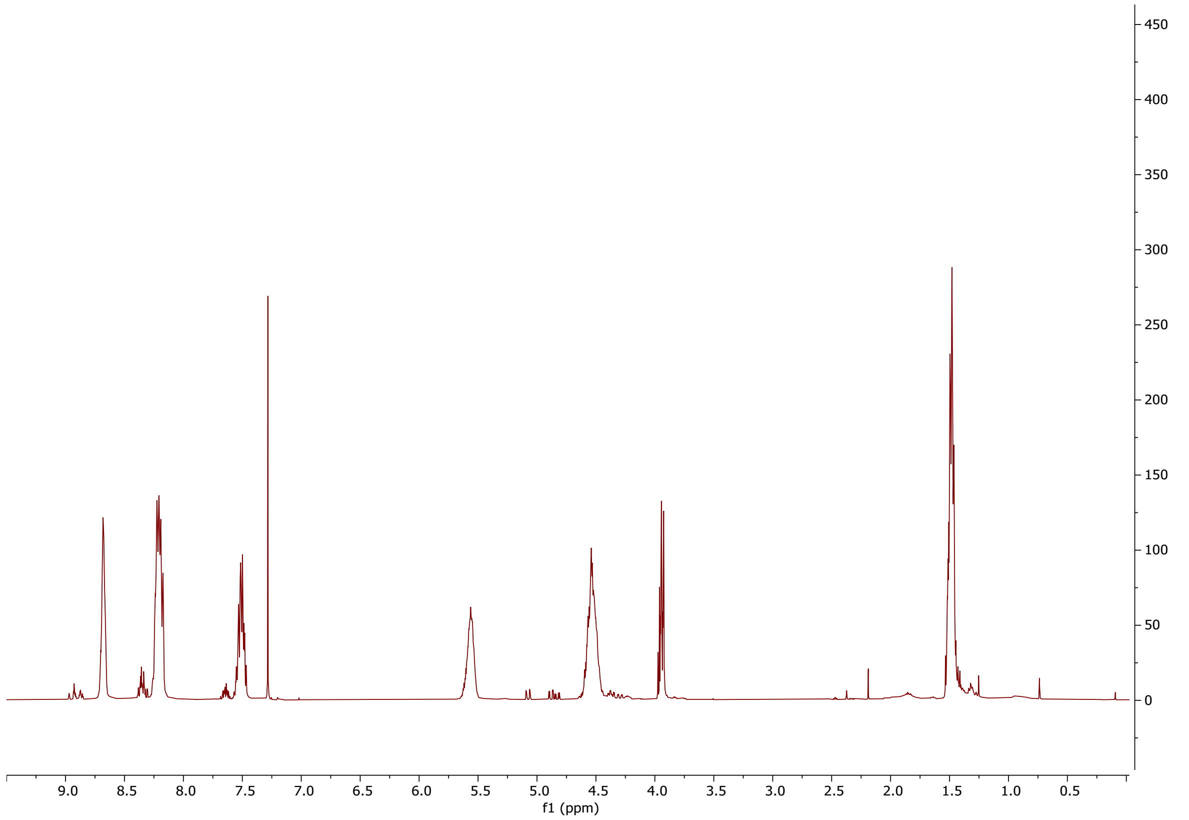

Figure S18. ^{1}H NMR spectrum of Polymer 12 (400 MHz, $CDCl_3$).

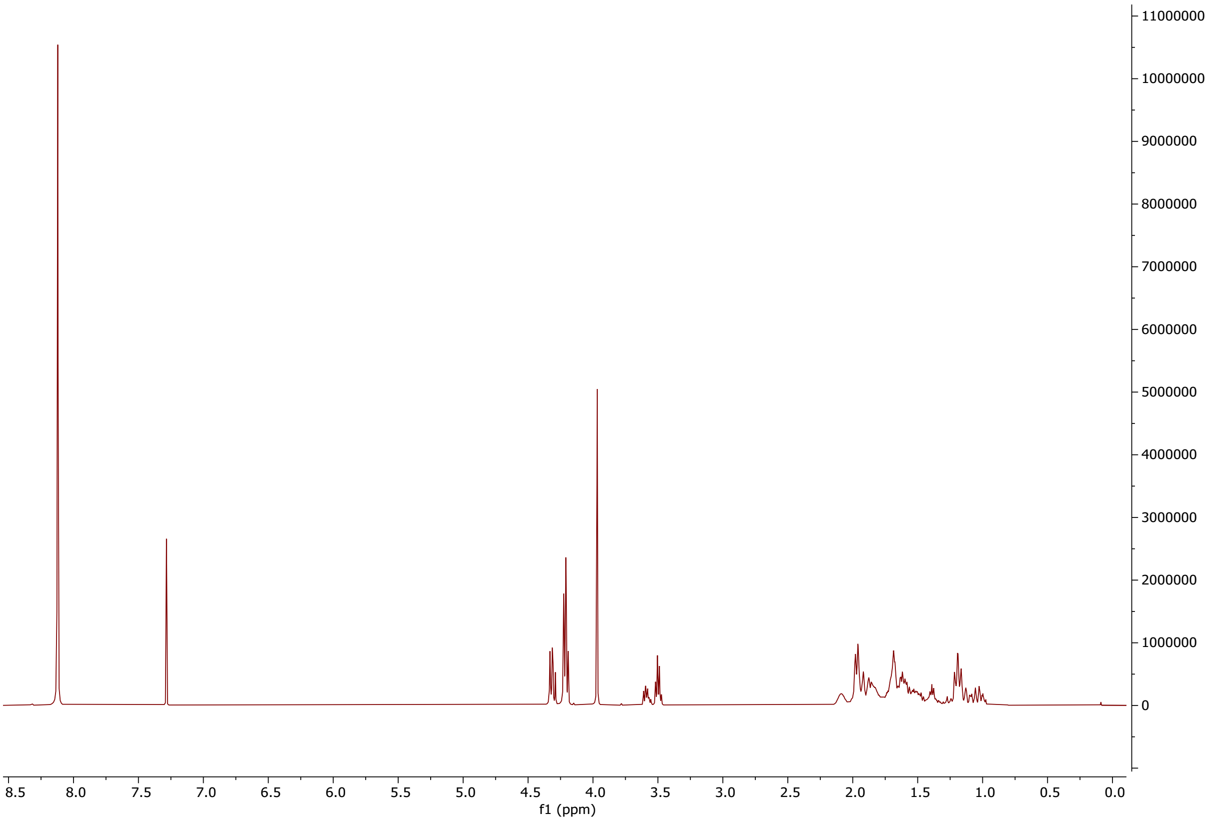

Figure S19. ^{1}H NMR spectrum of Polymer 13 (400 MHz, $CDCl_3$).

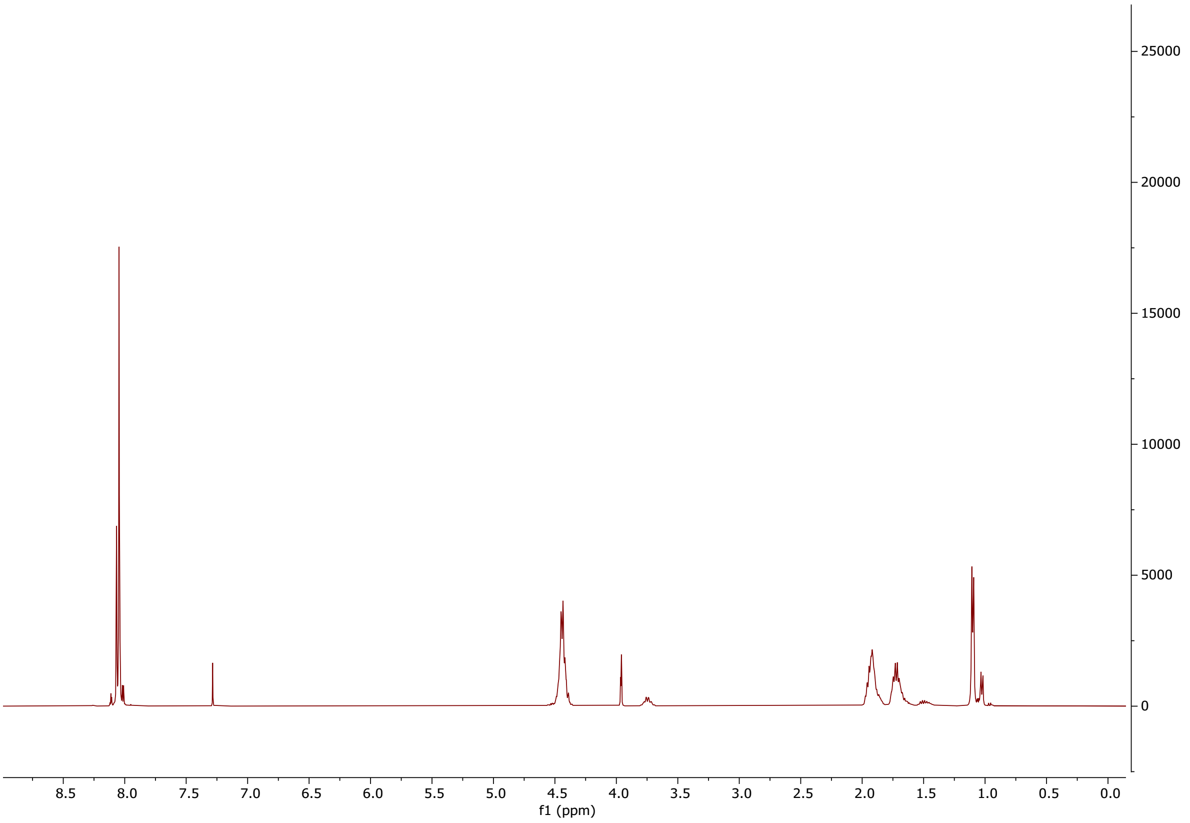

Figure S20. ^{1}H NMR spectrum of Polymer 14 (400 MHz, $CDCl_3$).

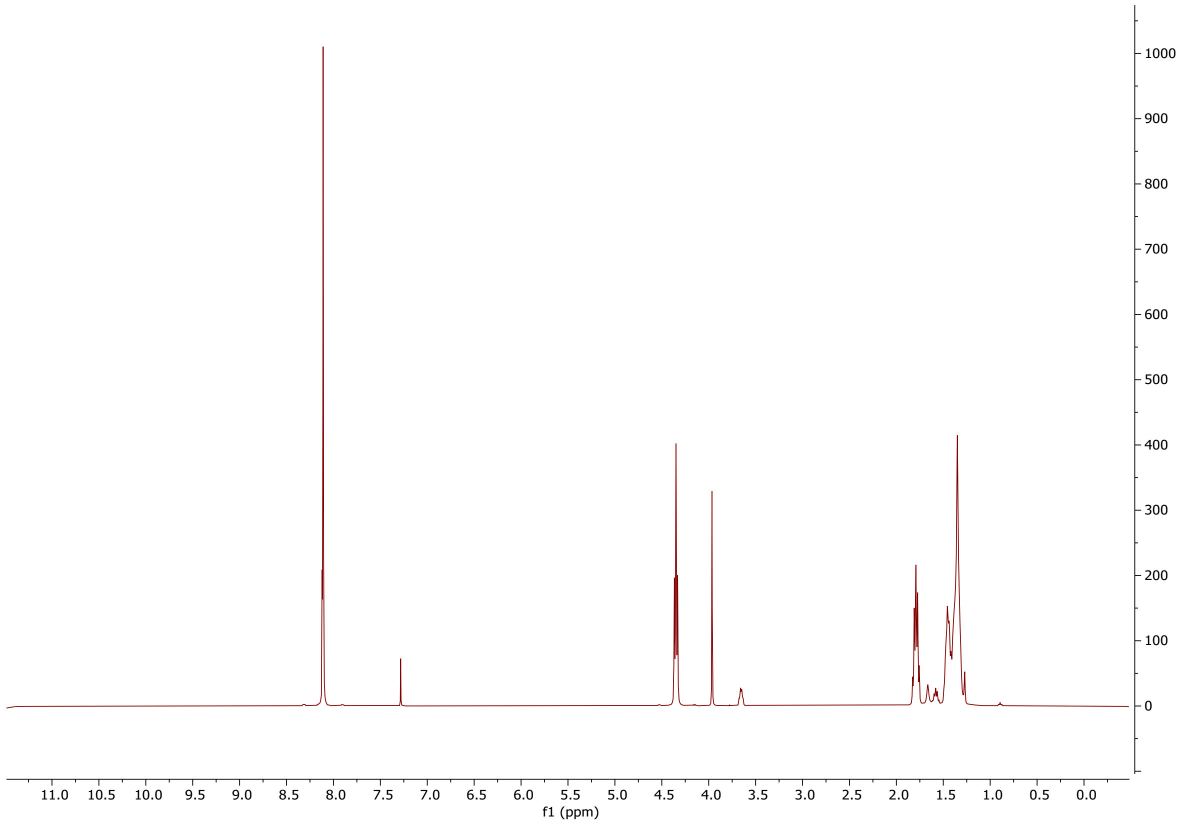

Figure S21. ^{1}H NMR spectrum of Polymer 15 (400 MHz, $CDCl_3$).

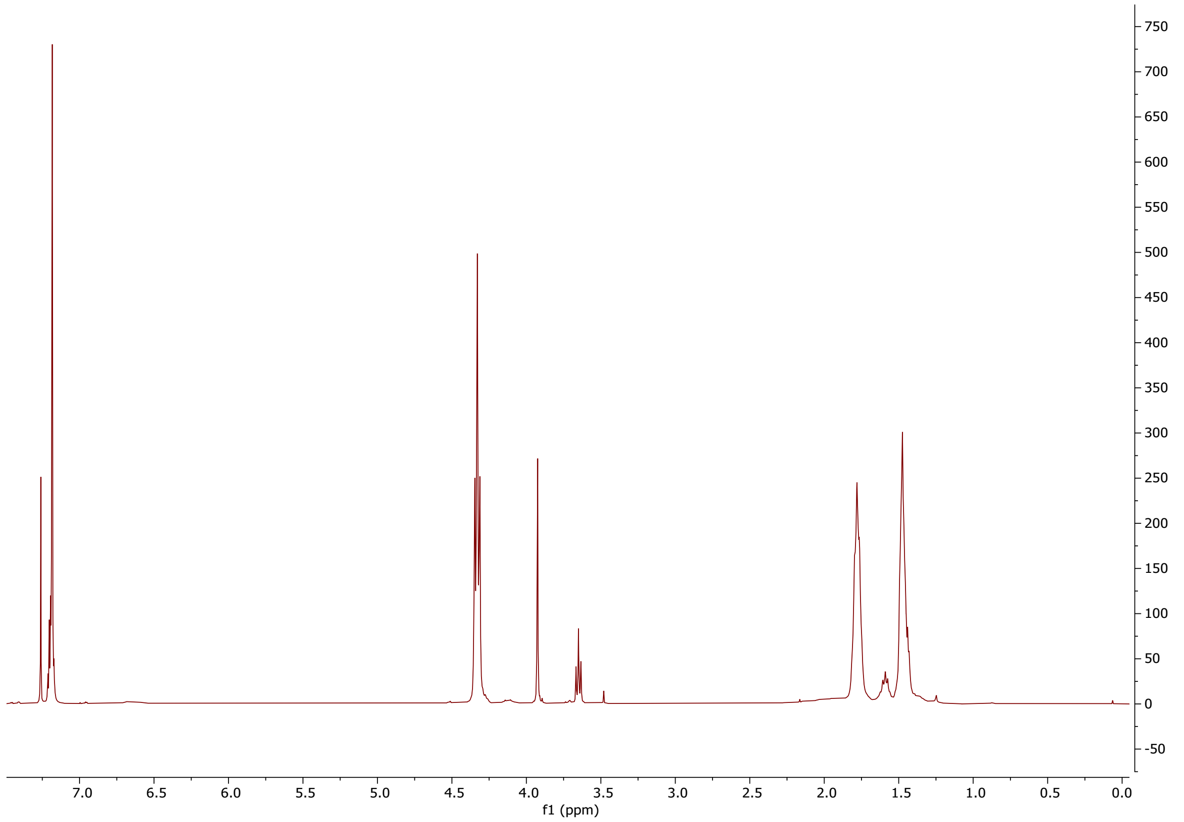

Figure S22. ^{1}H NMR spectrum of Polymer 16 (400 MHz, $CDCl_3$).

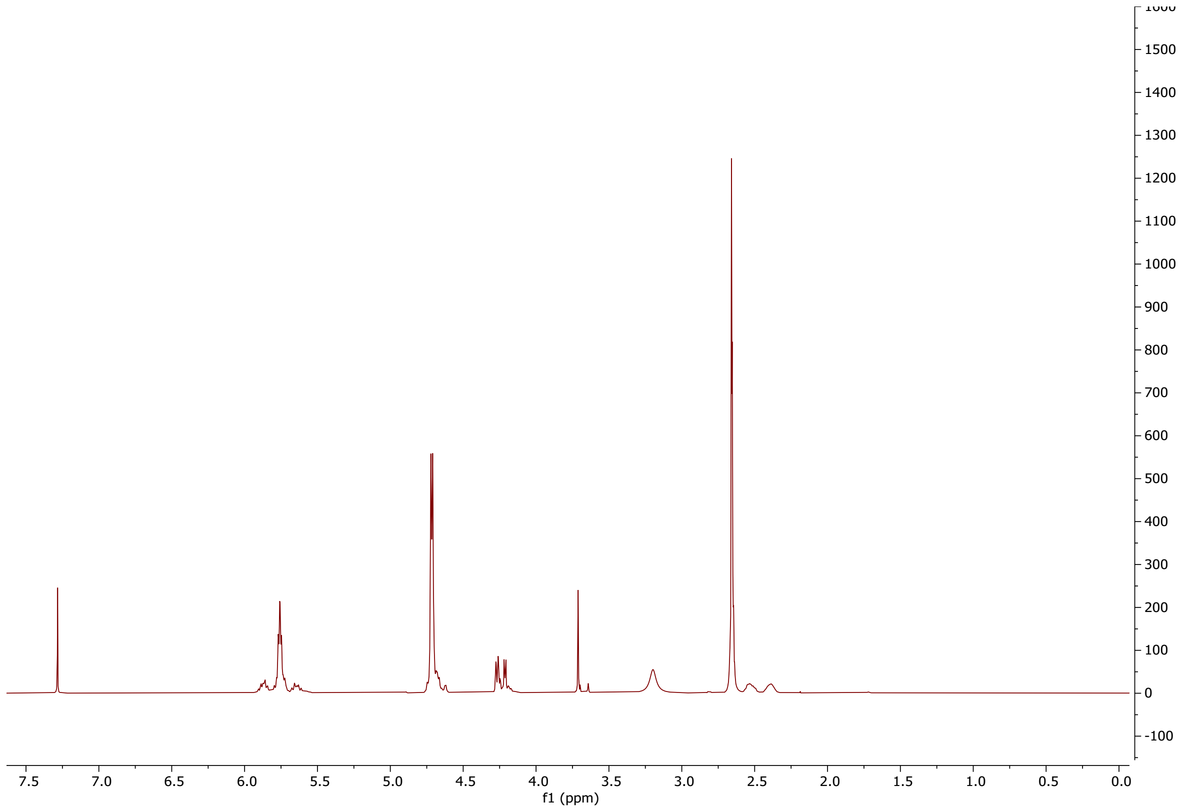

Figure S23. ^{1}H NMR spectrum of Polymer 17 (400 MHz, $CDCl_3$).

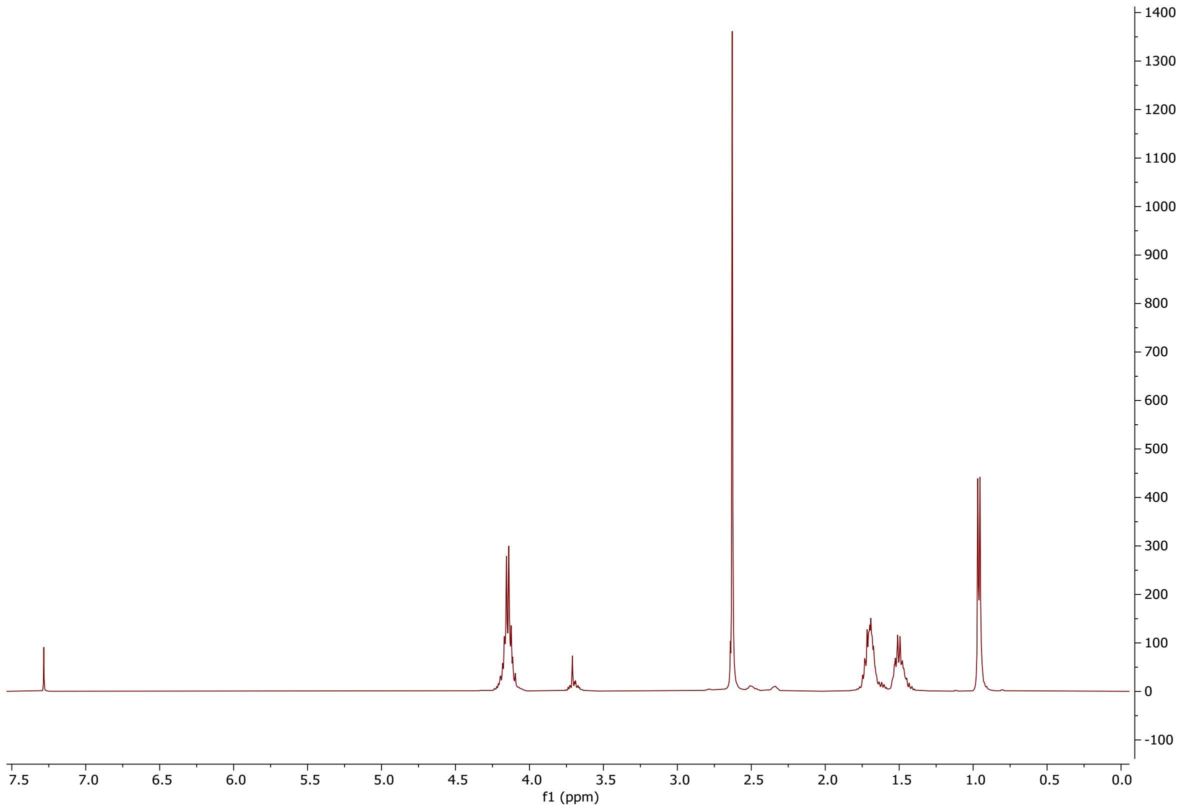

Figure S24. ^{1}H NMR spectrum of Polymer 18 (400 MHz, $CDCl_3$).

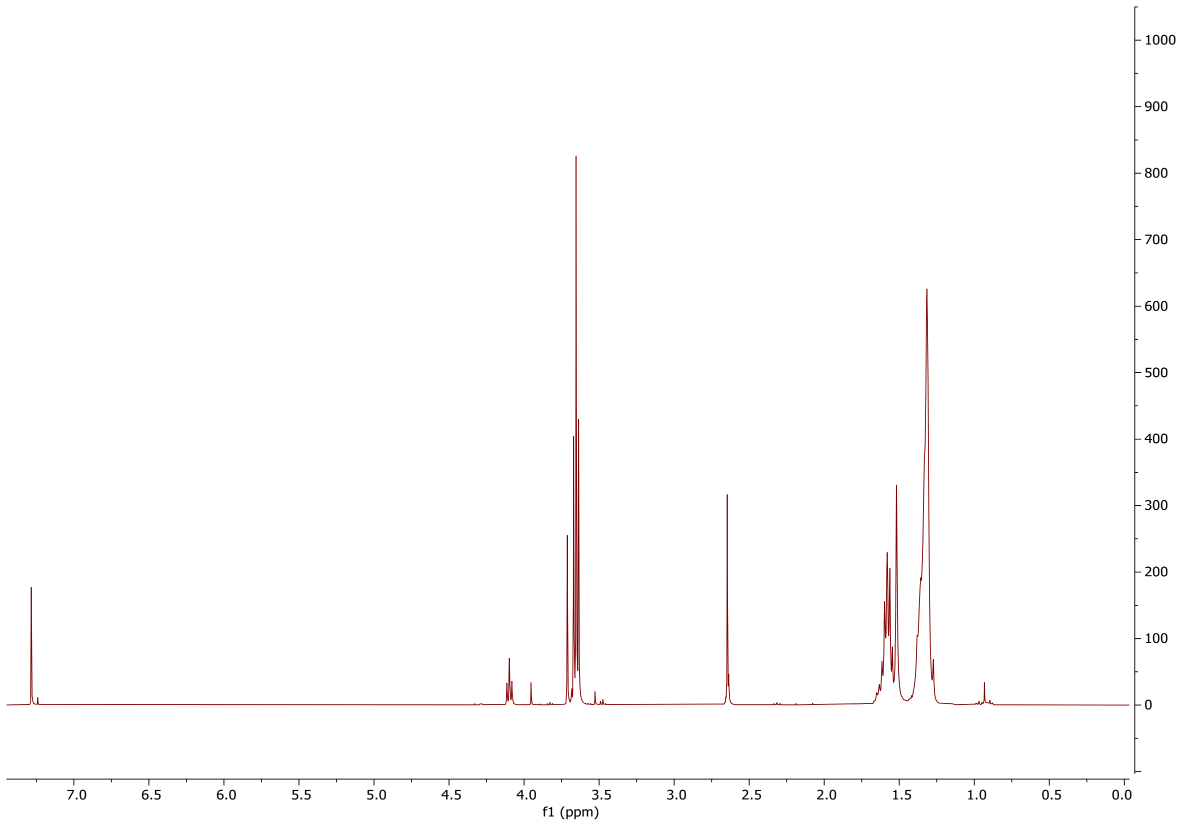

Figure S25. ^{1}H NMR spectrum of Polymer 19 (400 MHz, $CDCl_3$).

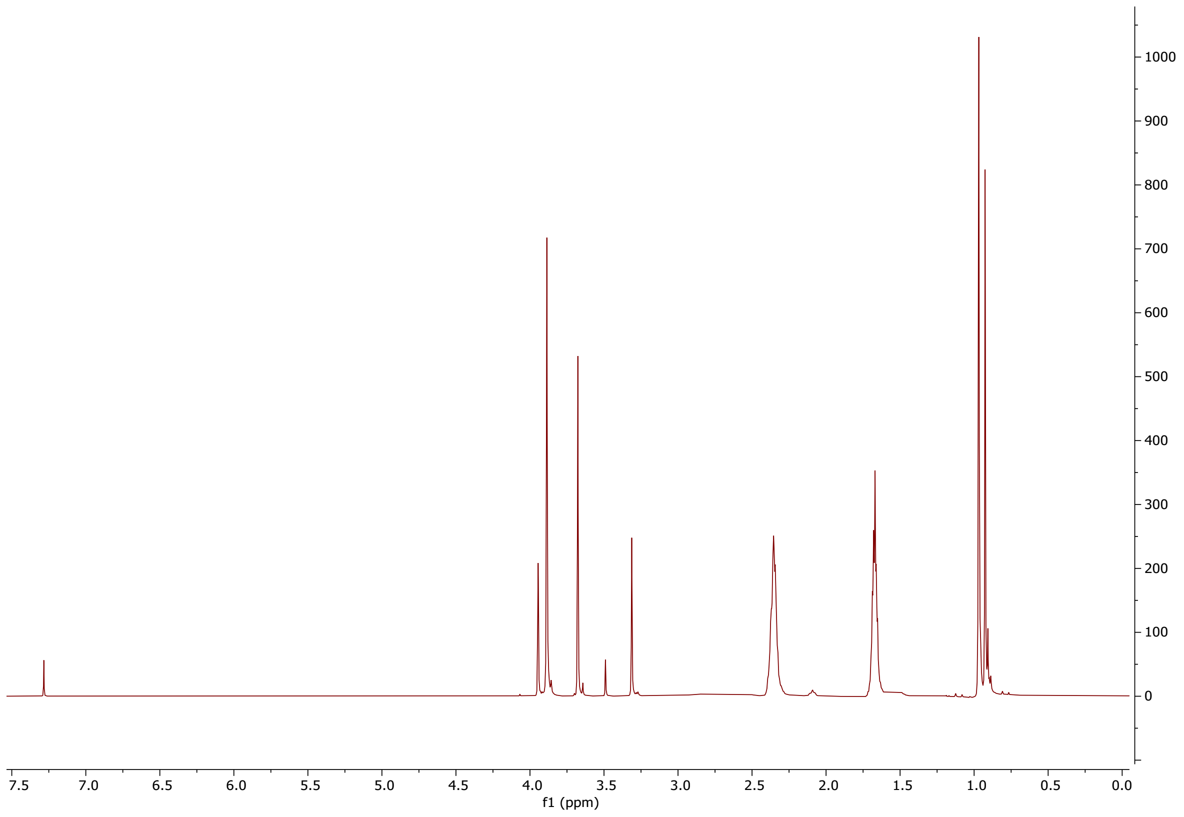

Figure S26. ^{1}H NMR spectrum of Polymer 20 (400 MHz, $CDCl_3$).

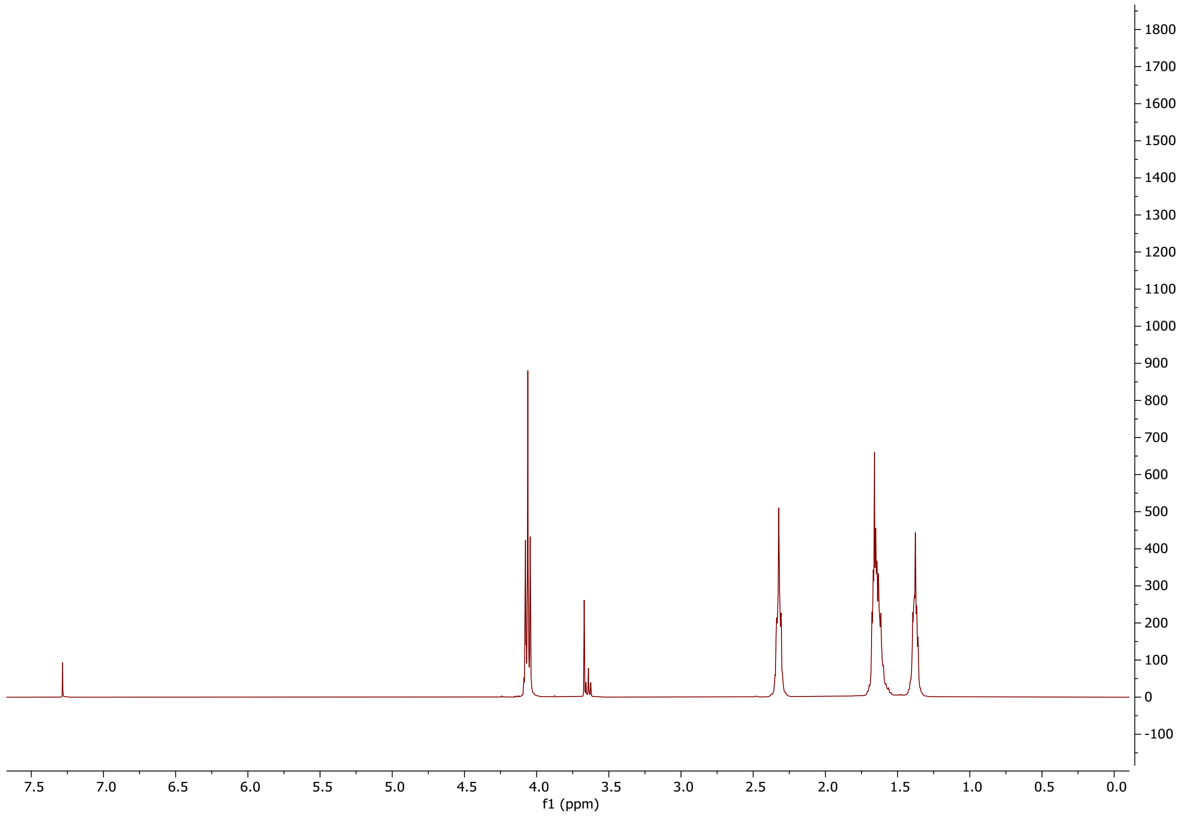

Figure S27. ^{1}H NMR spectrum of Polymer 21 (400 MHz, $CDCl_3$).

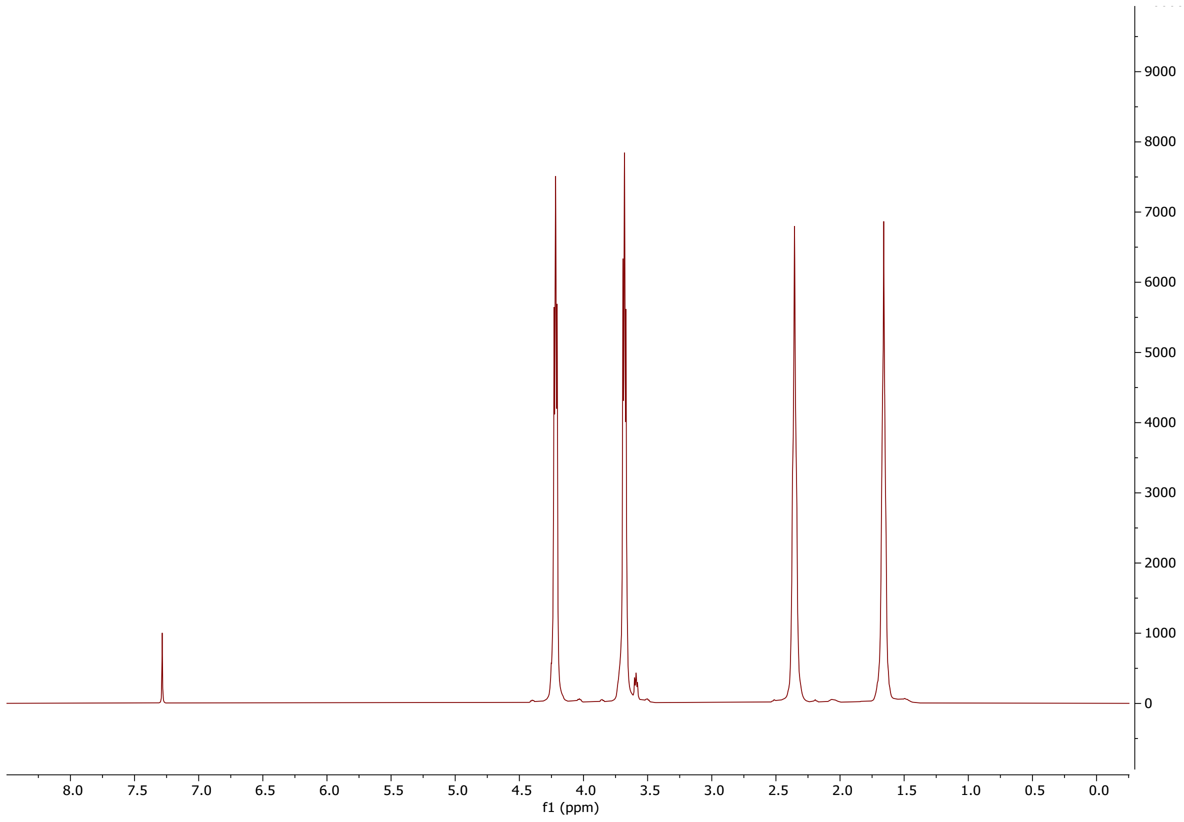

Figure S28. ^{1}H NMR spectrum of Polymer 22 (400 MHz, $CDCl_3$).

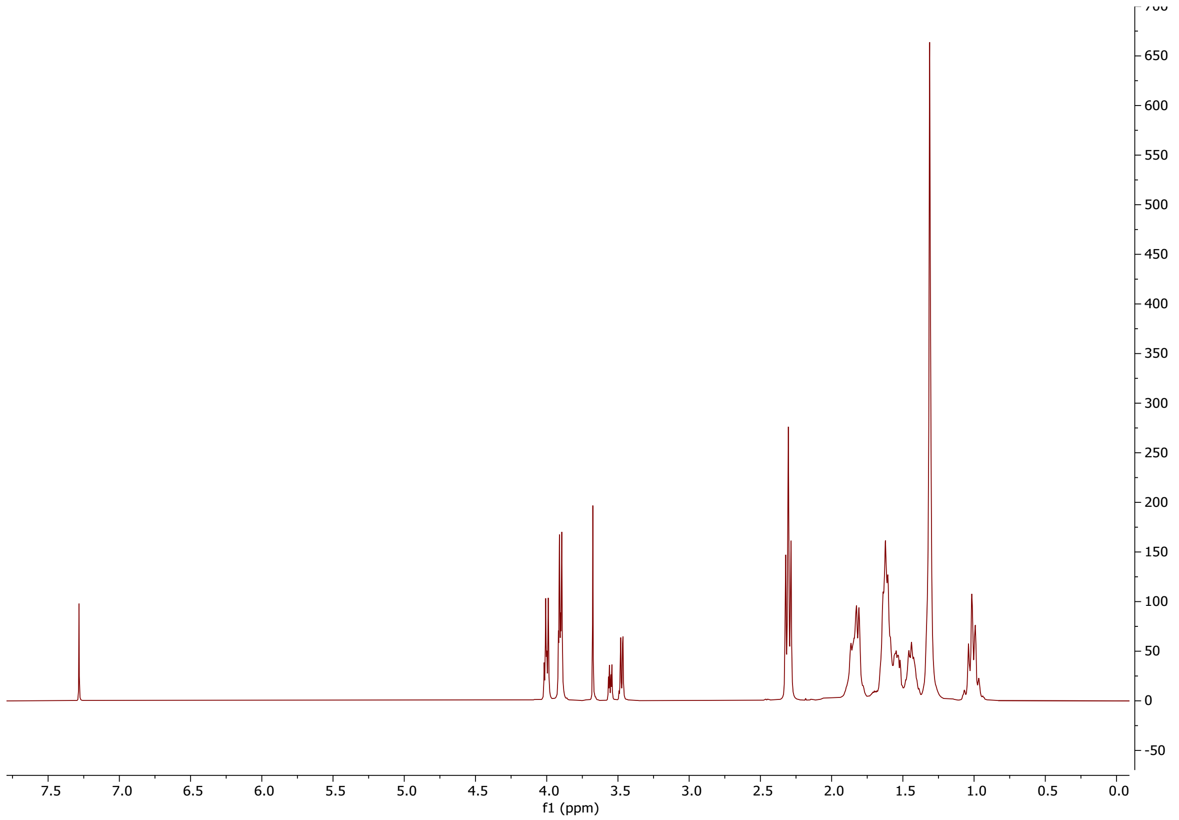

Figure S29. ^{1}H NMR spectrum of Polymer 23 (400 MHz, $CDCl_3$).

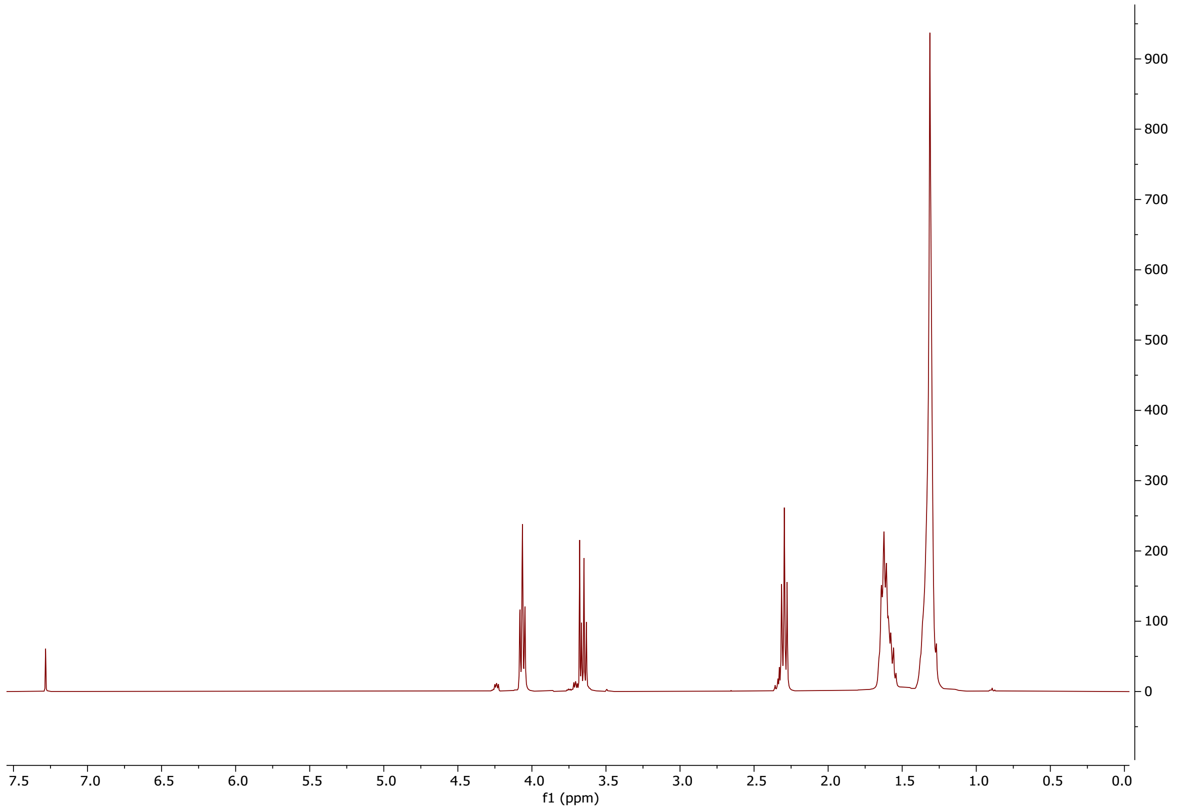

Figure S30. ^{1}H NMR spectrum of Polymer 24 (400 MHz, $CDCl_3$).

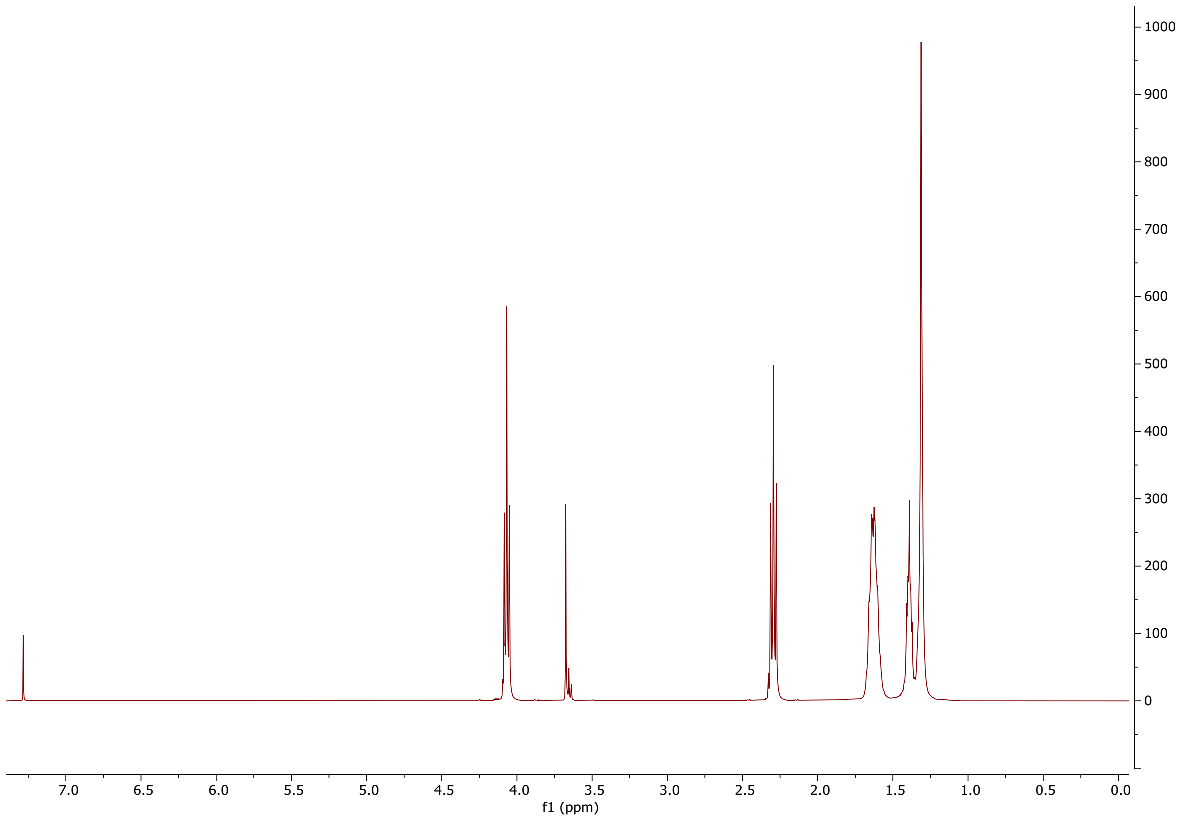

Figure S31. ^{1}H NMR spectrum of Polymer 25 (400 MHz, $CDCl_3$).

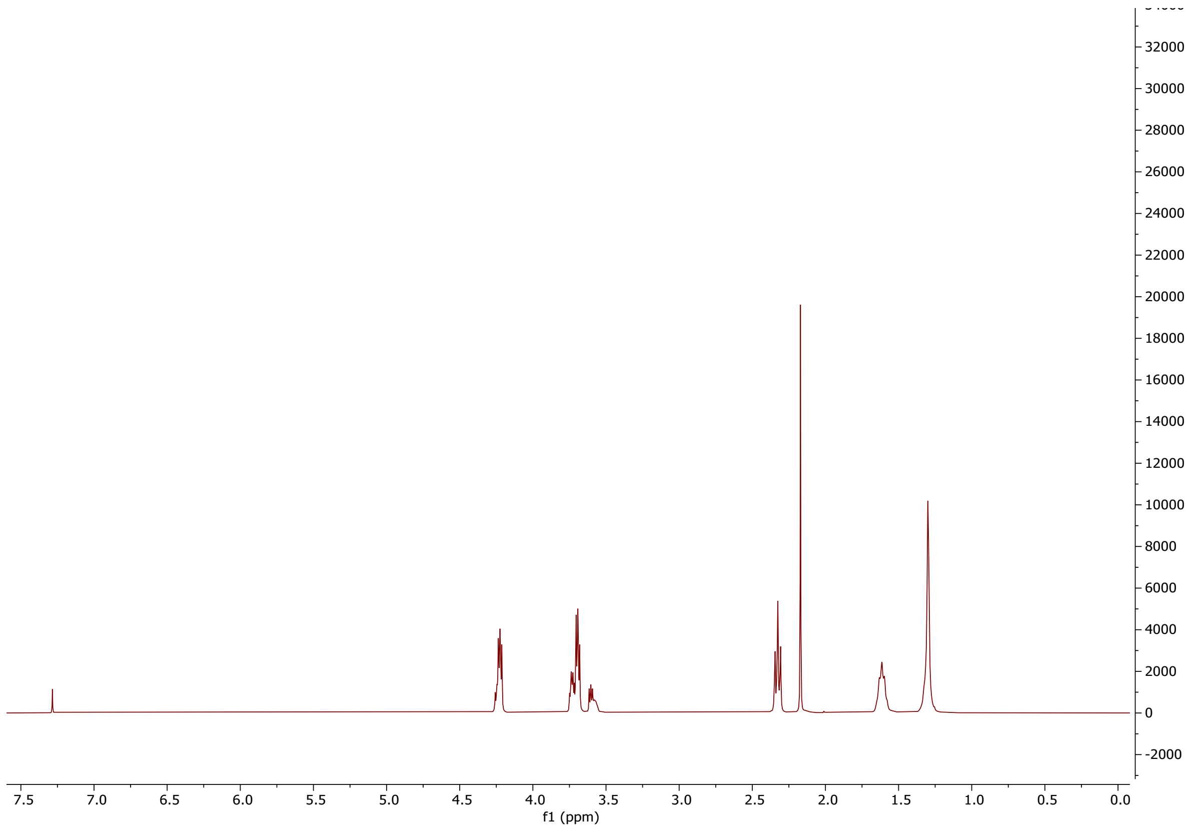

Figure S32. ^{1}H NMR spectrum of Polymer 26 (400 MHz, $CDCl_3$).

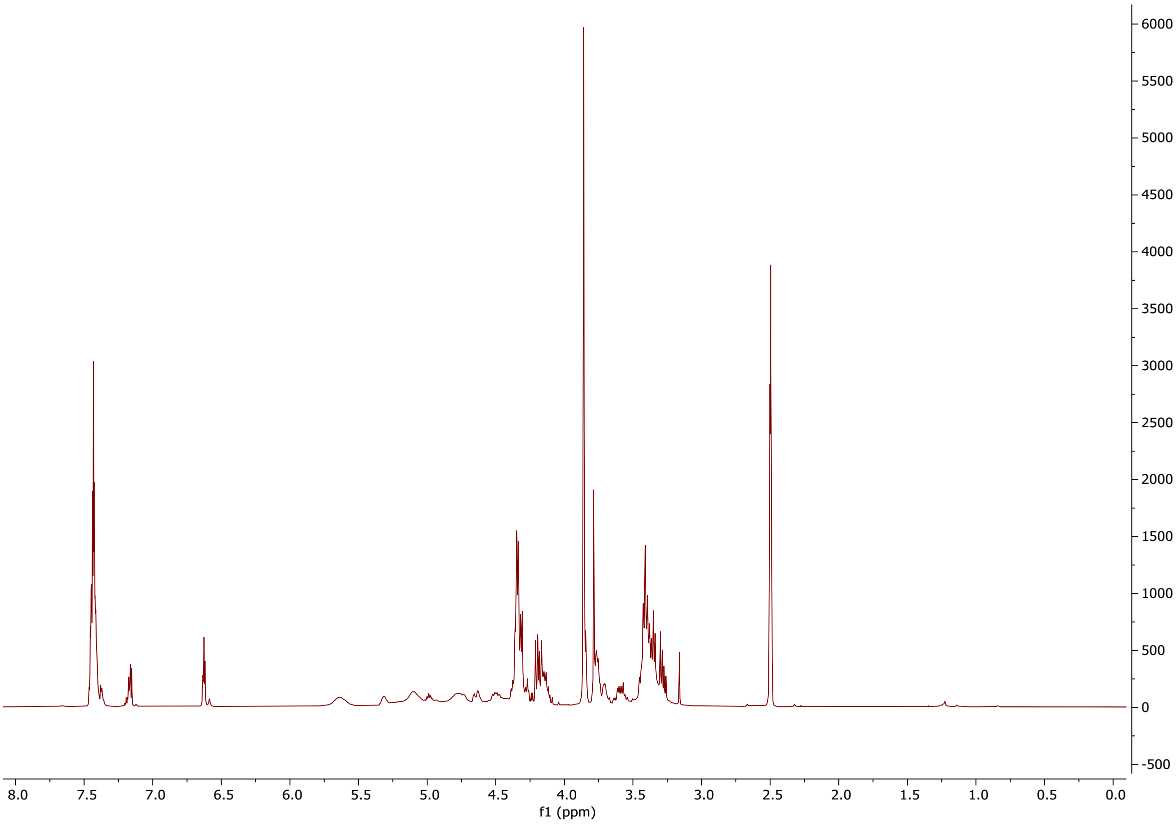

Figure S33. ^{1}H NMR spectrum of Polymer 27 in (400 MHz, DMSO-d_6).

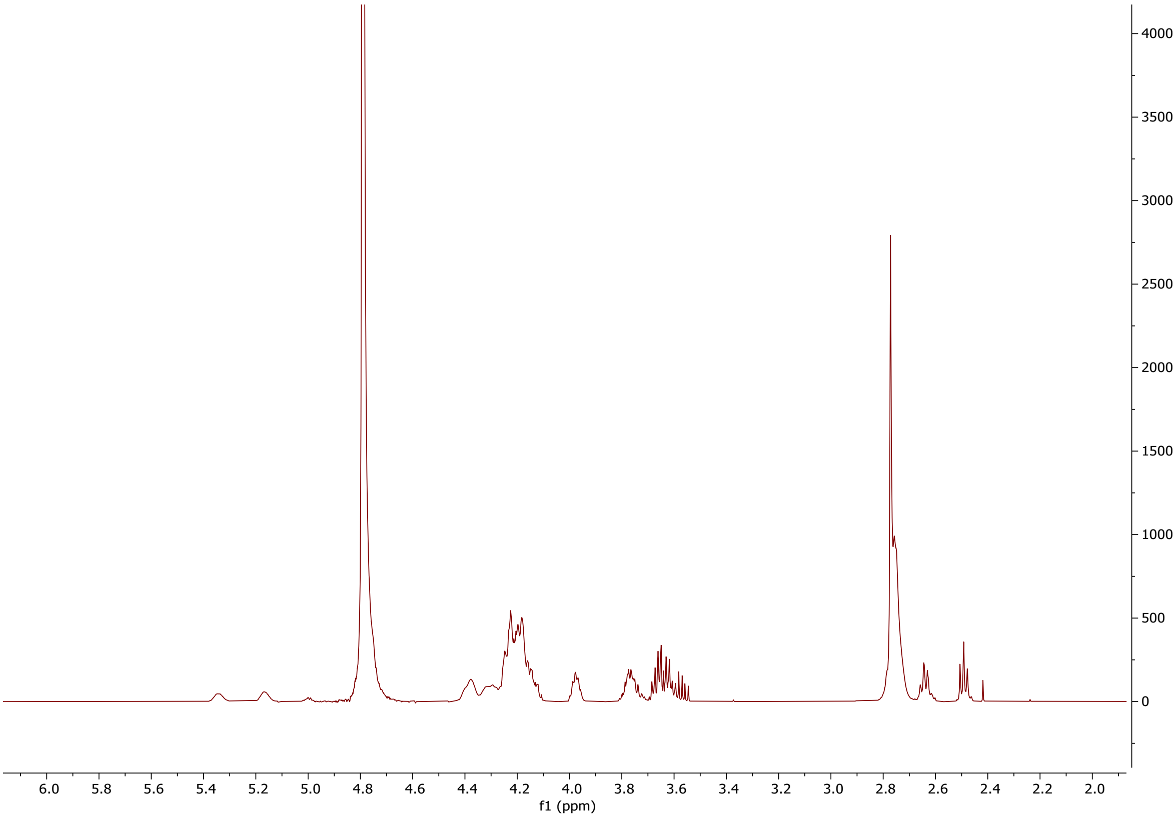

Figure S34. ^{1}H NMR spectrum of Polymer 28 (400 MHz, D_2O).

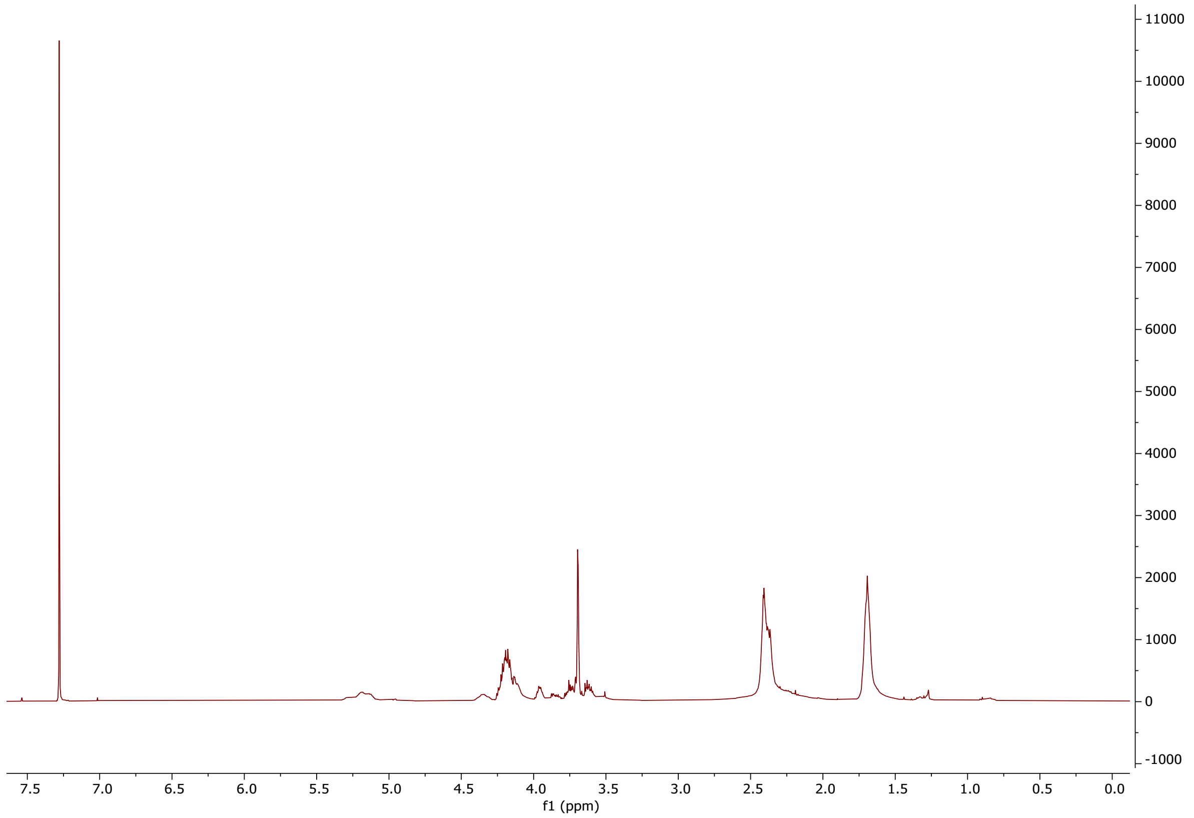

Figure S35. ^{1}H NMR spectrum of Polymer 29 in (400 MHz, $CDCl_3$).

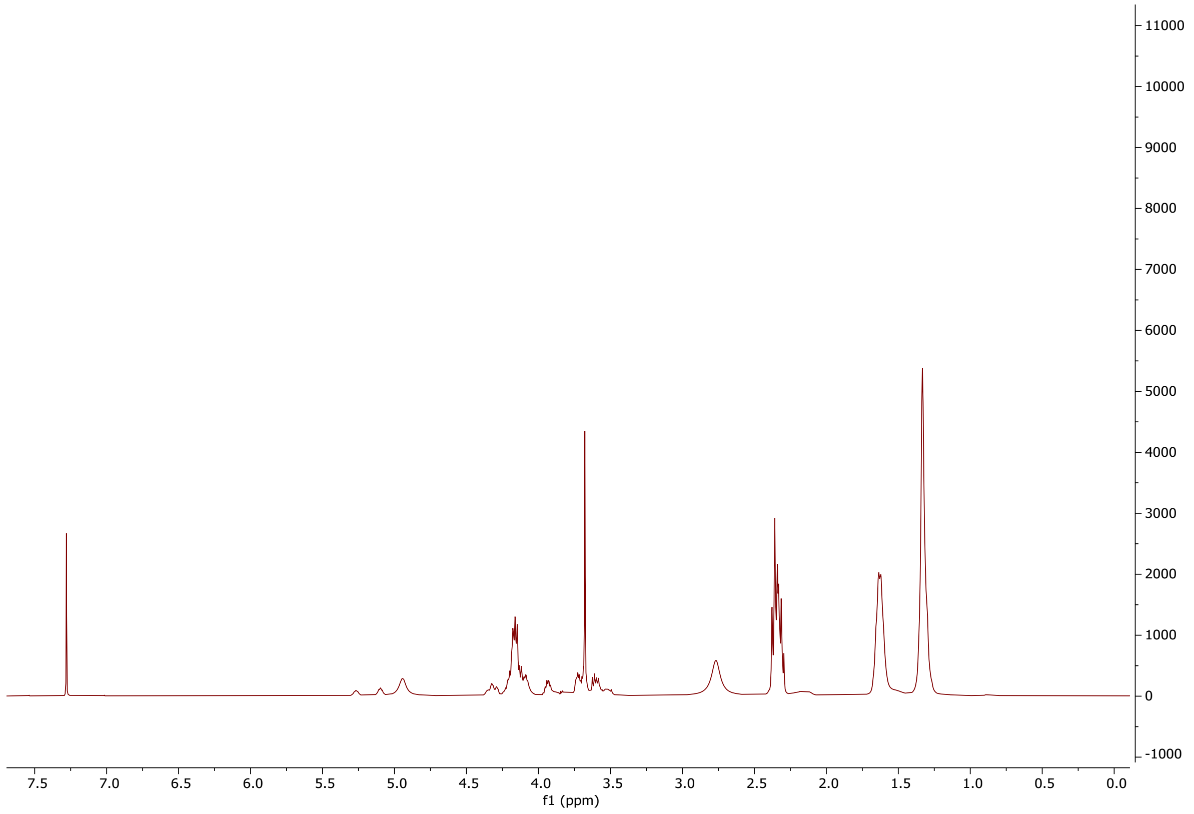

Figure S36. ^{1}H NMR spectrum of Polymer 30 (400 MHz, $CDCl_3$).

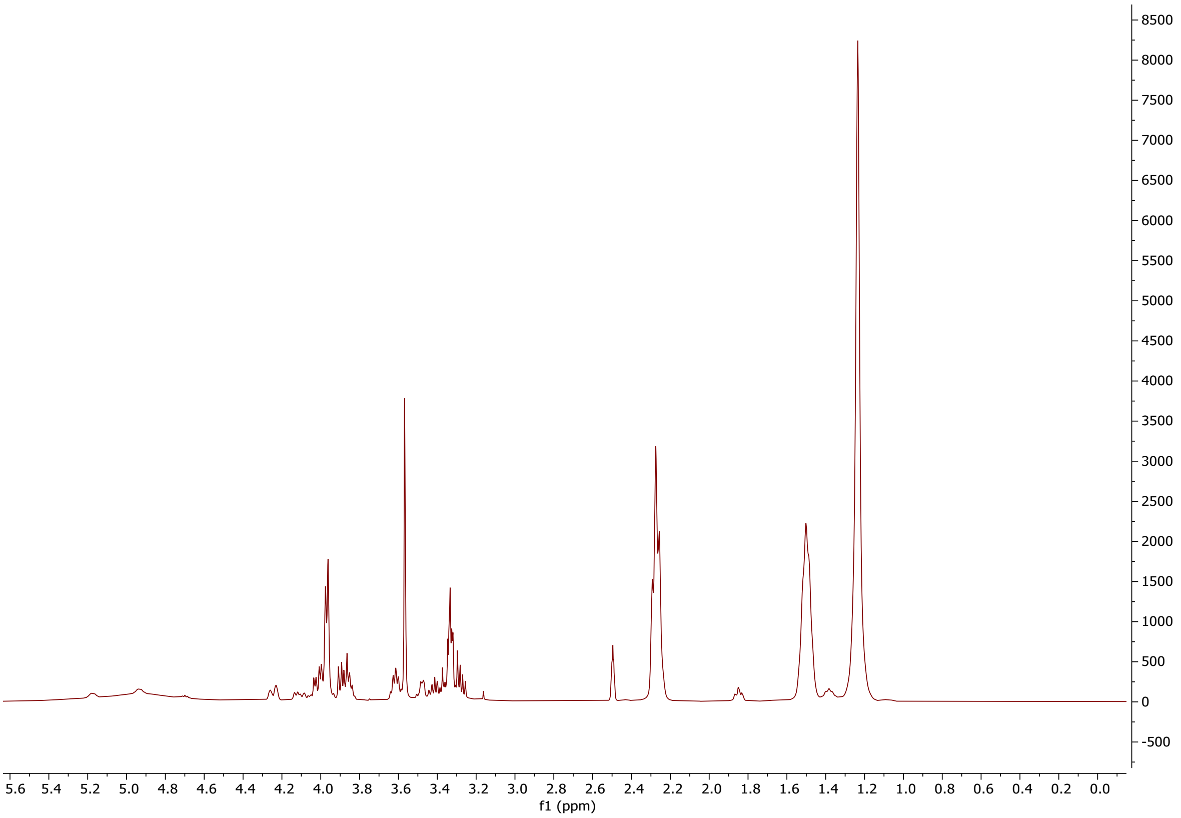

Figure S37. ^{1}H NMR spectrum of Polymer 31 (400 MHz DMSO-d_6).

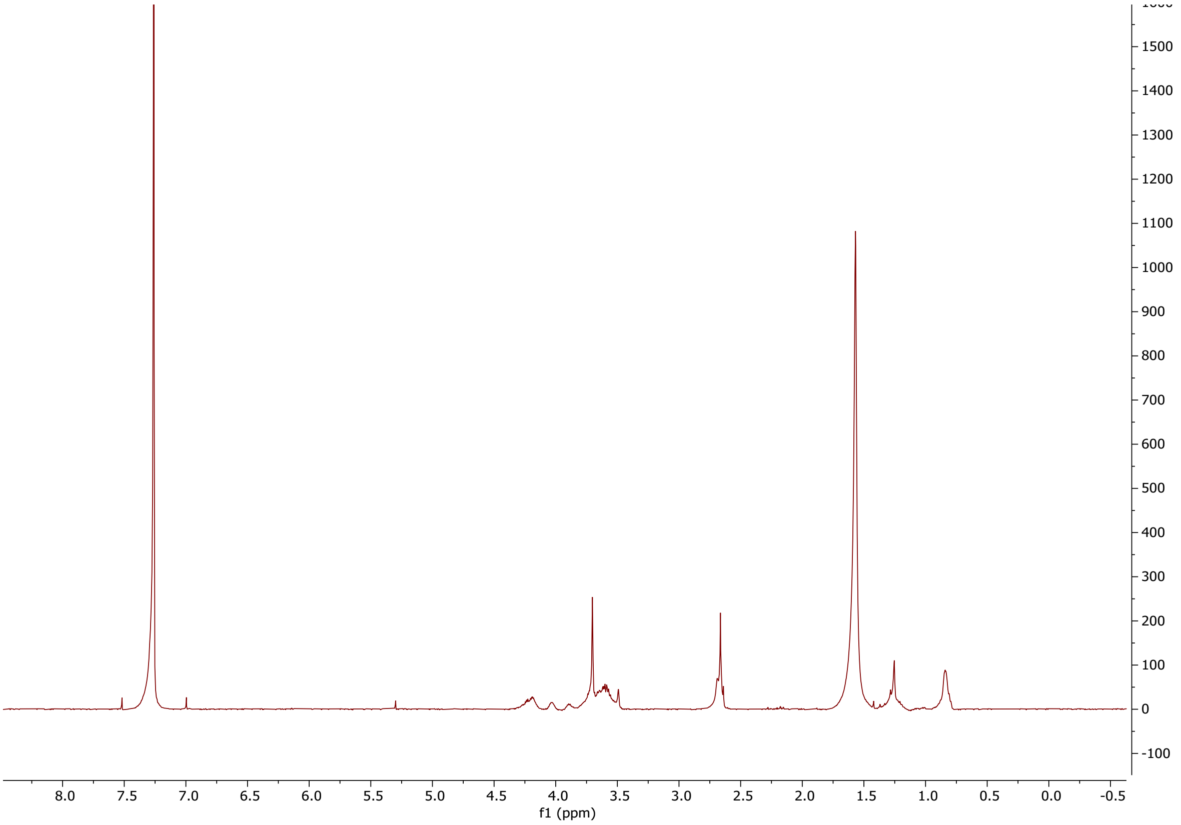

Figure S38. ^{1}H NMR spectrum of Polymer 32 (400 MHz, $CDCl_3$).

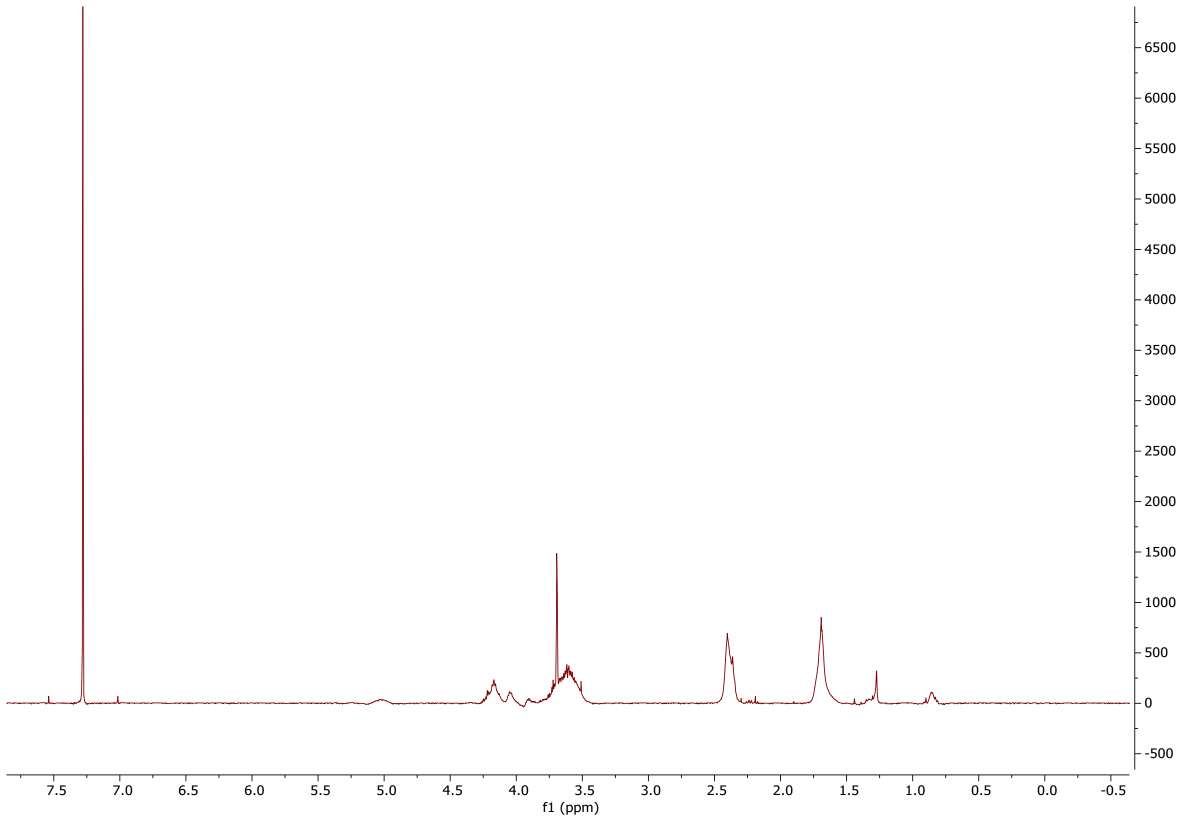

Figure S39. ^{1}H NMR spectrum of Polymer 33 in (400 MHz, $CDCl_3$).

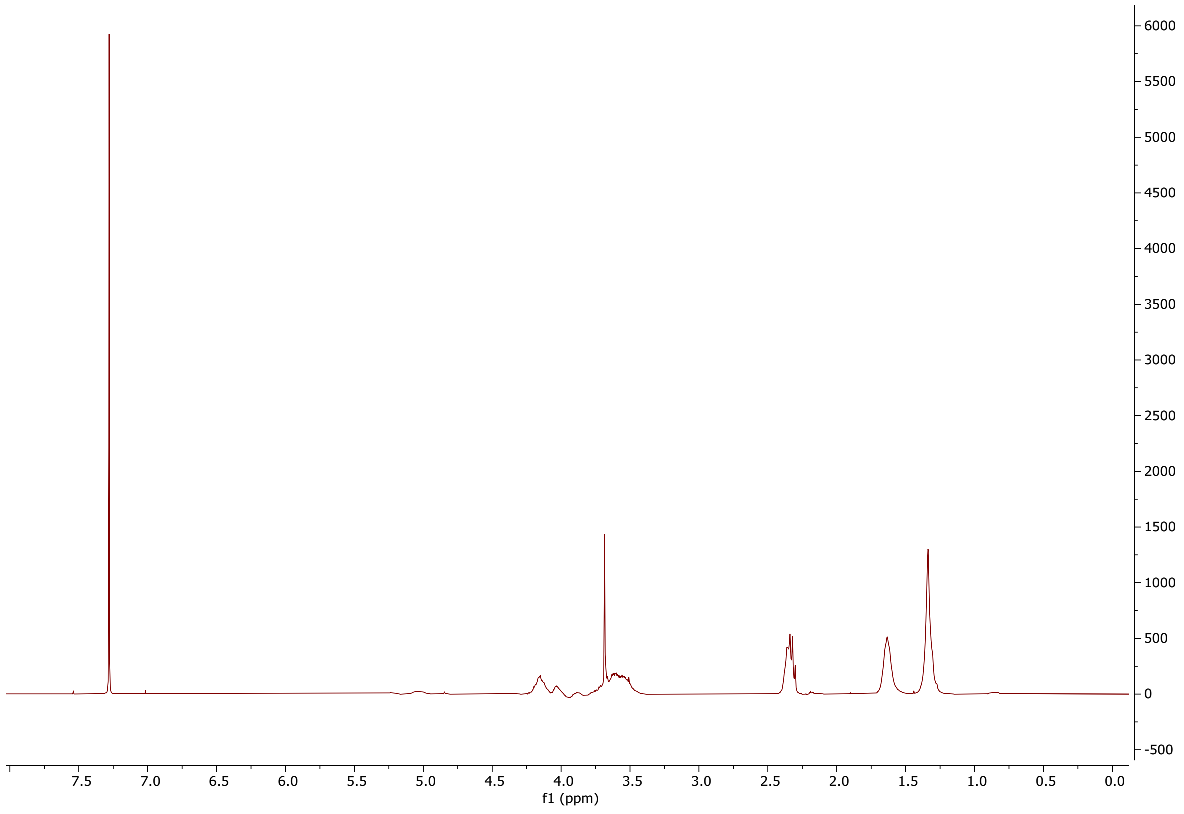

Figure S40. ^{1}H NMR spectrum of Polymer 34 in (400 MHz, $CDCl_3$).

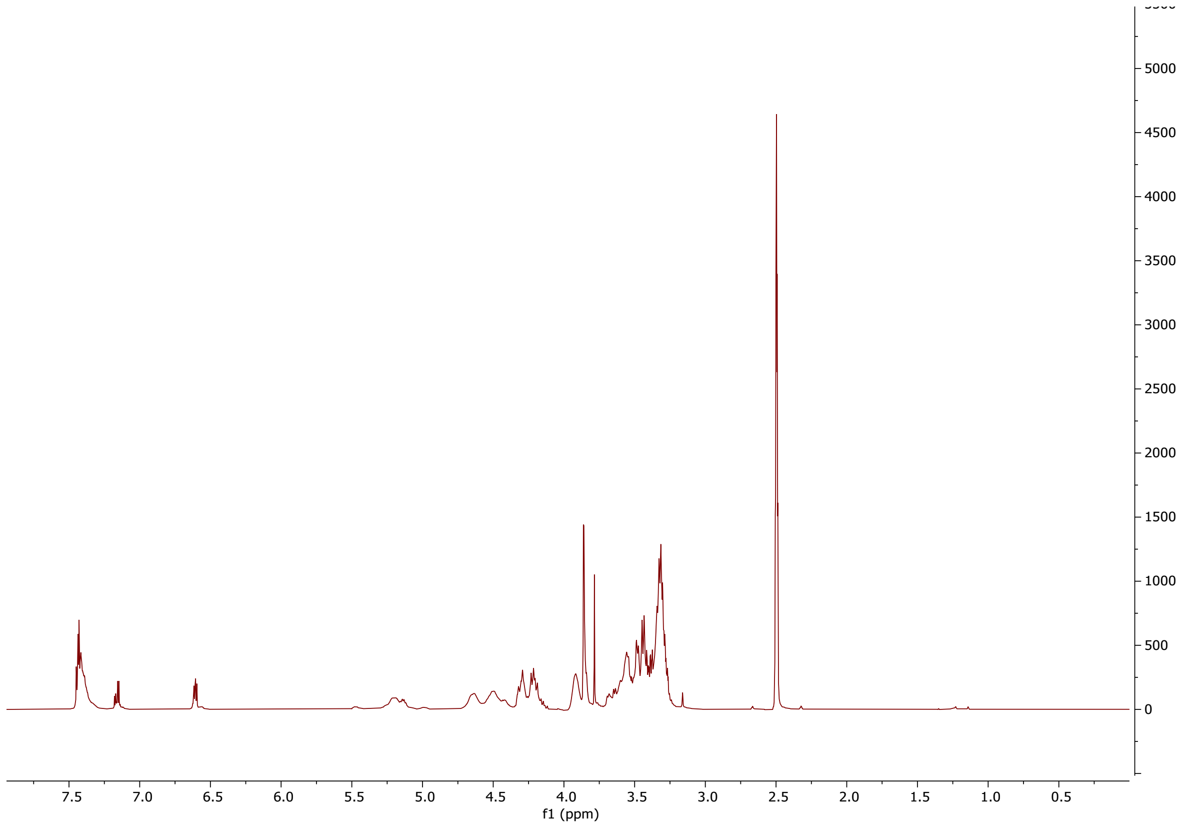

Figure S41. ^{1}H NMR spectrum of Polymer 35 (400 MHz, DMSO-d_6).

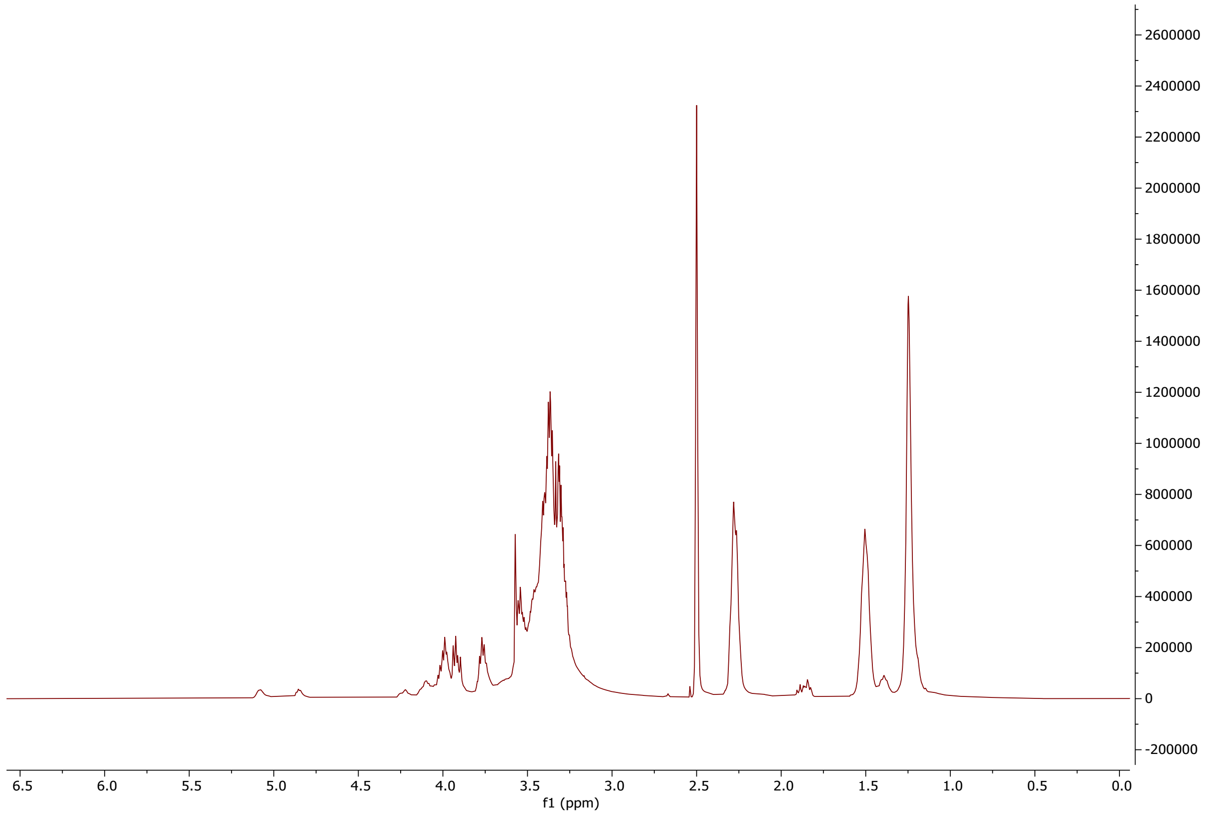

Figure S42. ^{1}H NMR spectrum of Polymer 36 (400 MHz, DMSO-d_6).

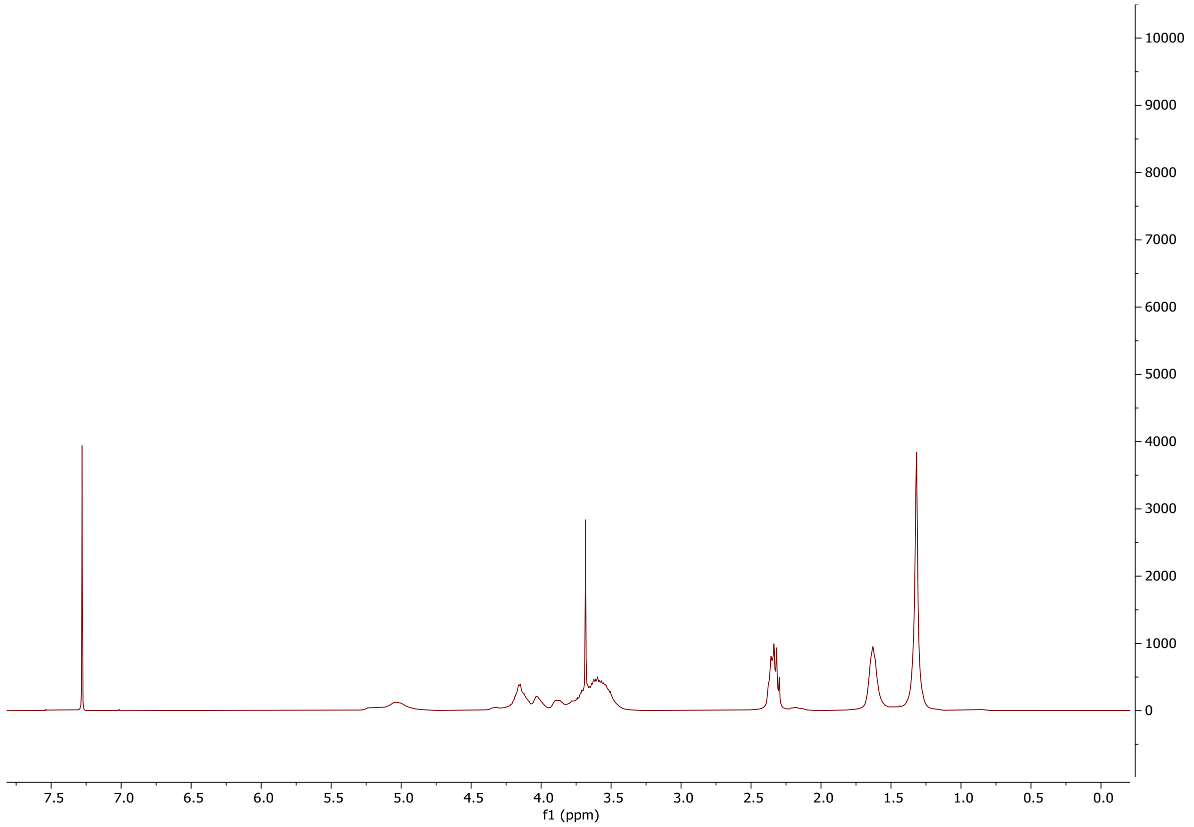

Figure S43. ^{1}H NMR spectrum of Polymer 37 in (400 MHz, $CDCl_3$).

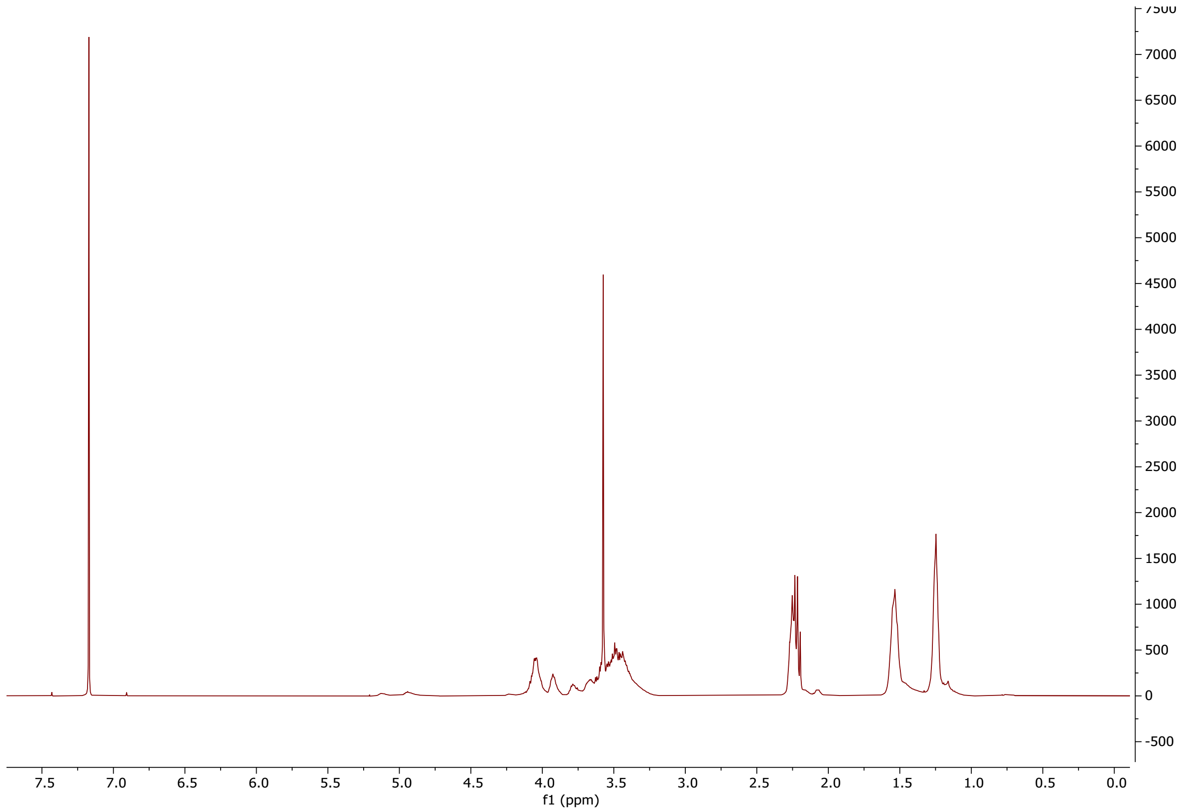

Figure S44. ^{1}H NMR spectrum of Polymer 38 in (400 MHz, $CDCl_3$).

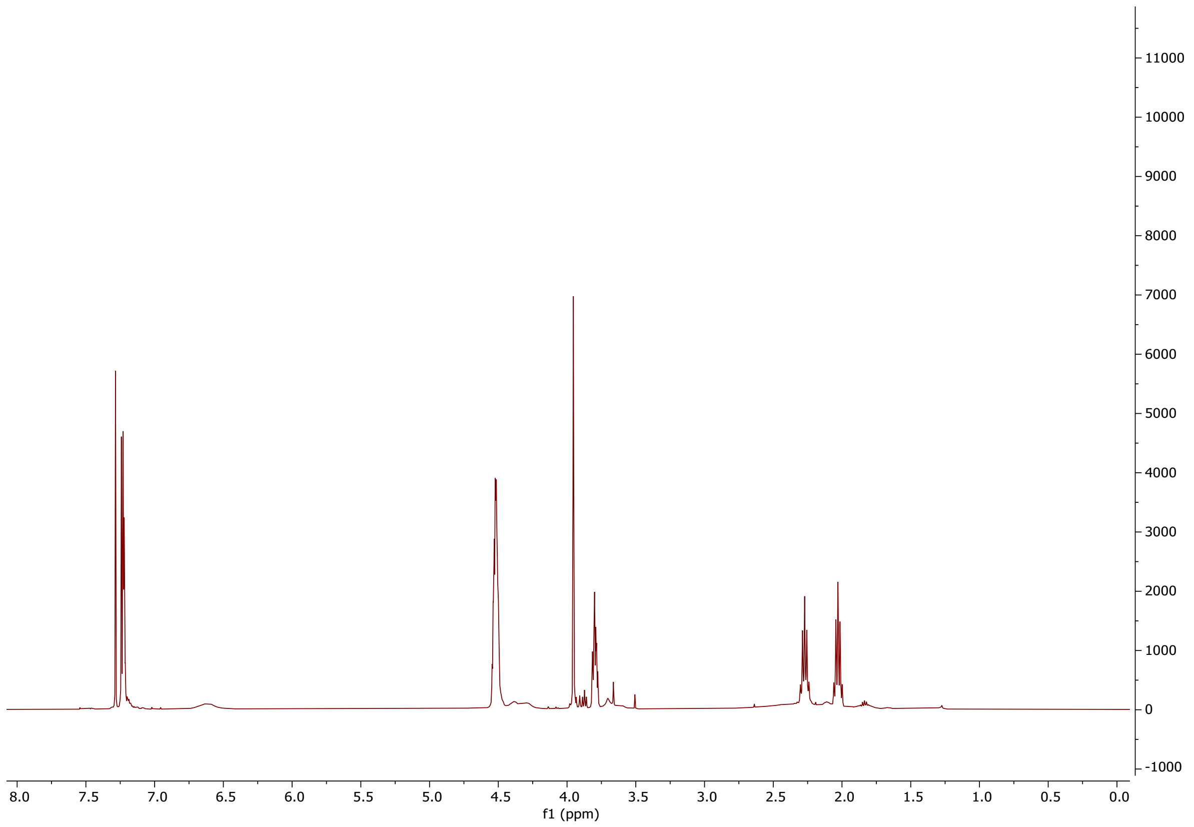

Figure S45. ^1H NMR spectrum of Polymer 39 (400 MHz, $CDCl_3$).

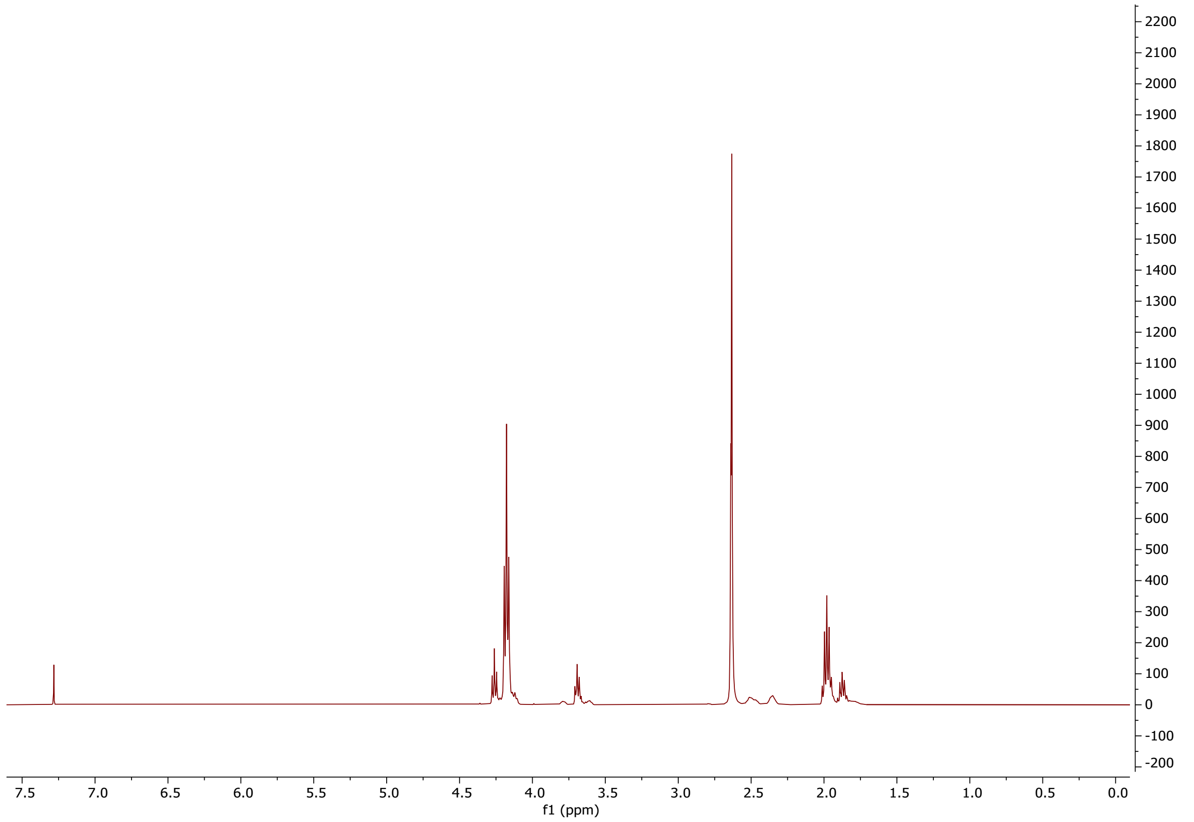

Figure S46. ^1H NMR spectrum of Polymer 40 in (400 MHz, $CDCl_3$).

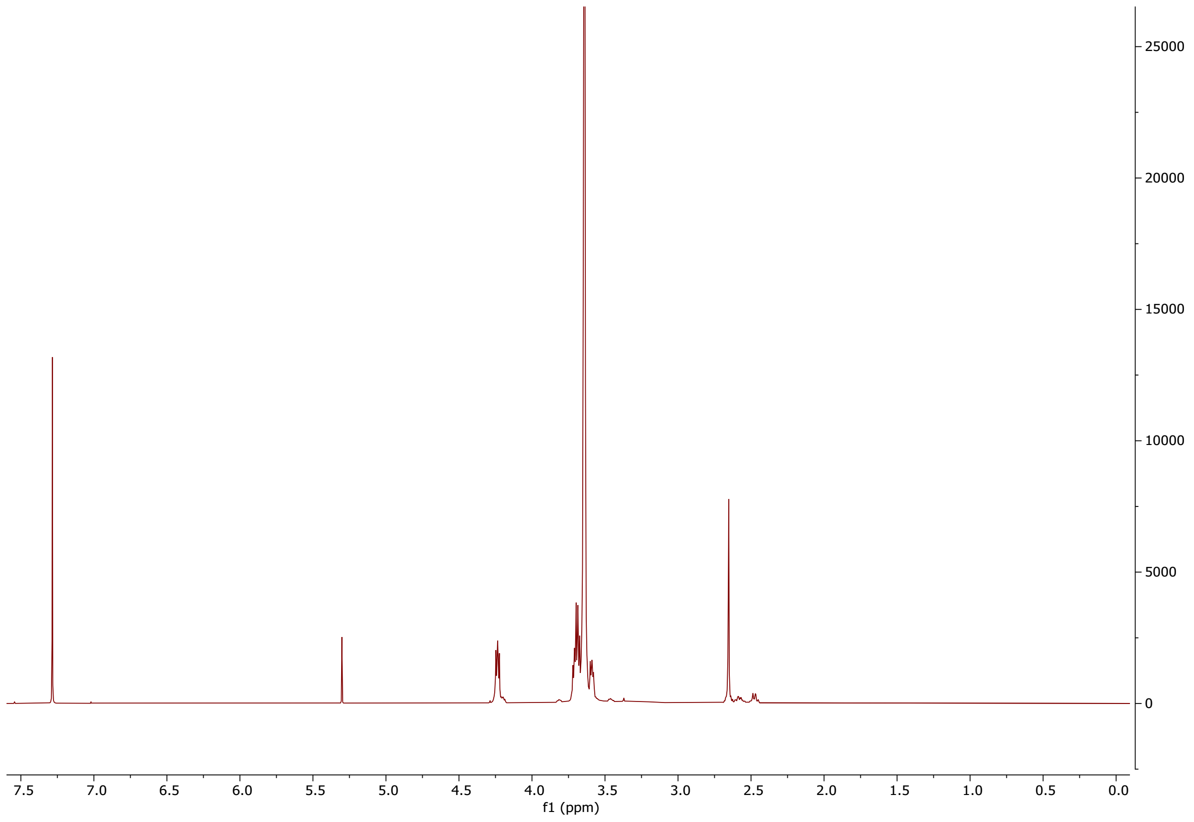

Figure S47. ^{1}H NMR spectrum of Polymer 41in (400 MHz, $CDCl_3$).

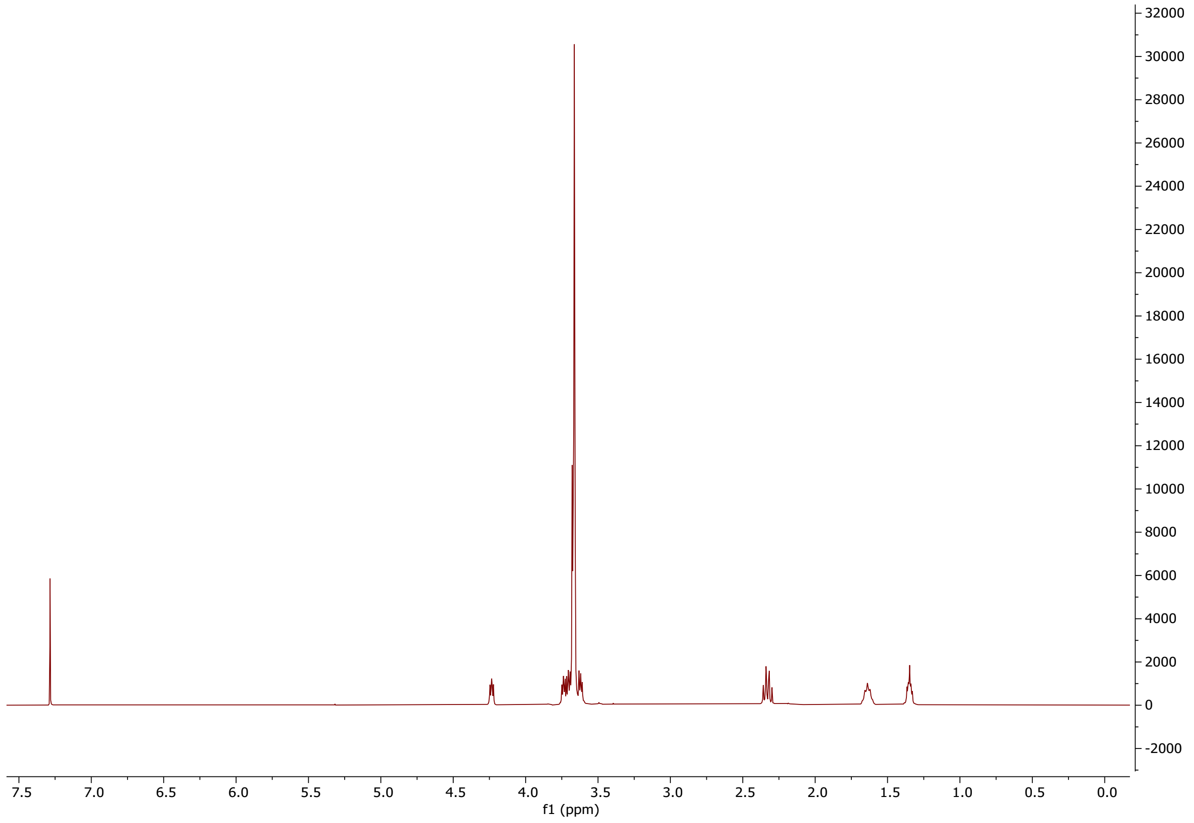

Figure S48. ^{1}H NMR spectrum of Polymer 42 in (400 MHz, $CDCl_3$).

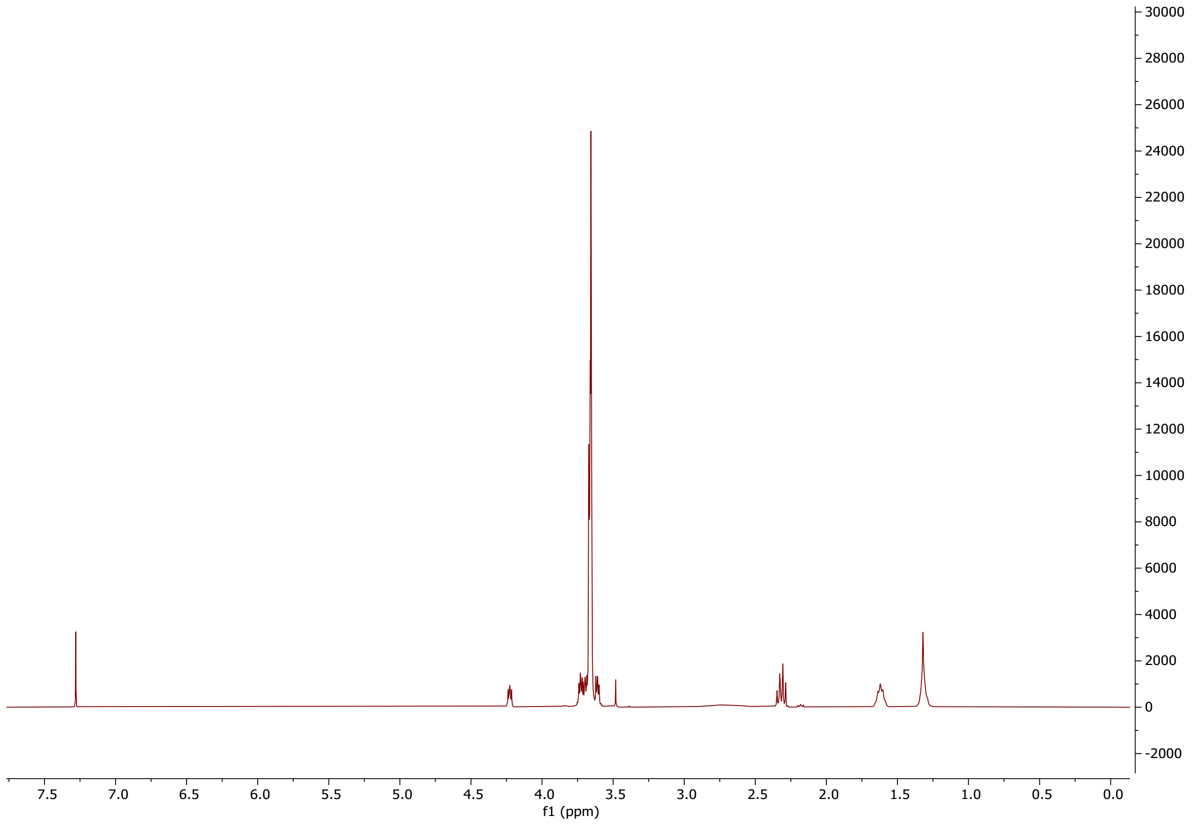

Figure S49. ^{1}H NMR spectrum of Polymer 43 in (400 MHz, $CDCl_3$).

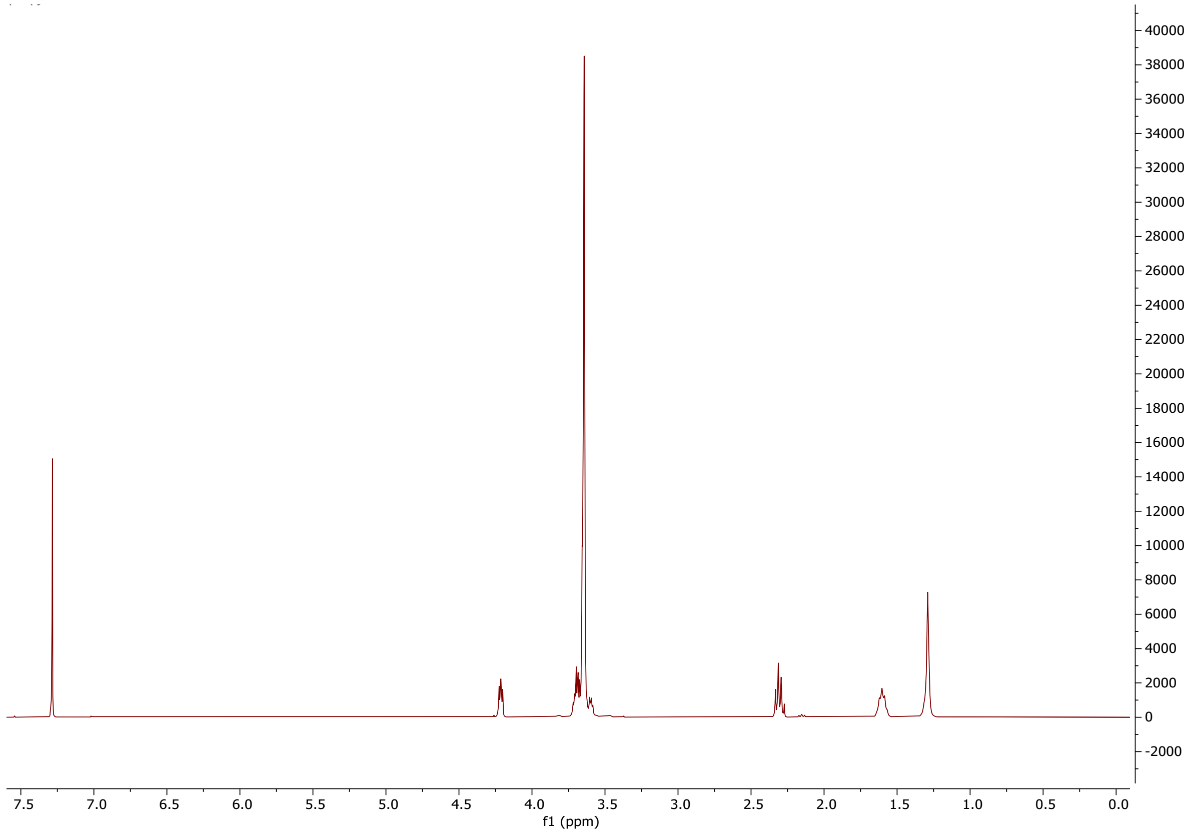

Figure S50. ^{1}H NMR spectrum of Polymer 44 in (400 MHz, $CDCl_3$).

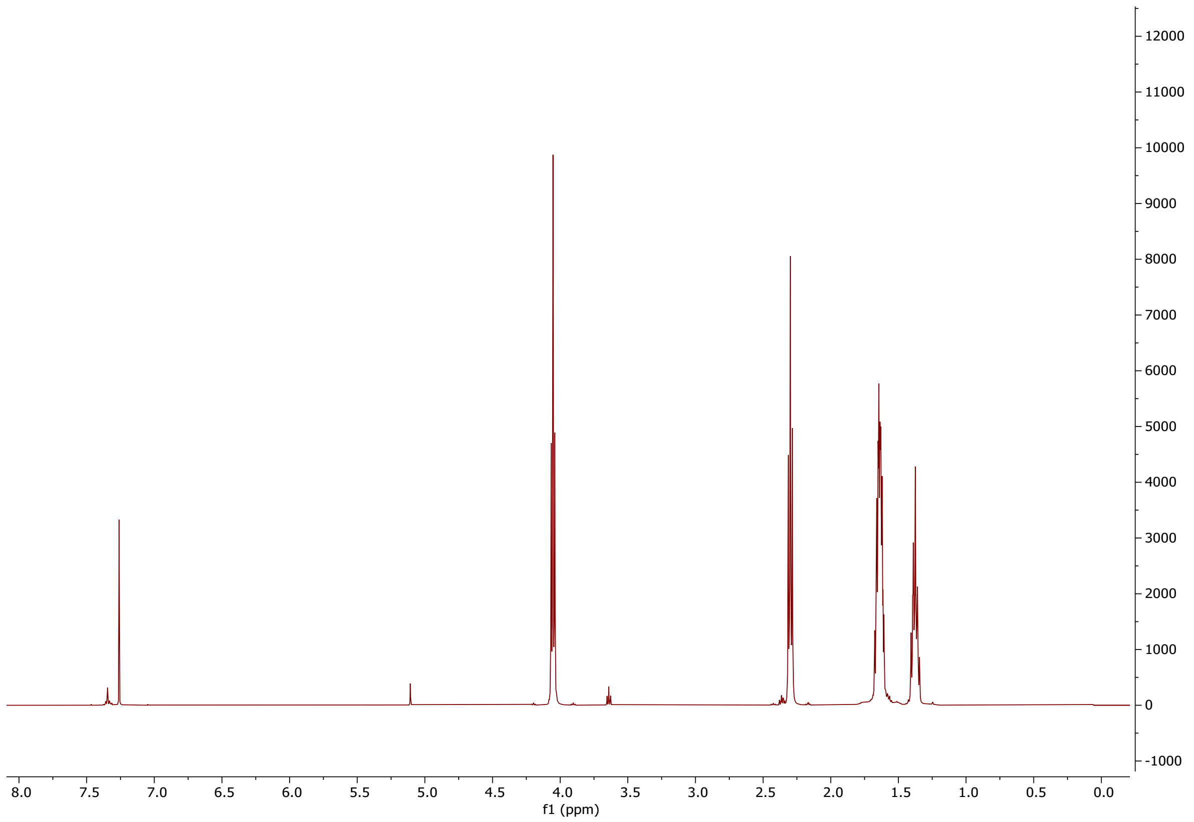

Figure S51. ^{1}H NMR spectrum of Polymer 45 in (400 MHz, $CDCl_3$).

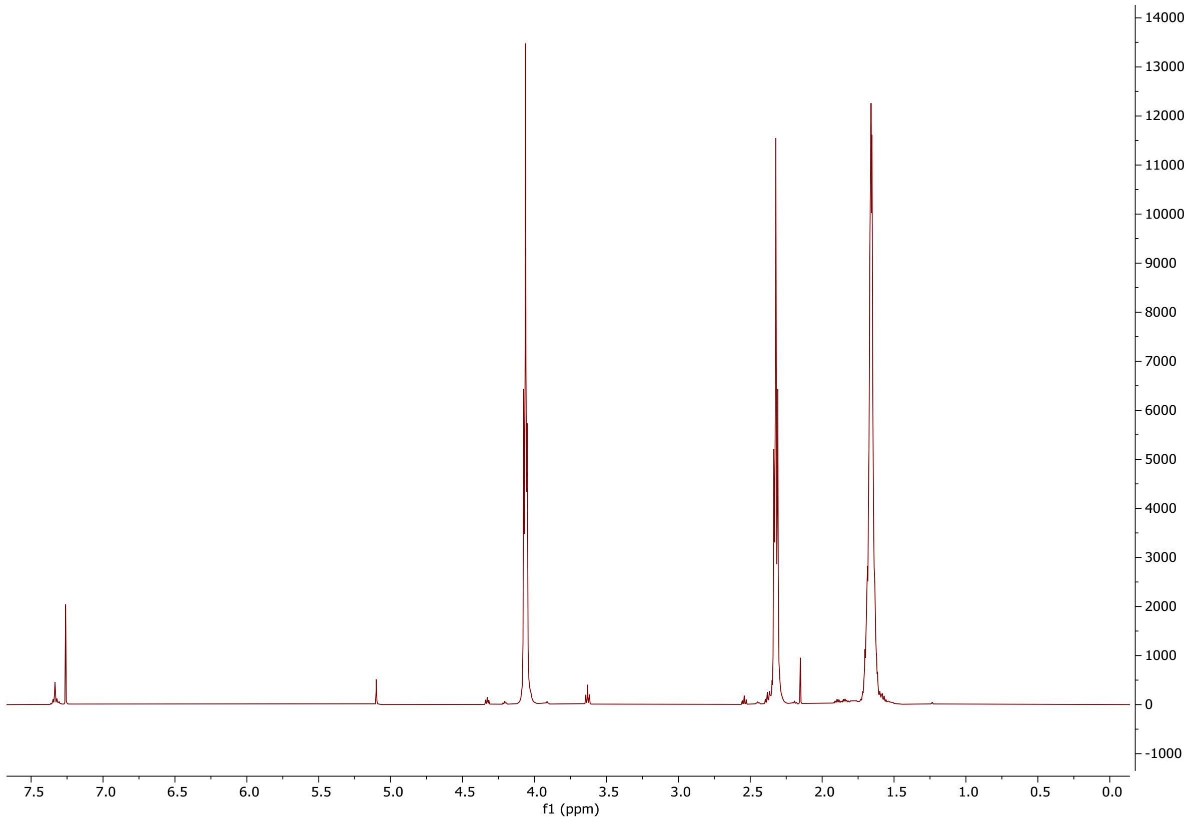

Figure S52. ^{1}H NMR spectrum of Polymer 46 in (400 MHz, $CDCl_3$).

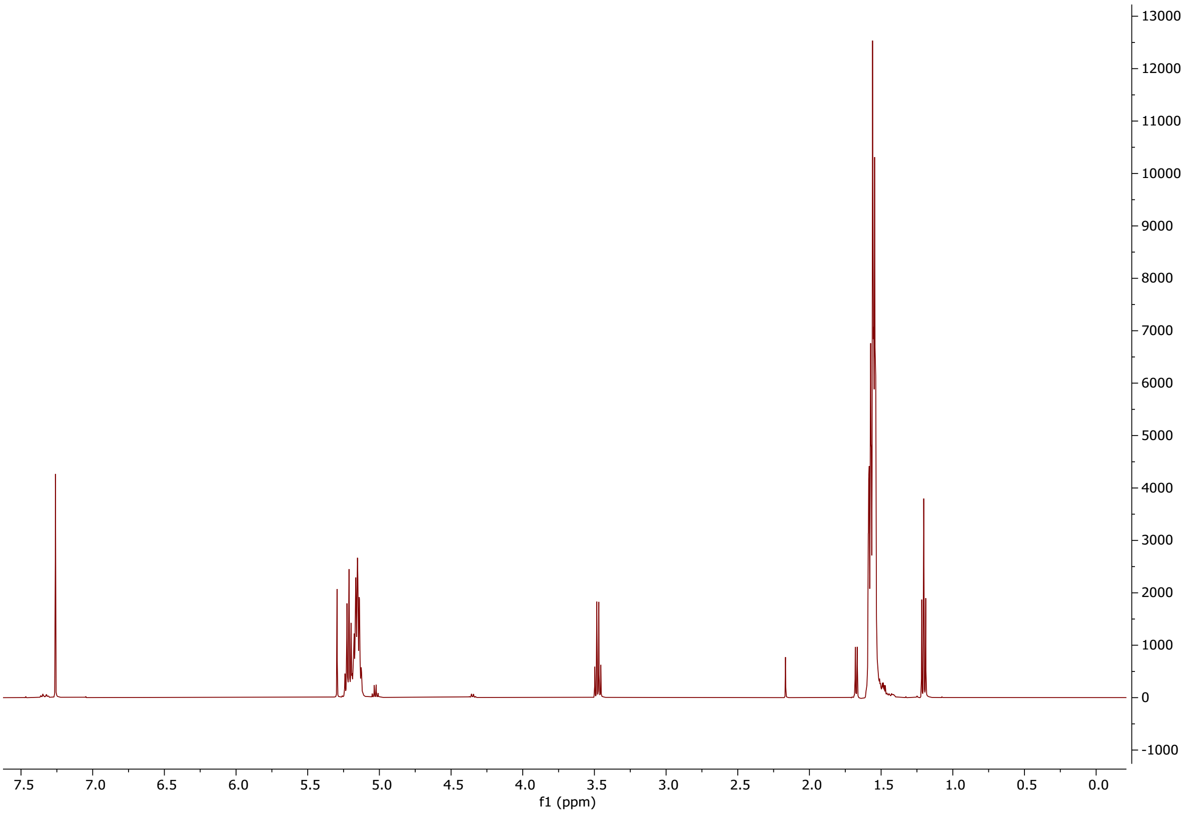

Figure S53. ^{1}H NMR spectrum of Polymer 47 in (400 MHz, $CDCl_3$).

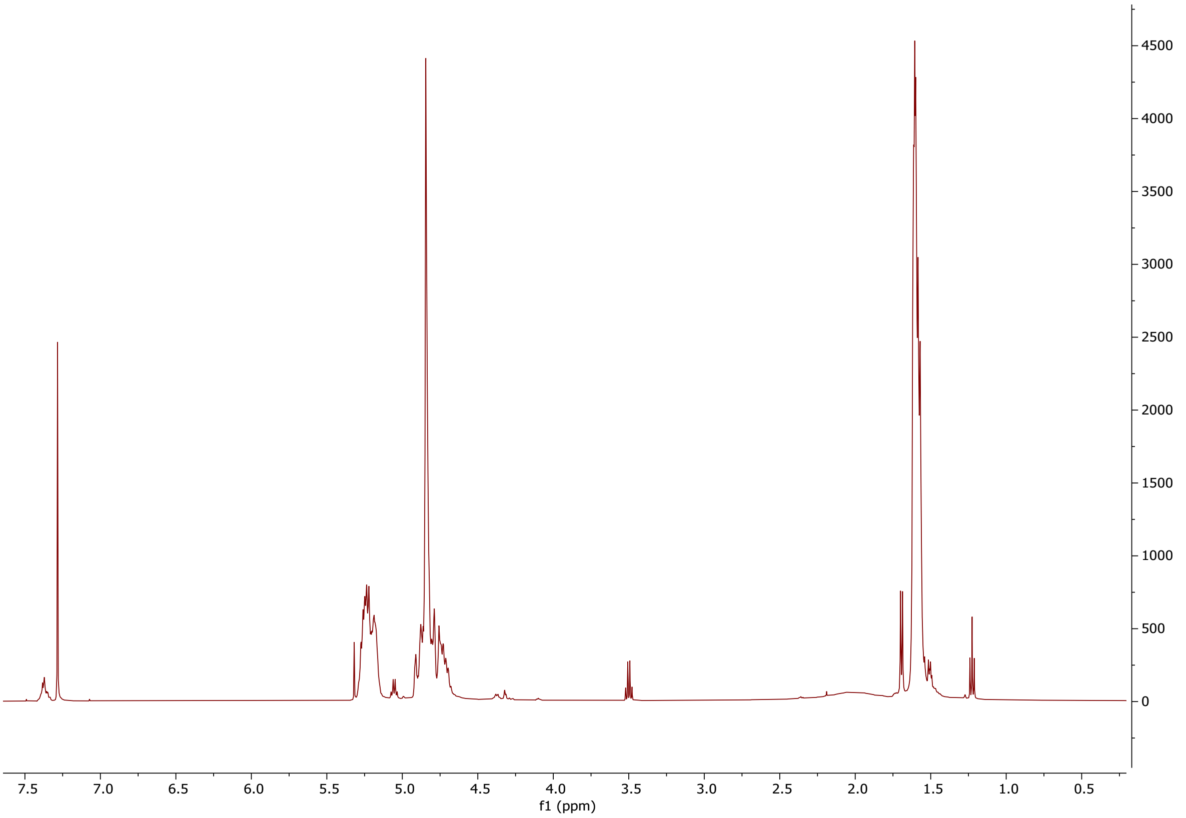

Figure S54. ^{1}H NMR spectrum of Polymer 48 in (400 MHz, $CDCl_3$).

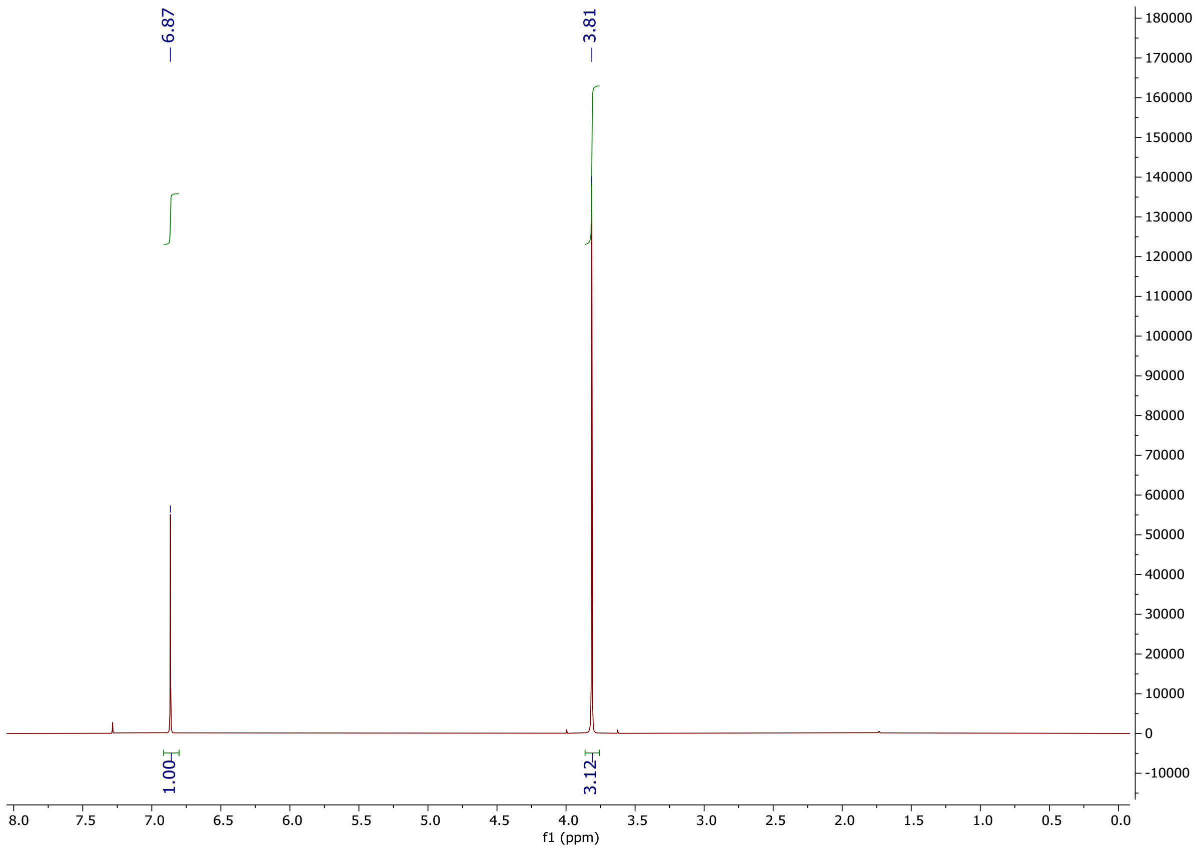

Figure S55. ^1H NMR spectrum of dimethyl fumarate (400 MHz, $CDCl_3$).

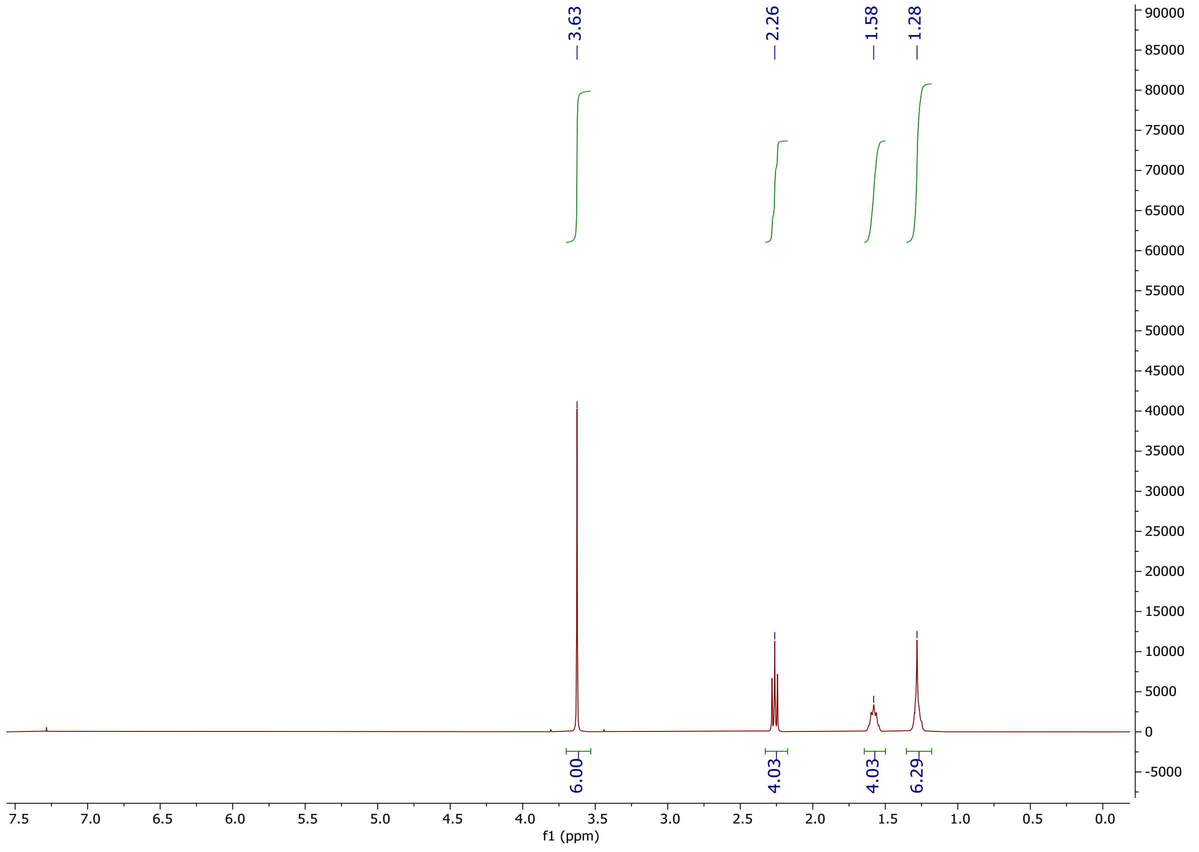

Figure S56. ^1H NMR spectrum of dimethyl azelate (400 MHz, $CDCl_3$).

Table S8. Synthetic details for the preparation of prospective polyesters via polycondensation.

Number	Diol	Diol (mmol)	Diester	Diester (mmol)	K_2CO_3 (mg)	T (°C)	Predicted biodegradability	Measured biodegradability
**A1**	1,10-decanediol	4.94	Dimethyl adipate	4.94	9	120	False	False
**A2**	1,6-hexanediol	4.94	Dimethyl suberate	4.94	8	120	False	True
**A3**	2,2-dimethyl-1,3-propanediol	4.94	Dimethyl furan-2,5-dicarboxylate	4.94	7	120	True	False
**A4**	1,2-propanediol	4.94	Dimethyl furan-2,5-dicarboxylate	4.94	6	120	True	False
**A5**	3-methyl-1,5-pentanediol	4.94	Dimethyl furan-2,5-dicarboxylate	4.94	7	120	False	False
**A6**	PEG400	4.94	Dimethyl glutarate	4.94	14	120	True	False
**A7**	glycerol	4.94	Dimethyl glutarate	4.94	6	120	True	False
**A9**	diethylene glycol	4.94	Dimethyl suberate	4.94	8	120	True	True
**A11**	diglycerol	4.94	Dimethyl glutarate	4.94	8	120	True	False
**A12**	xylitol	19.71	Dimethyl succinate	19.71	29	120	False	False

Table S9. Prospective polymer molar masses.

Number	*Eluent*	M_n (g/mol)	M_w (g/mol)	$Đ$
**A1**	THF	2860	4530	1.6
**A2**	THF	3350	5120	1.5
**A3**	THF	1060	1400	1.3
**A4**	THF	800	1030	1.3
**A5**	THF	2620	3420	1.3
**A6**	THF	2600	4470	1.7
**A7**	-	n.d.	n.d.	n.d.
**A9**	-	n.d.	n.d.	n.d.
**A11**	Water	6170	14170	2.3
**A12**	-	n.d.	n.d.	n.d.

* Samples A7, A9 and A12 were not soluble in GPC eluents; therefore, it was not possible to determine their molar masses. These samples are marked with n.d.

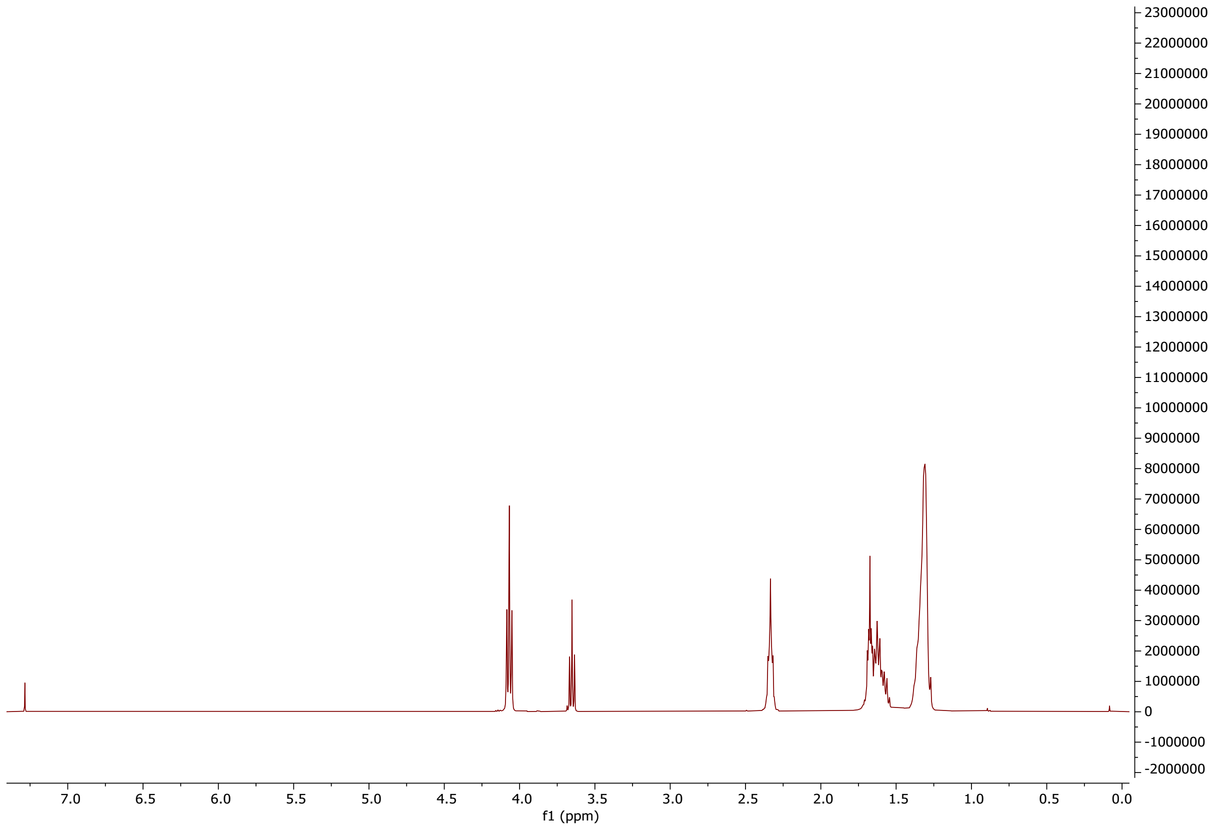

Figure S57. ^{1}H NMR spectrum of Polymer A1 (400 MHz, $CDCl_3$).

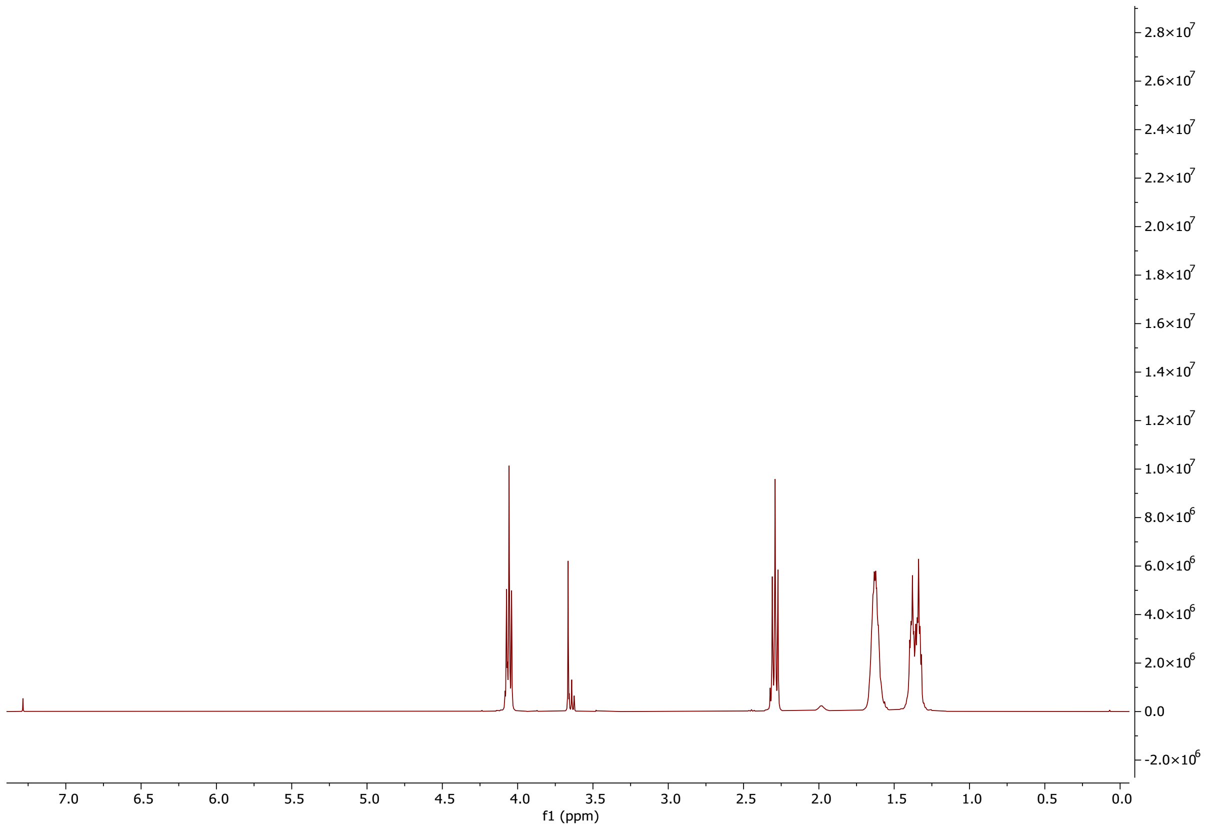

Figure S58. ^{1}H NMR spectrum of Polymer A2 (400 MHz, $CDCl_3$).

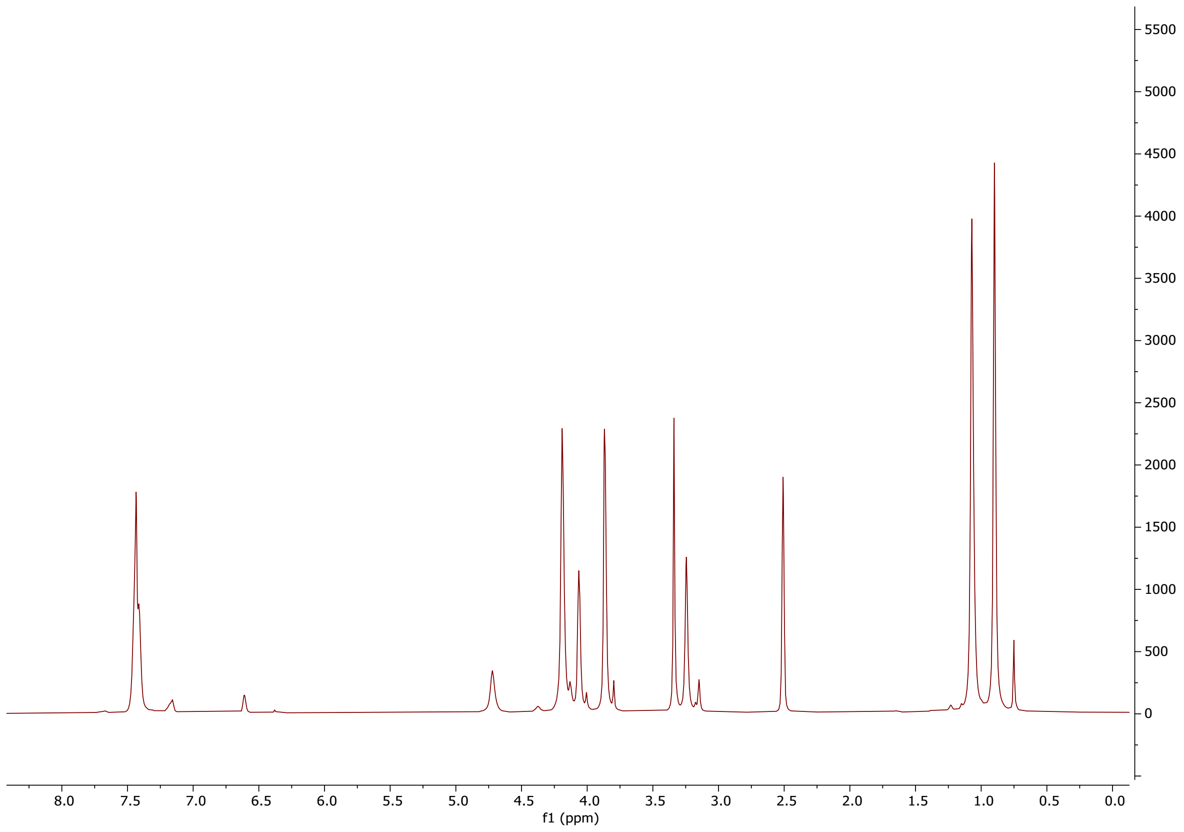

Figure S59. ^{1}H NMR spectrum of Polymer A3 (400 MHz, DMSO-d_6).

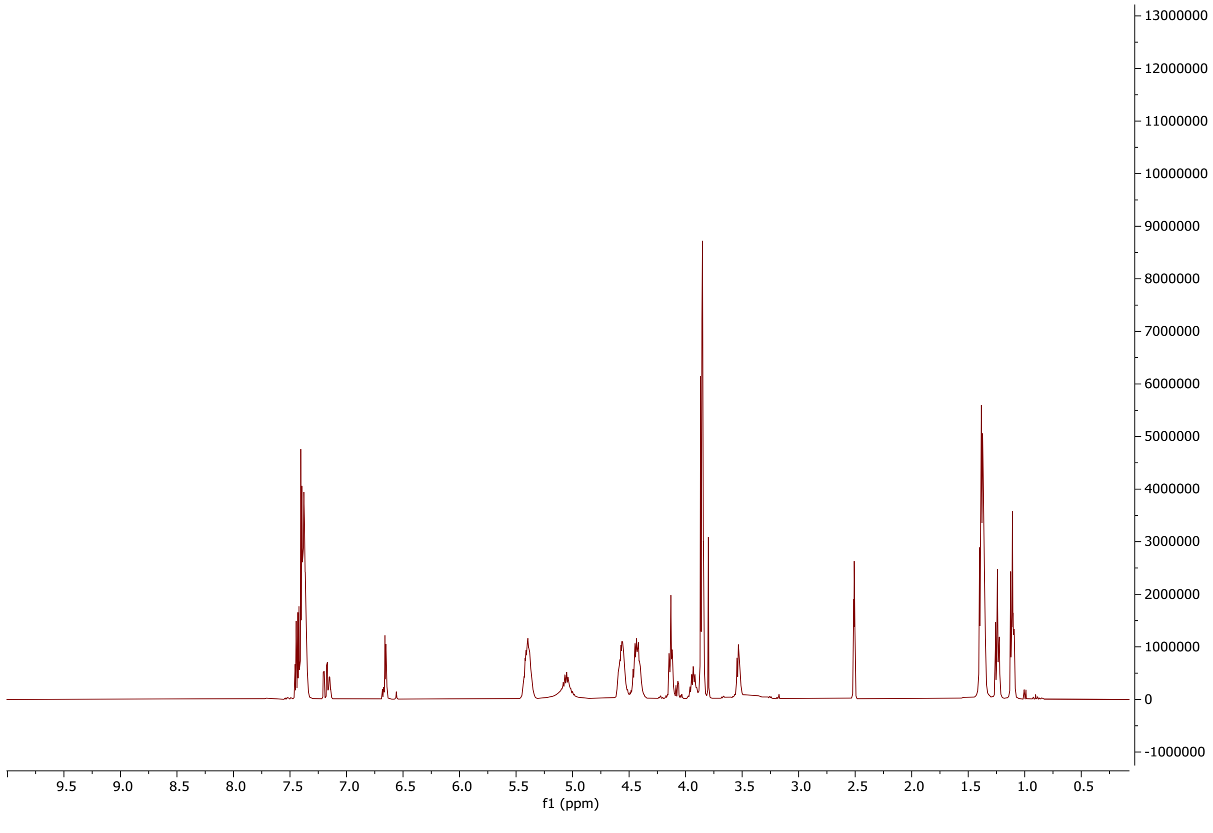

Figure S60. ^{1}H NMR spectrum of Polymer A4 (400 MHz, DMSO-d_6).

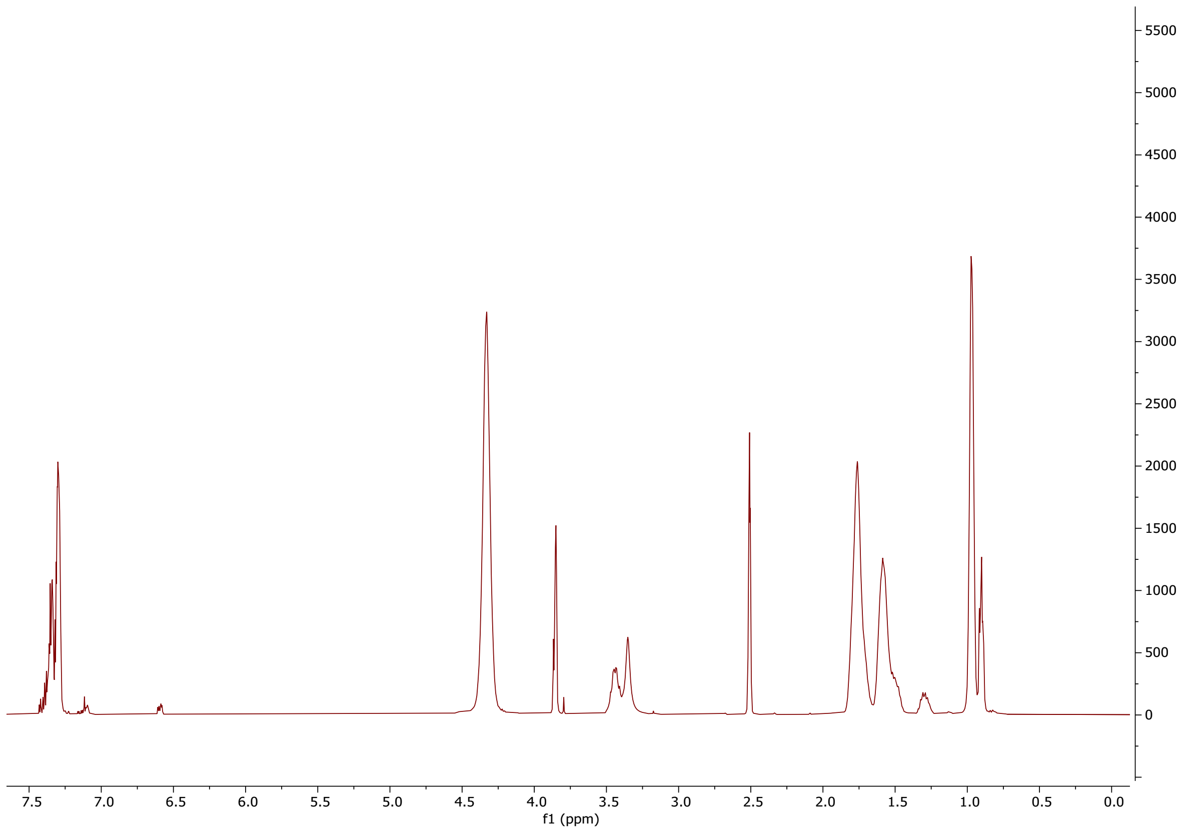

Figure S61. ^{1}H NMR spectrum of Polymer A5 (400 MHz, DMSO-d_6).

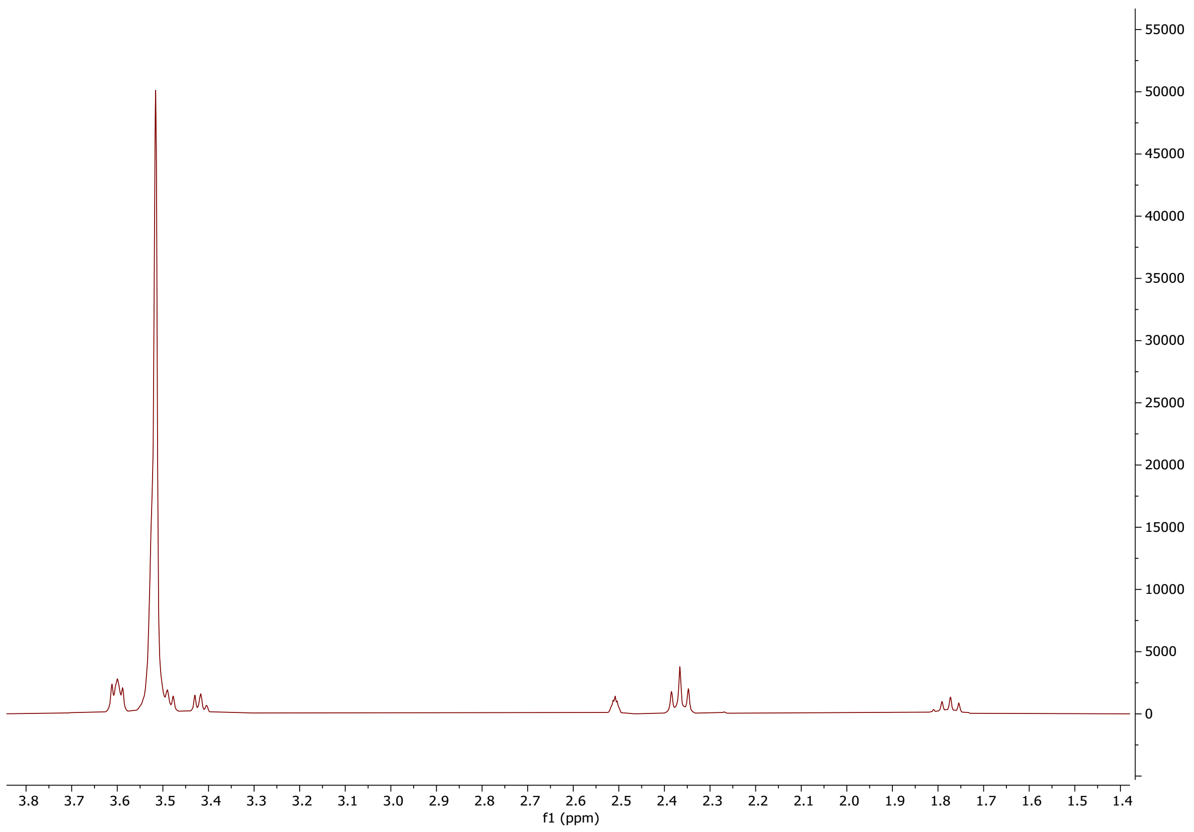

Figure S62. ^{1}H NMR spectrum of Polymer A6 (400 MHz, DMSO-d_6).

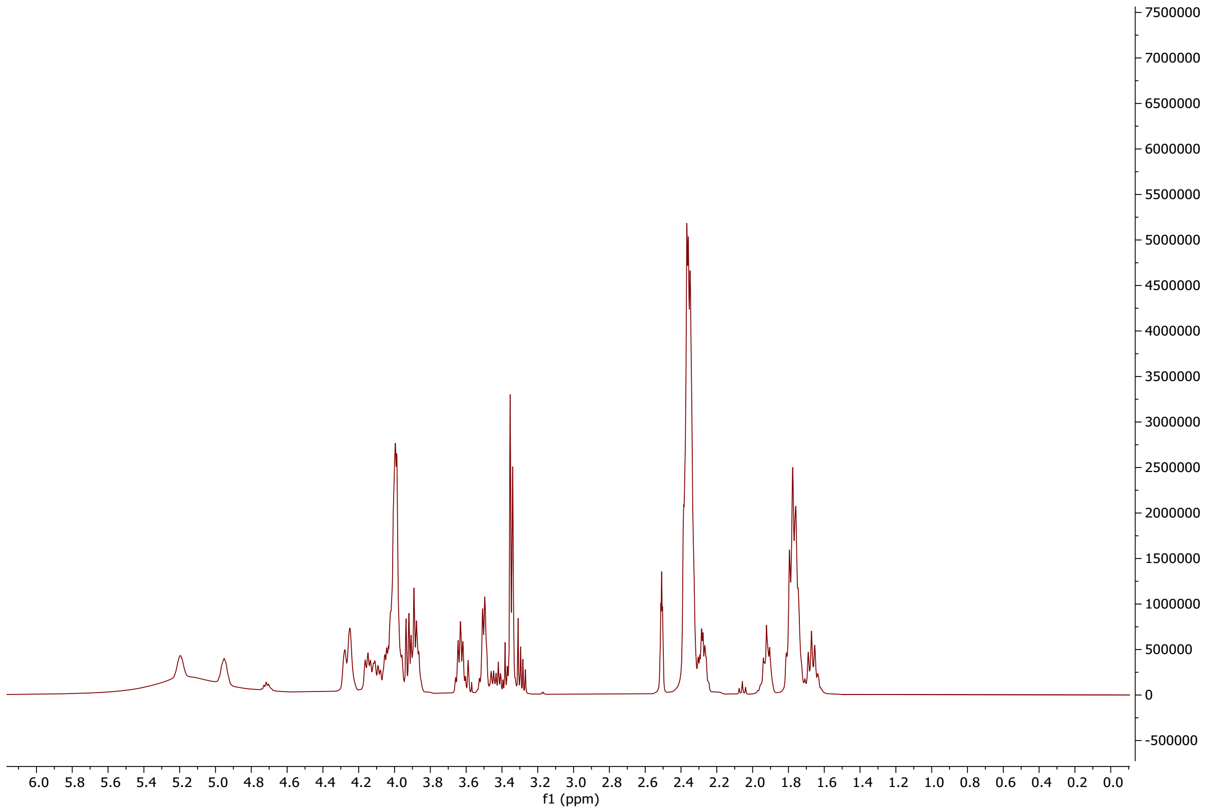

Figure S63. ^{1}H NMR spectrum of Polymer A7 (400 MHz, DMSO-d_6).

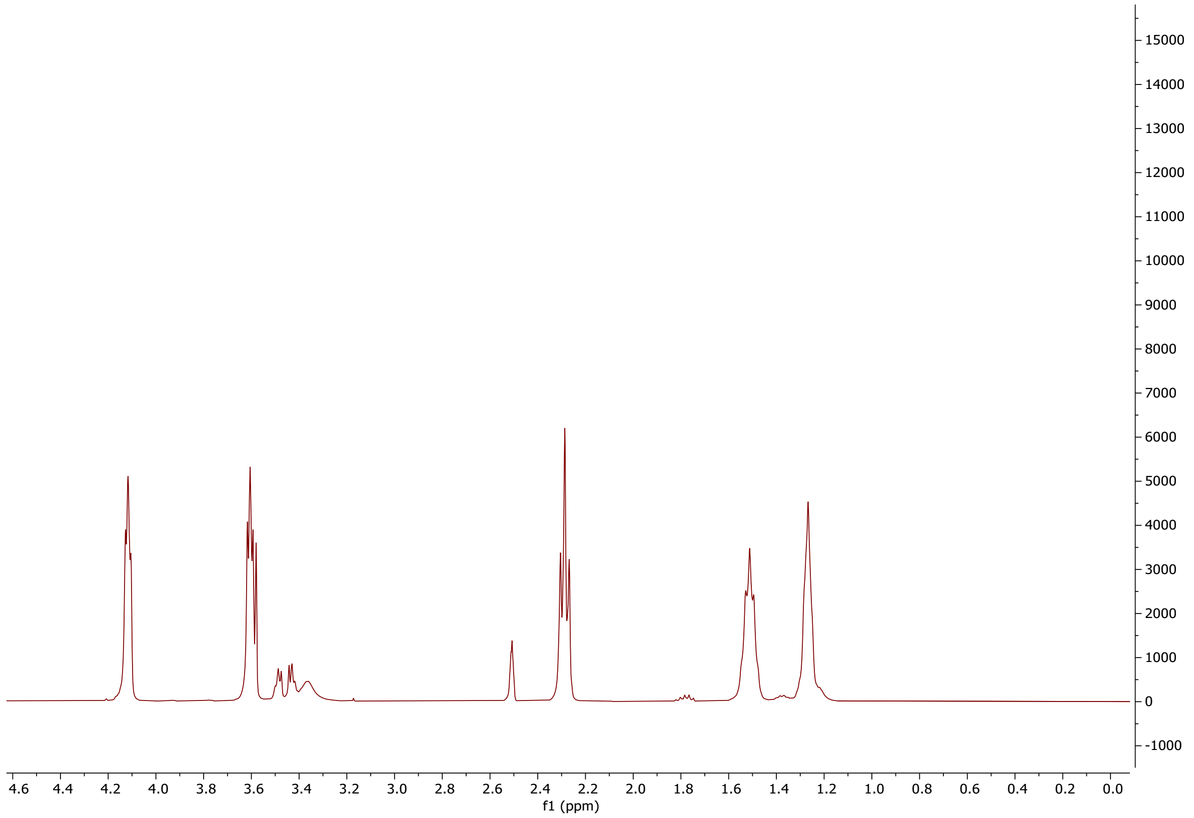

Figure S64. ^{1}H NMR spectrum of Polymer A9 (400 MHz, DMSO-d_6).

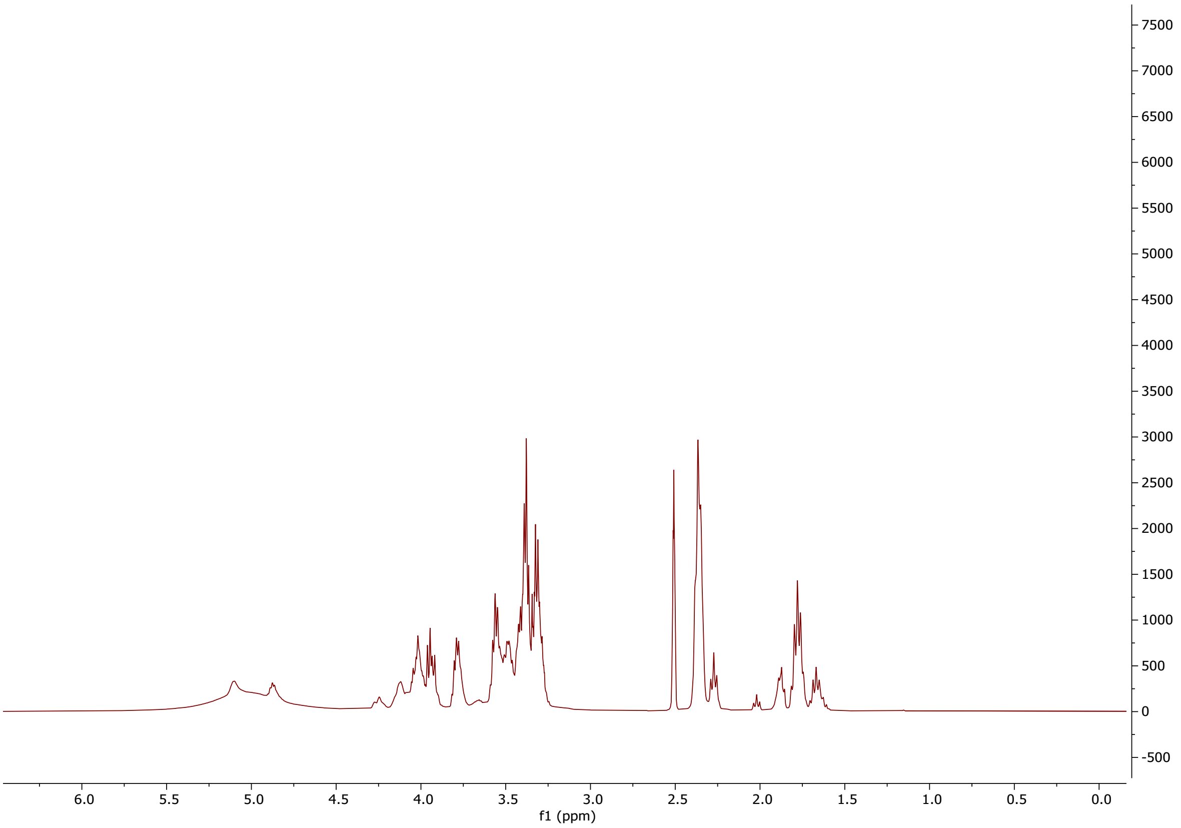

Figure S65. ^{1}H NMR spectrum of Polymer A11 (400 MHz, DMSO-d_6).

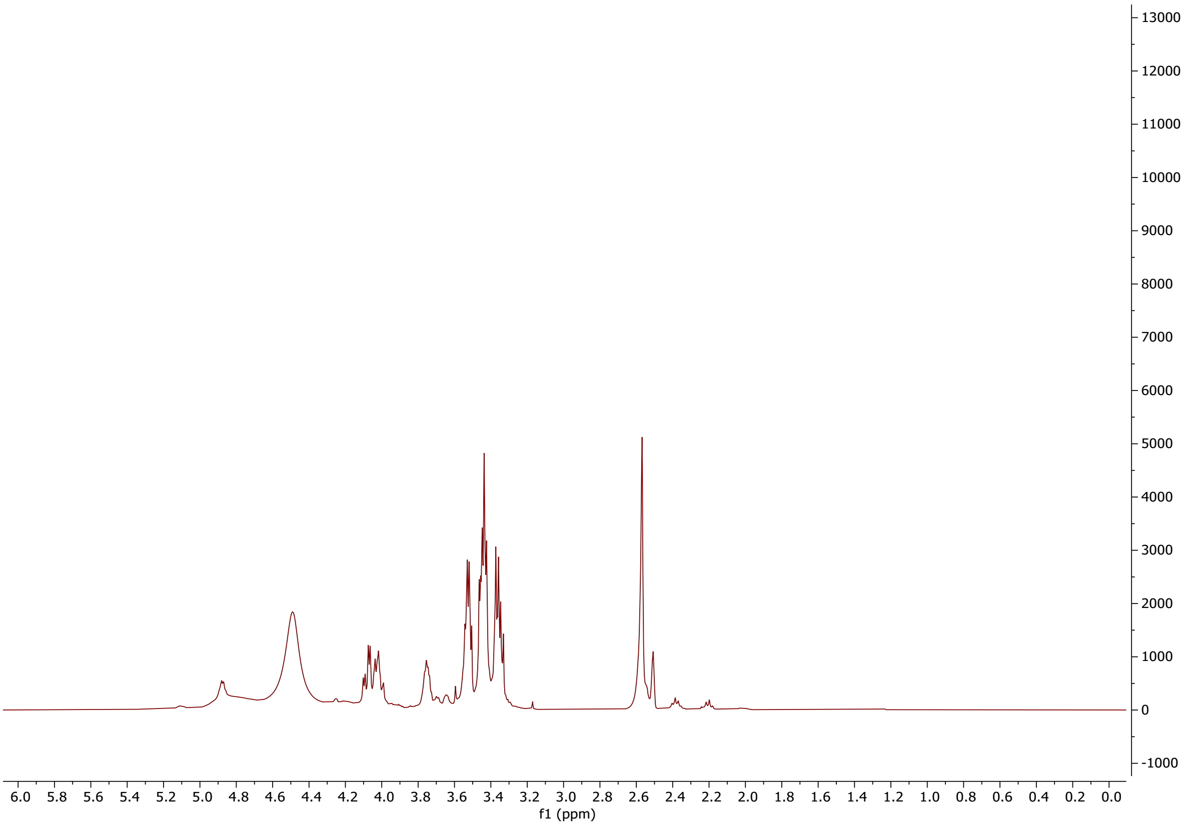

Figure S66. ^{1}H NMR spectrum of Polymer A12 (400 MHz, DMSO-d_6).

References

1. Y. M. Chung, C. K. Wei, D. W. Chuang, M. El-Shazly, C. T. Hsieh, T. Asai, Y. Oshima, T. J. Hsieh, T. L. Hwang, Y. C. Wu and F. R. Chang, *Bioorg. Med. Chem.*, 2013, **21**, 3866-3872.
2. K. A. Fransen, S. H. M. Av-Ron, T. R. Buchanan, D. J. Walsh, D. T. Rota, L. Van Note and B. D. Olsen, *Proceedings of the National Academy of Sciences of the United States of America*, 2023, **120**, e2220021120.
3. S. A. d. Silva, D. J. L. Faccin and N. S. M. Cardozo, *ACS Sustainable Chemistry & Engineering*, August 1, 2024, **12**.
4. D. Alvarez-Melis and N. Fusi, presented in part at the Proceedings of the 34th International Conference on Neural Information Processing Systems, Vancouver, BC, Canada, 2020.
5. D. Alvarez-Melis and N. Fusi, presented in part at the Proceedings of the 38th International Conference on Machine Learning, Proceedings of Machine Learning Research, 2021.
6. Microsoft, Microsoft/otdd, https://github.com/microsoft/otdd, (accessed 6th November, 2025).
